# Benchmark Study of the Performance of Density Functional Theory for Bond Activations with (Ni,Pd)-Based Transition-Metal Catalysts

**DOI:** 10.1002/open.201300012

**Published:** 2013-06-03

**Authors:** Marc Steinmetz, Stefan Grimme

**Affiliations:** [a] Mulliken Center for Theoretical Chemistry, Institut für Physikalische und Theoretische Chemie der Universität BonnBeringstr. 4, 53115 Bonn (Germany)

**Keywords:** ab initio calculations, bond activation, density functional calculations, dispersion correction, transition metals

## Abstract

The performance of 23 density functionals, including one LDA, four GGAs, three meta-GGAs, three hybrid GGAs, eight hybrid meta-GGAs, and ten double-hybrid functionals, was investigated for the computation of activation energies of various covalent main-group single bonds by four catalysts: Pd, PdCl^−^, PdCl_2_, and Ni (all in the singlet state). A reactant complex, the barrier, and reaction energy were considered, leading to 164 energy data points for statistical analysis. Extended Gaussian AO basis sets were used in all calculations. The best functional for the complete benchmark set relative to estimated CCSD(T)/CBS reference data is PBE0-D3, with an MAD value of 1.1 kcal mol^−1^ followed by PW6B95-D3, the double hybrid PWPB95-D3, and B3LYP-D3 (1.9 kcal mol^−1^ each). The other tested hybrid meta-GGAs perform less well (M06-HF: 7.0 kcal mol^−1^; M06-2X: 6.3 kcal mol^−1^; M06: 4.9 kcal mol^−1^) for the investigated reactions. In the Ni case, some double hybrids show larger errors due to partial breakdown of the perturbative treatment for the correlation energy in cases with difficult electronic structures (partial multi-reference character). Only double hybrids either with very low amounts of perturbative correlation (e.g., PBE0-DH) or that use the opposite-spin correlation component only (e.g., PWPB95) seem to be more robust. We also investigated the effect of the D3 dispersion correction. While the barriers are not affected by this correction, significant and mostly positive results were observed for reaction energies. Furthermore, six very recently proposed double-hybrid functionals were analyzed regarding the influence of the amount of Fock exchange as well as the type of perturbative correlation treatment. According to these results, double hybrids with <50–60 % of exact exchange and ∼30 % perturbative correlation perform best.

## Introduction

Over the past decade Kohn–Sham density functional theory (DFT)^1^–^5^ has become a very important tool for understanding mechanistic problems in chemistry. Evaluation of the performance of density functionals (DFs) by benchmarking for different realistic chemical tasks is a crucial step prior to the investigation of new problems. Several sets were developed in recent years to test DFs, such as atomization energies,^6^–^8^ noncovalent interactions,^9^–^12^ and thermochemistry and kinetics.^13^–^15^ Many of these were collected in the GMTKN30^16^ test set by our group to build a large benchmark, which includes a thorough treatment of the chemically important main-group chemistry.

Less extensive benchmarks exist in the field of transition-metal chemistry. Truhlar and co-workers used small transition-metal compounds to benchmark bond energies^17^, ^18^ and s/d excitation energies^19^ with several DFs. They also developed new DFs based on different training sets partly containing transition metals^20^–^24^ and tested the behavior of several effective core potentials.^25^ Jiang et al. studied conventional and double-hybrid density functionals (DHDFs) for the thermochemistry of the ccCATM/11 test set, building on 193 molecules containing 3d-transition metals.^26^ The best functionals in this study were B97-1^27^ and mPW2PLYP^28^ compared with experimental data. Hughes et al. recently presented a special dispersion correction for ligand-removal enthalpies of transition-metal complexes.^29^

An area of great interest in chemistry is bond activation by transition-metal catalysts as studied by Siegbahn and Blomberg during the 1980s and 1990s. They investigated the influence of the transition metal on the oxidative addition into different types of bonds based on several ab initio methods.^30^–^41^ Bickelhaupt and co-workers later analyzed reactions of different palladium catalysts with various hydrocarbons.^42^–^47^ The main focus was to show the limits of wave-function methods, basis set effects, and the role of relativistic effects in treating organometallic reactions. In these studies a small number of DFs were included for comparison.

In 2006 Quintal et al. published a benchmark set concentrating on the performance of DFT against reliable theoretical reference data.^48^ They used the palladium reactions from the investigations of Bickelhaupt and co-workers and tested several DFs against extrapolated CCSD(T) values. The main conclusion was that there is no “best functional,…, but rather a cluster of several functionals”. In general, these are hybrids with ∼20 % Fock exchange, for example, PBE0^49^ or PW6B95.^50^ Lai et al. recently benchmarked twelve DFs for C–H activation energies of methane by pincer complexes of late platinum group metals.^51^ Their findings are that the best DF is B3LYP,^52^, ^53^ a dispersion correction has no influence on the activation energies, and palladium-based pincer complexes are relatively complicated for DFT methods. Oyedepo and Wilson^54^ investigated C–C bond activation by various transition metals for the model system ethane and a chemically more realistic example (lignine model), with several methods against CCSD(T)/CBS values including three DHDFs (B2PLYP,^55^ B2GPPLYP,^56^ and mPW2PLYP^28^). This study reveals that the best method for the reaction energies is B2PLYP, whereas M06 shows the best performance for activation barriers.

Because there is only a relatively small number of benchmarks with transition-metal-based insertion reactions, we compiled a large test set including complexation energies, reaction energies, and, most importantly, reaction barriers with three palladium catalysts in two different oxidation states (Pd, PdCl^−^, and PdCl_2_). Some of the palladium-based reactions had already been used in a previous smaller benchmark.^48^ We also generated a subset with nickel in the singlet state, which is quite challenging for single-reference methods due to some multi-reference character in the electronic wave functions. Compared with other tests, many different bond activations were included in our benchmark, namely C–H activations for all possible hybridizations of carbon, C–C, O–H, B–H, N–H, and C–Cl bond activations. This study continues previous extensive benchmark work by our group for main-group thermochemistry.^15^, ^57^–^59^

One aim of our work was to test for effects of our D3 dispersion correction^60^ on the various parts of the reactions (complexation, reaction, and barrier). For the problems investigated herein that involve only relatively small molecules, this correction mainly accounts for density-functional-dependent medium-range correlation effects and not the ‘true’ long-range dispersion energy.

We also conducted a detailed test of the class of DHDFs because it is known that these functionals are less robust for electronically very complicated systems due to the added perturbative orbital-dependent correlation to the functional. This should shed some light on general questions regarding their applicability in chemically complicated situations. Thereby, we applied well-known double-hybrid functionals, namely B2PLYP,^55^ B2GPPLYP,^56^ mPW2PLYP,^28^ and DSD-BLYP.^61^ We furthermore included the very recently developed DHDFs PWPB95, PTPSS,^16^ 1DH-BLYP, 1DH-PBE,^62^ PBE0-2,^63^ and PBE0-DH.^64^ In PWPB95 and PTPSS, only the opposite-spin part in the orbital-based correlation was considered. Together with the resolution-of-identity (RI)^65^ and the Laplace transformation algorithm,^66^ this reduces the formal scaling from *N*^5^ to *N*^4^, in which *N* is a measure of system size. DSD-BLYP is also a special case because it employs SCS-MP2^67^-type correlation instead of normal MP2.^68^

For a recent review about spin-component correlation methods, see ref. 69. For a broader perspective, standard DFs like B3LYP^52^, ^53^ are included as well.

### Selection of reactions

Inspired by the tests of Siegbahn et al.,^30^–^41^ de Jong et al.,^42^–^45^ Diefenbach et al.,^46^ and Quintal et al.,^48^ we created a test set of small prototype reactions with Pd, PdCl^−^, PdCl_2_, and Ni as catalysts and various types of bond activations, namely C–H, C–C, O–H, B–H, N–H, and C–Cl. In the case of C–H bonds, we included systems with each type of hybridization of the carbon atom.

**Figure 1:**
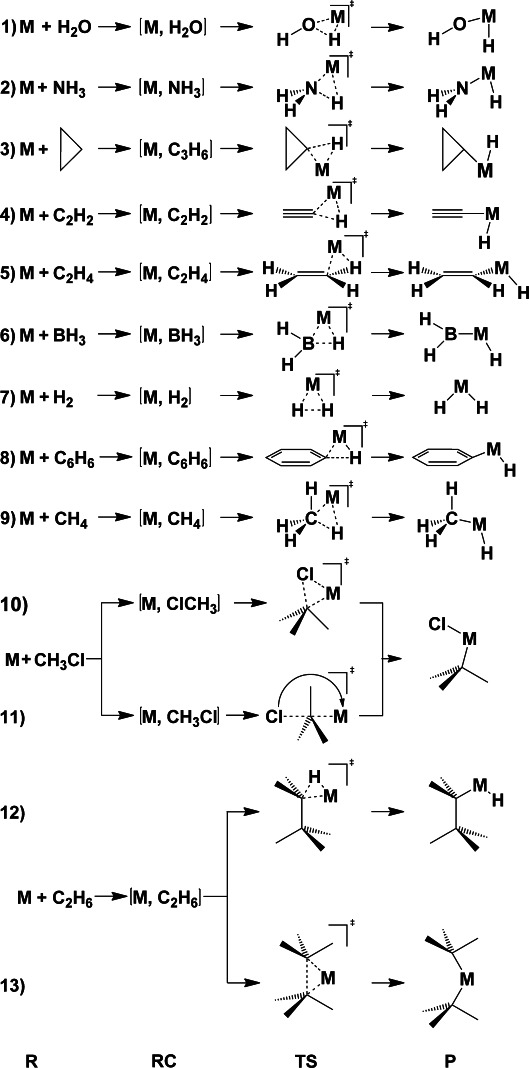
Selected prototype reactions for the test set. ′**M**′ stands for the different catalysts (Pd, PdCl^−^, PdCl_2_, Ni); **R**, reactants; **RC**, complex; **TS**, transition state; **P**, product.

**Figure 2:**
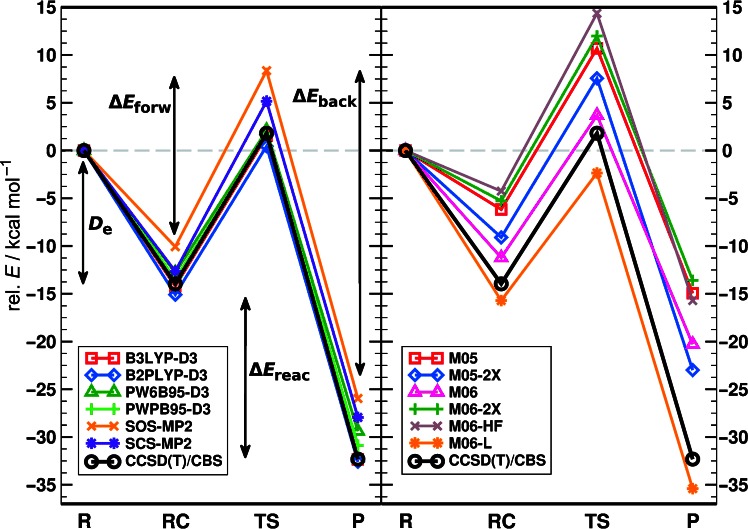
The reaction path for Pd-based activation of the C–Cl bond in chloromethane with selected methods indicated.

The investigated systems are shown in Figure 1, and the reaction proceeds as follows. First, the reactants (**R**) form a complex (**RC**), which then must pass a transition state (**TS**) leading to the metal-bond-inserted product (**P**). Generally the reactions with different catalysts and different bonds follow a similar path on the potential energy surface (PES). For the statistical evaluation of the performance of a method, the dissociation energy of the reactant complex (difference of complex and free reactants, *D*_e_), the forward and backward barrier (difference between the transition state and either the reactant complex (Δ*E*_forw_) or the product (Δ*E*_back_)) and the reaction energy (difference of the product and the reactant complex, Δ*E*_reac_) were taken into account (see Figure 2 for an illustration). The deviation of the reference value is calculated by the difference of the investigated method and CCSD(T)/CBS. Therefore, a positive value stands for an overestimation of the reference value and a negative one for an underestimation.

Only in the case of the palladium atom, all reactions were considered. For the other catalysts it was not always possible to locate a reasonable transition state. Another reason for incompleteness is a failed reference CCSD(T) calculation due to an overly complicated electronic structure for a single-reference method, for example H_2_O with Ni, so that no reasonable reference value would be available. Therefore, several reactions are missing in the subsets. In the Ni set, reactions **1**, **4**, **10**, and **11** are missing, which holds for reactions **6**, **7**, and **11** for the PdCl^−^ case as well. For PdCl_2_ there are no data for reactions **2**, **4**, **6**, and **11**.

All in all, the benchmark contains 164 energy values for comparison, and 205 single-point energies must be computed. In the following we refer to all 164 energies as the “complete set”. If only reactions containing one single catalyst are being considered, this is called a “subset”.

## Computational Details

Except for the Minnesota functionals (denoted as M0X, computed with the NWChem 6.0 program^70^), mPW2PLYP (calculated with ORCA 2.9^71^), and CCSD(T)^72^ (carried out with MOLPRO2009^73^), all calculations and optimizations were done with a modified version of TURBOMOLE 5.7.^74^

The geometries were optimized at the BP86-D2/def2-TZVP level^75^–^78^ for the Ni, Pd, and PdCl_2_ systems, and at the B3LYP-D2/def2-TZVP level^52^, ^53^, ^77^, ^78^ for the PdCl^−^ set, first with constraints to localize the extrema on the PES and afterward freely. Because the reference and DFT values are obtained using the exact same geometries, this choice (which was partially triggered by the previous studies) does not significantly affect the results.

For the single-point DFT calculations the def2-QZVPP basis set^78^ was used, and the small-core ECP ECP28 MWB^79^ for Pd was applied. Several DFs from each functional class were tested. The LDA functional S-VWN^80^, ^81^ was evaluated, but the results are only shown in the Supporting Information. From the General Gradient Approximation (GGA) level, the BP86,^75^, ^76^ PBE,^82^, ^83^ BLYP,^53^, ^75^ and B97-d^77^ functionals were chosen, whereas for the class of meta-GGAs M06L,^22^, ^84^ TPSS,^85^ and its re-optimized version oTPSS^58^ were taken. In addition, the hybrid functionals BHLYP,^86^ PBE0^49^ and B3LYP,^52^, ^53^ and the meta-hybrids M05,^20^ M05-2X^21^, ^22^ (both only shown in Figures 2 and 8 below; see the Supporting Information for details), M06,^22^, ^23^ M06-2X,^22^, ^23^ M06-HF,^22^, ^24^ PW6B95,^50^ BMK,^87^ and TPSSh^85^ were used. From the fifth rung^88^, ^89^ the DHDFs B2PLYP,^55^ B2GPPLYP,^56^ PWPB95, PTPSS,^16^ DSD-BLYP,^61^ PBE0-2,^63^ 1DH-PBE, 1DH-BLYP,^62^ mPW2PLYP,^28^ and PBE0-DH^64^ were adopted. In the perturbative treatment all electrons were taken into account.

In the TURBOMOLE calculations the large grid m5 (grid 4 for the SCF and grid 5 for the final energy evaluation) was applied for the numerical quadrature of the exchange-correlation energy, except for the reaction of cyclopropane with Ni for PWPB95 as well as for some DHDFs (1DH-BLYP, 1DH-PBE, PTPSS, PBE0-DH, and PBE0-2) in which the largest available (reference) grid was used. In all NWChem calculations, we used the *XFine* grid, as it is known that the M0X functionals are sensitive to the grid size.^90^ In the case of ORCA, the large grid 7 (no final energy evaluation) was used.

As wave-function methods for comparison we chose Hartree–Fock (HF), MP2,^68^ and the spin-component-scaled versions^69^ SCS-MP2^67^ and SOS-MP2.^91^ In the case of (SCS-/ SOS-)MP2, only the valence electrons were correlated.

Except for some DHDFs (1DH-BLYP, 1DH-PBE, PTPSS, PBE0-DH, and PBE0-2), in most of the TURBOMOLE calculations the RI for the Coulomb integrals (RI-J)^92^ and for the (double) hybrids for the exchange integrals (RI-K)^93^ was adopted as well. For the perturbative part of the DHDFs (and the MP2 variants) the RI approximation was used^65^ in all calculations. All auxiliary basis sets were taken from the TURBOMOLE basis set library.^94^ The estimated error of the RI approximation in all variants is completely negligible relative to other sources of error and on the order of 0.1–0.2 kcal mol^−1^ for the considered relative energies.

Additionally, we applied our atom pairwise dispersion correction DFTD-D3 (functionals are denoted with “-D3”)^60^ together with the Becke–Johnson (BJ) damping function.^95^

The estimated CCSD(T)/CBS reference values were obtained by using the cc-pVXZ (X=T,Q) basis sets^96^, ^97^ and subsequent extrapolation to the complete basis set (CBS) limit according to the procedure of Halkier et al.^98^, ^99^ For Pd the cc-pVXZ-PP (X=T,Q) sets and the Stuttgart–Dresden ECP ECP28 MDF were used.^100^ In the Pd calculations the 4s4p electrons and for Ni the 3s3p electrons were included for the correlation energy. In the case of the main-group elements only the valence electrons were correlated. The remaining core electrons were kept frozen.

In the case of Ni some electronic structures have partial multi-reference character, as indicated by increased values of the *T*_1_ diagnostic (e.g., 0.037 (**RC** for BH_3_) to 0.157 (**RC** for NH_3_)). In such cases application of single-reference methods like CCSD(T) as reference may not be appropriate, and the error of the reference values (which is, in other cases, expected to be <1 kcal mol^−1^) can be larger. These cases are of borderline character concerning the aim and context of this work, but considering the very approximate nature of some of the DFs tested, we nevertheless decided to include them as a kind of worst-case scenario. The good results obtained from the robust hybrid DFs appear to support this decision (see below).

To check for the basis set superposition error (BSSE) mainly of the CCSD(T)/CBS reference values, we conducted a study for the reaction of Pd with methane as an example. The results of the uncorrected and counter-poise (CP)^101^-corrected calculations as well as the percentage BSSE are listed in Table 1.

**Table 1:** Uncorrected and counter-poise-corrected CCSD(T)/CBS results for the reaction of Pd with methane. B3LYP and B2PLYP are included for comparison

	Uncorr [kcal mol^−1^]	CP-corr [kcal mol^−1^]	BSSE [%]
CCSD(T)/CBS			
*D*_e_	9.9	9.3	6.5
Δ*E*_forw_	12.3	12.7	3.3
Δ*E*_back_	4.1	3.8	7.9
Δ*E*_reac_	8.2	8.9	8.5
B2PLYP			
*D*_e_	7.7	6.9	11.6
Δ*E*_forw_	12.0	12.3	2.5
Δ*E*_back_	4.6	4.4	4.5
Δ*E*_reac_	7.4	7.9	6.8
B3LYP			
*D*_e_	5.0	4.8	4.2
Δ*E*_forw_	12.5	12.5	0.0
Δ*E*_back_	6.2	6.1	1.6
Δ*E*_reac_	6.3	6.4	1.6

As shown in Table 1 the remaining BSSE is between 0.3 and 0.7 kcal mol^−1^ corresponding to 3–8.5 % of the reaction energy in the CCSD(T)/CBS treatment. This is acceptable considering the average errors from DFs which are typically an order of magnitude larger. Due to the fact that the perturbative part in DHDFs is not fully converged with the largest def2-QZVPP basis set, a similar BSSE is noted in Table 1 for the example of B2PLYP. Hybrid DFs exhibit small BSSEs <0.2 kcal mol^−1^ with such large basis sets as demonstrated by the B3LYP values.

## Results and Discussion

### Statistical data

**Figure 3:**
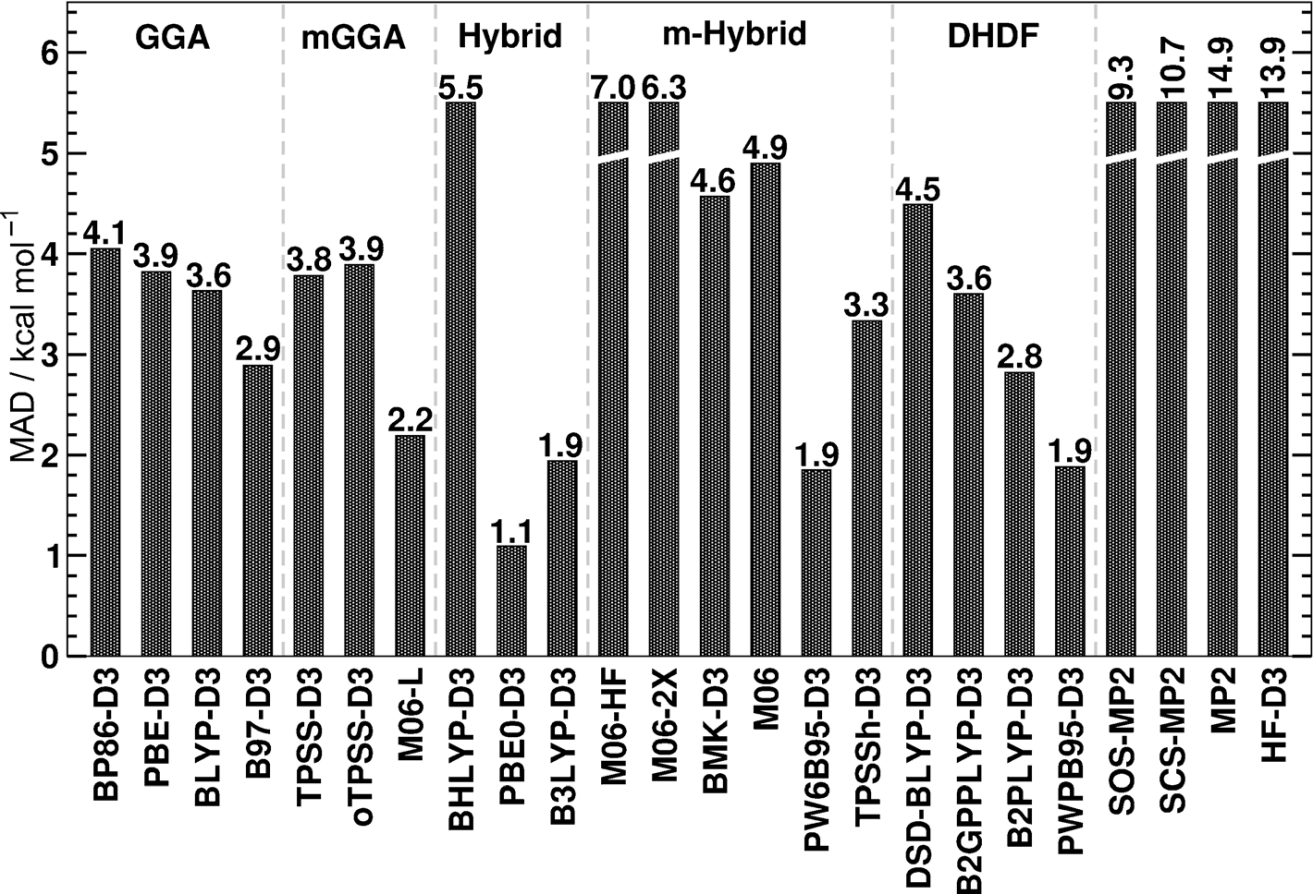
MAD over the complete set compared with CCSD(T)/CBS. The functionals are ordered according to Jacob’s ladder and Fock exchange. The bars are capped if the MAD is >5.5 kcal mol^−1^.

The results for the complete set are presented in Figure 3 in a highly condensed form as mean absolute deviation (MAD) from the reference values, and more details are given in the Supporting Information. Over all 164 points in the test set, the best functional is PBE0-D3 with an MAD of 1.1 kcal mol^−1^, followed by PW6B95-D3, the corresponding double hybrid PWPB95-D3, and B3LYP-D3 (each 1.9 kcal mol^−1^). These results are in line with the findings of Quintal et al.^47^

The performances of the GGAs BP86-D3, PBE-D3, and BLYP-D3 are similar, with respective MADs of 4.1, 3.9, and 3.6 kcal mol^−1^. B97-D3 is outstanding here with a value of 2.9 kcal mol^−1^, which is closer to those of the hybrids. M06L performs surprisingly well, with an MAD of 2.2 kcal mol^−1^. The other meta-GGAs TPSS and a re-optimized version, namely oTPSS, have similar MADs to those of most of the other GGAs (3.8 and 3.9 kcal mol^−1^, respectively).

Compared with the other hybrid functionals BHLYP-D3 performs much worse. The main problem is the description of the van der Waals (vdW)-type reactant complex, which is much too high in energy (up to 10 kcal mol^−1^ in some cases, see Supporting Information). The hybrids are ordered with respect to the amount of Fock exchange included, from which an optimum of ∼25 % as in PBE0 can be deduced.

In the class of the meta-hybrid functionals, the M0X approaches partially fail. The problem is related to the Fock exchange contribution as for the other hybrids. This is apparent by the corresponding MADs, which drop from 7.0 kcal mol^−1^ (M06-HF, 100 % Fock exchange), over 6.3 kcal mol^−1^ (M06-2X, 54 % Fock exchange) to 4.9 kcal mol^−1^ (M06, 27 % Fock exchange). A similar behavior as with M06 is obtained for BMK-D3, with an MAD of 4.6 kcal mol^−1^. This result is in line with the findings of Quintal et al.^48^ A partial failure of the ‘high-Fock-exchange’ M06 functionals for transition-metal systems had already been noted by the developers of these methods, and is therefore not unexpected. The best meta-hybrid is PW6B95-D3 (1.9 kcal mol^−1^), which has a similar admixture of Fock exchange (28 %) as PBE0-D3.

In the group of double hybrids, PWPB95-D3 shows the best performance with an MAD of 1.9 kcal mol^−1^, and it is second best of all tested methods, followed by B2PLYP-D3 (2.8 kcal mol^−1^), the re-parameterized version B2GPPLYP-D3 (3.6 kcal mol^−1^), and DSD-BLYP (4.5 kcal mol^−1^). Notably, the DHDF with the highest amount of Fock exchange performs worst, and the functional with the lowest, best. Another interesting observation is made by comparing B2PLYP-D3 and PWPB95-D3, as both functionals have a similar amount of Fock exchange with 53 % and 50 %, respectively. This also holds for the perturbative part with 27 % and 26.9 %, respectively. However, the behavior of PWPB95-D3 is significantly (almost 1 kcal mol^−1^) better than B2PLYP-D3. The reason is that the perturbative part in PWPB95 contains only the opposite-spin (OS) component of the second-order correlation energy. In Figure 3 different variants of MP2 are also shown, and a comparison of the performance of MP2 (full OS part, full same-spin (SS) contribution) and SOS-MP2 (no SS part) underlines the unfavorable effect of the SS correlation if evaluated perturbatively (MAD(SOS-MP2)=9.3 kcal mol^−1^, MAD(MP2)=14.9 kcal mol^−1^). This also holds true for DSD-BLYP, as it uses SCS-MP2-type correlation (46 % OS, 37 % SS) instead of normal MP2. However, the main issue of this functional is the high Fock exchange admixture (69 %). The wave-function-based methods overall show poorer behavior than DFT average performers. One reason for this is the nickel subset, which is very demanding for single-reference wave-function methods such as MP2. However, this is explained in greater detail below. Hartree–Fock, together with the D3 dispersion correction, performs slightly better (MAD=13.9 kcal mol^−1^) than MP2. The effect of the D3 correction is discussed separately.

**Figure 4:**
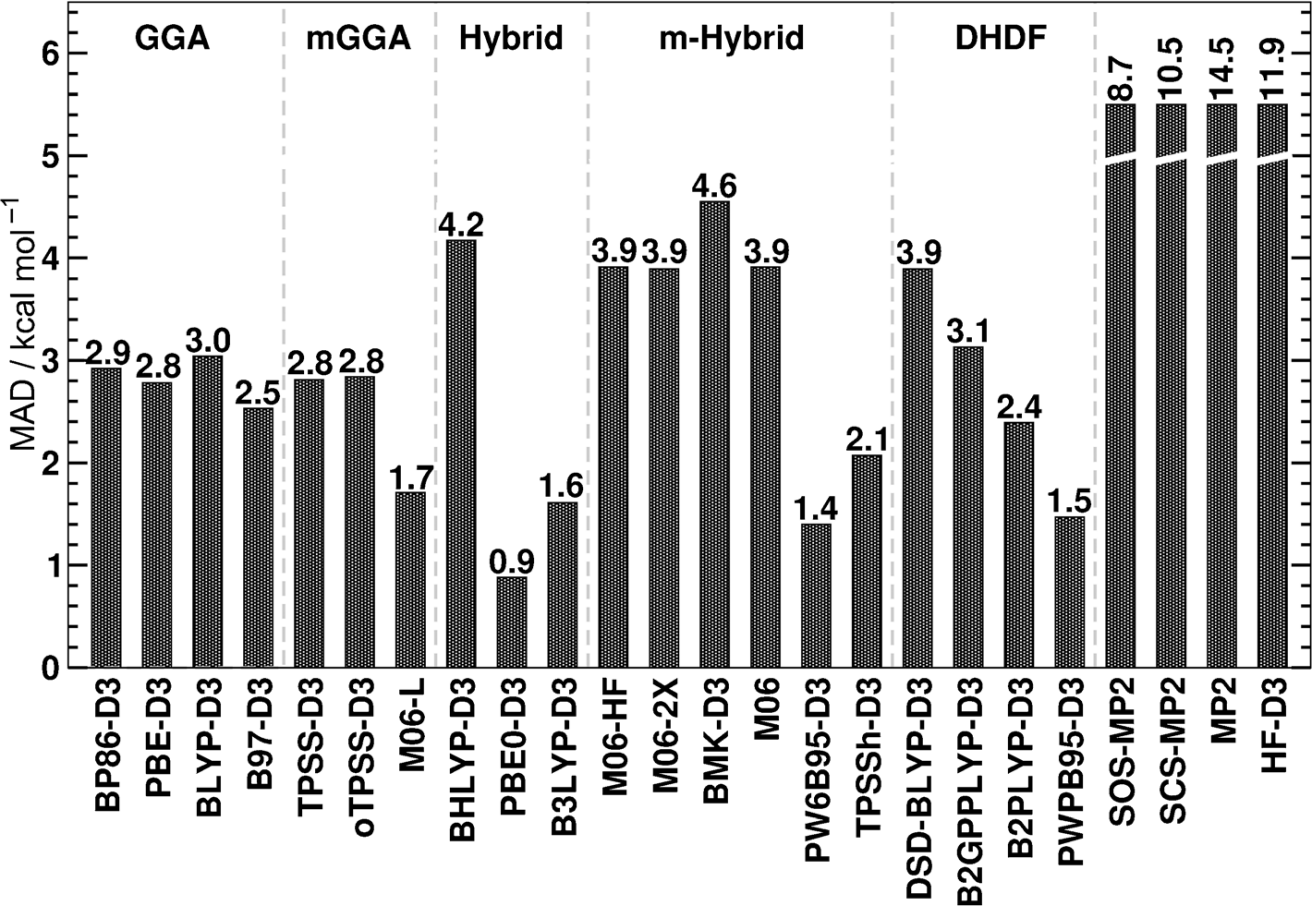
MAD over the complete set without the dissociation and reaction energy compared with CCSD(T)/CBS. The functionals are ordered according to Jacob’s ladder and Fock exchange. The bars are capped if the MAD is >5.5 kcal mol^−1^.

**Figure 5:**
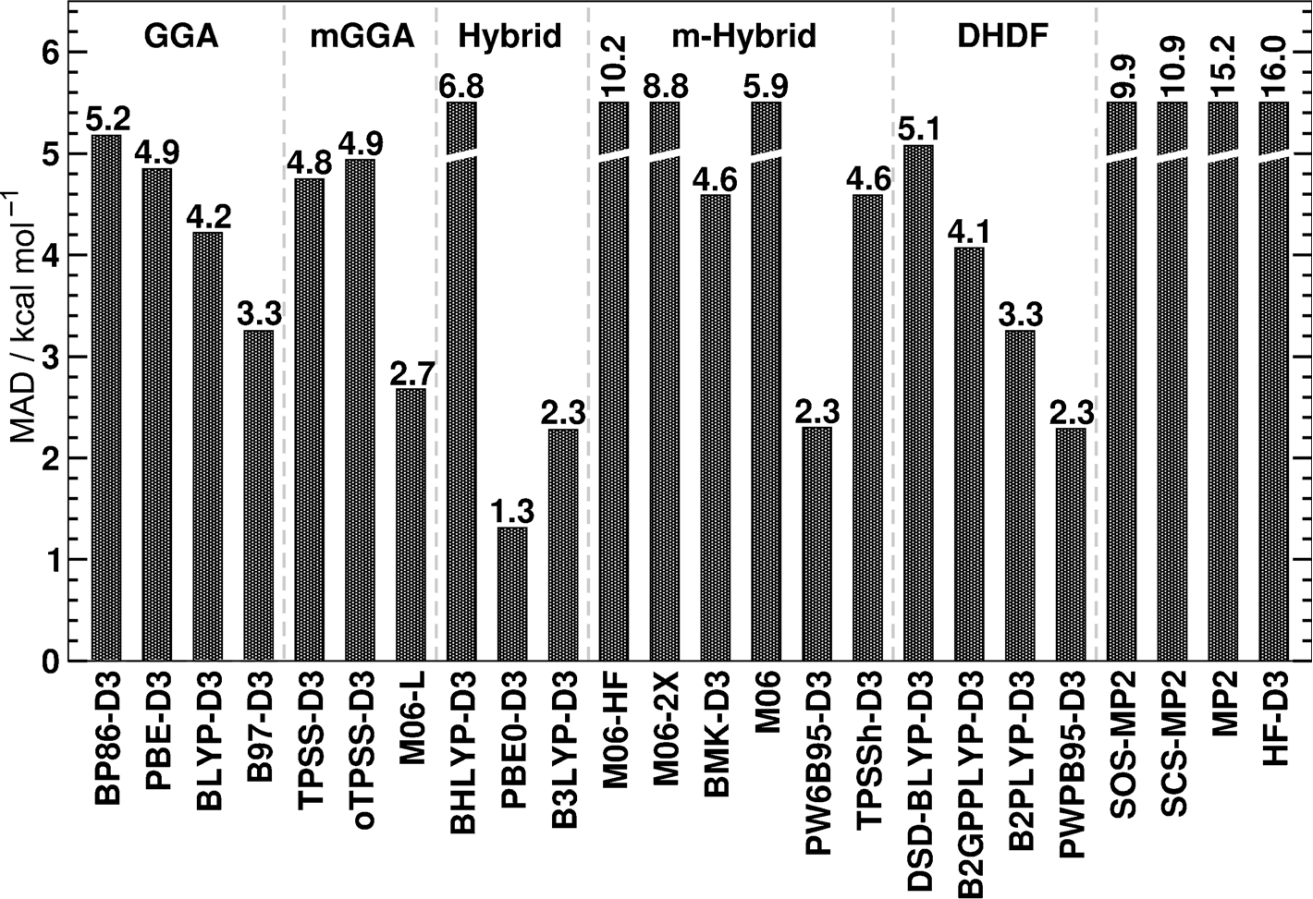
MAD over the complete set without the barriers compared with CCSD(T)/CBS. The functionals are ordered according to Jacob’s ladder and Fock exchange. The bars are capped if the MAD is >5.5 kcal mol^−1^.

Figures 4 and 5 are similar to Figure 3, but they contain only the barriers or only the reaction energies, respectively. Based on these results it can be concluded that the main problem for most of the tested DFs is the description of the latter. Thereby, the largest difference between the MADs of the thermochemistry and the barriers (ΔMAD^thermo–kin^) appears for the M06 functionals which additionally depends on the Fock exchange admixture (ΔMAD^thermo–kin^ between 2.0 kcal mol^−1^ for M06 and 6.3 kcal mol^−1^ for M06-HF). Functionals that are not strongly affected are, for example, PBE0-D3 with a difference of only 0.4 kcal mol^−1^. For the tested perturbation methods, a small ΔMAD^thermo–kin^ value is observed for SOS-MP2 and SCS-MP2 (1.2 and 0.4 kcal mol^−1^, respectively), whereas a greater difference is noted for HF-D3 (4.1 kcal mol^−1^).

**Figure 6:**
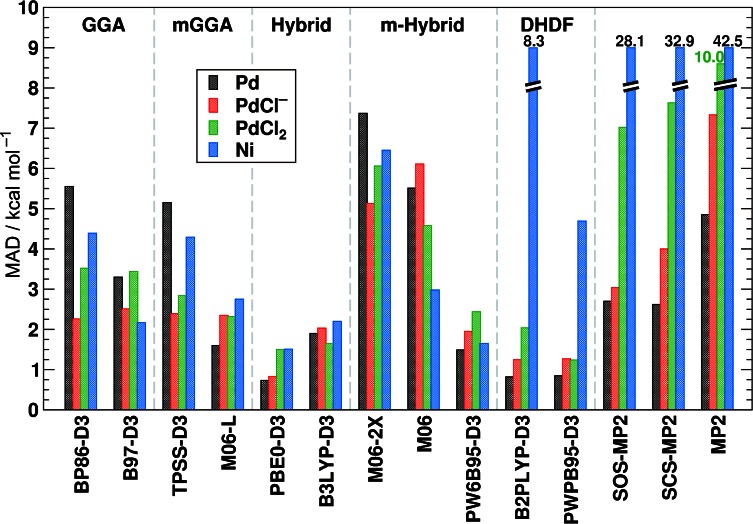
MAD for each subset for selected methods with respect to CCSD(T)/CBS. The functionals are ordered according to Jacob’s ladder and Fock exchange. The bars are capped if the MAD is >9 kcal mol^−1^.

A more detailed picture is shown in Figure 6, in which the performance of selected methods for each subset is presented. The best DF is PBE0-D3, which in two out of the four subsets shows MADs <1 kcal mol^−1^ (which is typically considered as ‘chemical accuracy’). From the other functionals, only DHDFs yield an MAD of <1 kcal mol^−1^ for the palladium test set.

Furthermore, a few of the tested methods perform rather differently for each subset, which is discussed using ΔMAD^w–b^ values, that is, the difference between the worst and the best MADs. One extreme example is BP86-D3, which has an ΔMAD^w–b^ value of 3.3 kcal mol^−1^ between the best subset (MAD (PdCl^−^)=2.3 kcal mol^−1^) and the worst (MAD(Pd)=5.6 kcal mol^−1^). Another functional with a large difference is M06, which has an ΔMAD^w–b^ value of 3.1 kcal mol^−1^. This difference in performance is observed for the double hybrids as well. The reason here are the results for the nickel subset. The double hybrids yield bad results here because these functionals are more sensitive to cases with difficult electronic structures due to the use of the perturbative correlation. The results for the MP2 variants support this view. The worst results are provided by MP2, which has large MADs for each of the subsets: MAD(Pd)=4.9 kcal mol^−1^, MAD(PdCl^−^)=7.3 kcal mol^−1^, MAD(PdCl_2_)=10.0 kcal mol^−1^, MAD(Ni)=42.5 kcal mol^−1^. The scaled variants SCS-MP2 and SOS-MP2 perform much better, and for the nickel subset the MAD is decreased to 32.9 and 28.1 kcal mol^−1^, respectively. Improvements are also noted for the PdCl_2_ subset (7.6 and 7.0 kcal mol^−1^, respectively).

Based on these results the different behavior of the DHDFs, especially for the nickel subset, can be rationalized. PWPB95 has the lowest MAD for this case (4.7 kcal mol^−1^), as it incorporates only a small part of SOS-MP2 (26.9 %). B2PLYP includes a similar amount of perturbative correlation (27 %) but contains normal MP2-type correlation, and the MAD increases to 8.3 kcal mol^−1^. The re-parameterized version B2GPPLYP contains 36 % MP2 correlation, resulting in a further increased MAD of 11.2 kcal mol^−1^.

On the other hand, there are DFs that perform similarly for each subset, indicating greater robustness. The best performer is B3LYP-D3, with a ΔMAD^w–b^ value of 0.5 kcal mol^−1^ followed by PBE0-D3 and PW6B95-D3, with respective ΔMAD^w–b^ values of 0.8 and 0.9 kcal mol^−1^.

### Effect of the dispersion correction

**Figure 7:**
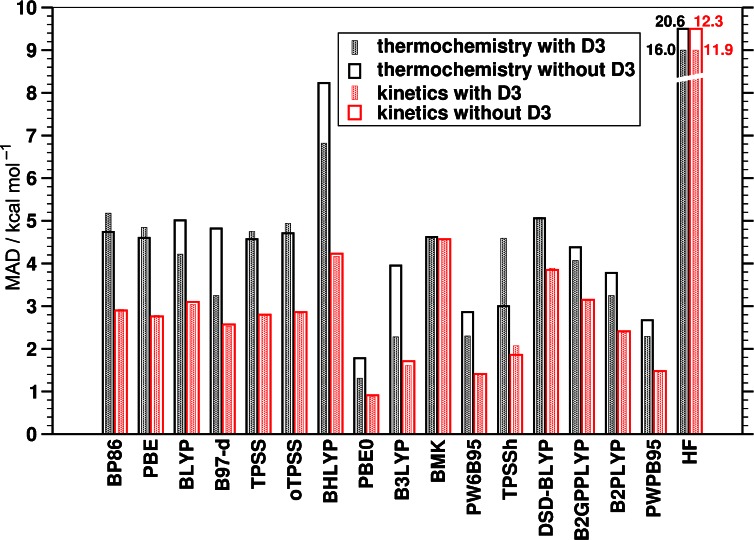
MAD for the thermochemistry (i.e., *D*_e_, Δ*E*_reac_) and kinetics (i.e., Δ*E*_forw_, Δ*E*_back_) of the complete set, with and without dispersion correction, with respect to CCSD(T)/CBS. The functionals are ordered according to Jacob’s ladder, and the bars are capped if the MAD is >9 kcal mol^−1^.

In this section we investigate the influence of the dispersion correction on the MADs for the barriers and the reaction energies separately. The results are shown in Figure 7. The Minnesota functionals are excluded because they might require dispersion corrections only for very large complexes. For the discussion we use the absolute value of the difference between the MAD of the dispersion corrected and the uncorrected functional (ΔMAD^|D3−noD3|^).

In their investigations, Lai et al.^51^ reported that the dispersion correction has no influence on the forward barriers. This is corroborated in our investigation for the backward barriers as well. A significant effect is only discovered for TPSSh (ΔMAD^|D3−noD3|^=0.2 kcal mol^−1^) and HF (ΔMAD^|D3−noD3|^= 0.4 kcal mol^−1^).

However, we obtained large effects on the reaction energies in some cases. The biggest change in MAD is found for HF (ΔMAD^|D3−noD3|^=4.6 kcal mol^−1^). This is understandable because in HF the Coulomb correlation is entirely missing, which is partly included in the medium-range part of the D3 correction. For the DFs the biggest influence is found for B97-d (ΔMAD^|D3−noD3|^=1.5 kcal mol^−1^), BHLYP (ΔMAD^|D3−noD3|^=1.4 kcal mol^−1^), B3LYP (ΔMAD^|D3−noD3|^=1.7 kcal mol^−1^), and TPSSh (ΔMAD^|D3−noD3|^=1.6 kcal mol^−1^). As can be observed from Figure 7, in most cases the MAD is lowered by adding a dispersion correction.

### Example reactions

In this section we discuss two reactions as examples in greater detail. The first is the oxidative addition of Pd into the C–Cl bond of chloromethane shown in Figure 2. Based on the CCSD(T) results, the reactants form a complex (**RC**) with a stabilization energy of 14.0 kcal mol^−1^. Then the Pd atom inserts into the C–Cl bond via a transition state (**TS**) which is 1.8 kcal mol^−1^ higher in energy than the free reactants. The product (**P**) is formed directly from the **TS** and is 32.3 kcal mol^−1^ more stable than the reactants.

Several methods are included in Figure 2 for comparison. For a clearer picture the results are split into two plots. On the left side two hybrid functionals (namely B3LYP-D3 and PW6B95-D3), two double-hybrid functionals (B2PLYP-D3 and PWPB95-D3), and two wave-function-based methods (SCS-MP2 and SOS-MP2) are presented. The plots suggest that the DFs give a qualitatively and quantitatively balanced representation of the CCSD(T)/CBS energy surface, and only PW6B95-D3 has some problems in the description of the backward barrier (Δ*E*_back_). The two wave-function methods also seem to have problems with this reaction. SCS-MP2 overestimates the forward barrier, and SOS-MP2 yields a reactant complex which is too high in energy. However, the barriers are qualitatively correct.

In the right-hand part of Figure 2, the results of all tested Minnesota functionals are plotted. The only functional that qualitatively reproduces the reference values is the meta-GGA M06L, although we note that Δ*E*_forw_ is too small. For the M06 series the reactant complex is increasingly destabilized with increasing admixture of Fock exchange. Also notable, for M06 both barriers are underestimated, but with increasing amount of Fock exchange this turns into an overestimation. This picture for M06, M06-2X, and M06-HF is complicated and cannot be rationalized easily. Overall, the Minnesota functionals perform worse than many standard DFs and also less well than one might have expected.

**Figure 8:**
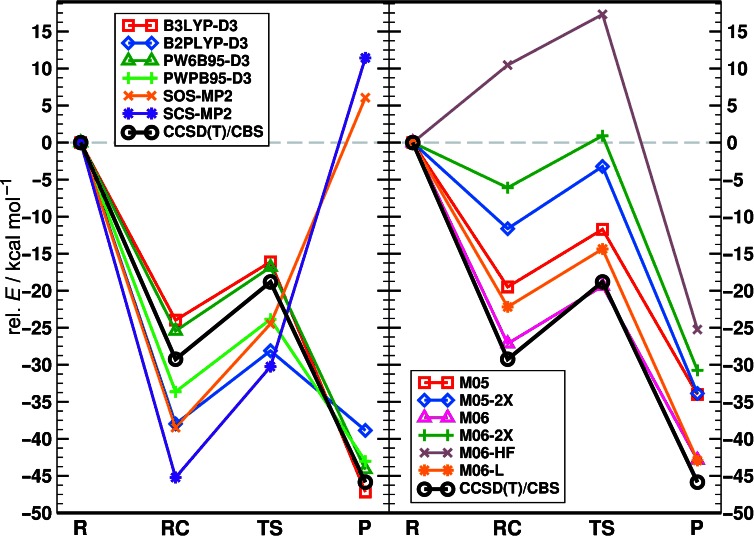
The reaction path of Ni-based activation of the C–C bond in ethane with selected methods indicated.

The second example is the oxidative insertion of Ni into the C–C bond of ethane (Figure 8). The CCSD(T)/CBS values indicate strongly exothermic complex formation (**RC**), which is 29.3 kcal mol^−1^ lower in energy than the free reactants. The forward barrier leading is 10.4 kcal mol^−1^, and the product is 45.9 kcal mol^−1^ lower in energy than the reactants.

The same methods as in Figure 2 are presented in Figure 8. The data reveal that only the hybrid functionals are able to reproduce the CCSD(T)/CBS values quantitatively. However, a small underestimation of the reactant complex and the forward barrier is observed. Additionally, in the case of B3LYP-D3 the value of Δ*E*_back_ is slightly too large.

The DHDFs yield qualitatively correct results. Thereby, PWPB95-D3 performs much better than B2PLYP-D3. However, in both cases *D*_e_ and Δ*E*_back_ are too small, and in fact for B2PLYP-D3 Δ*E*_reac_ is nearly zero. The reason is the poor behavior of the perturbative part as explained above. SCS-MP2 and SOS-MP2 show similar errors. The forward barrier is reasonable, while the **RC** energy is strongly overestimated, and the backward barrier is negative.

On the right side of Figure 8, the results for the Minnesota functionals are shown. They mostly give a qualitatively correct picture for this reaction, with M06 performing best. However, both barriers are underestimated. The meta-GGA M06L is the second-best functional, although it clearly underestimates the reactant complex and overshoots the backward barrier. By increasing the amount of Fock exchange, a similar behavior is observed for the M06 series as for the first example (Figure 2).

### Performance of double-hybrid functionals

In the last section we present the results of a detailed evaluation of newly developed DHDFs compared with well-known variants. The dispersion correction is neglected in all cases for consistency. The methods in Table 2 are ordered according to the Fock exchange admixture for analysis purposes. The most extreme variants are PBE0-2, with 79.37 % of Fock exchange and 50 % of MP2 correlation, and PBE0-DH (50 % of Fock exchange and 12.5 % of MP2 correlation). Additionally, spin-component-scaled parts were used in three functionals, namely DSD-BLYP (using an SCS-MP2 variant), PTPSS, and PWPB95 (both using SOS-MP2).

**Table 2:** All tested DHDFs ordered with respect to the amount of Fock exchange (FE)

Functional	FE [%]	MP2 corr [%]	Functional	FE [%]	MP2 corr [%]
PBE0-2	79.37	50	mPW2PLYP	55	25
DSD-BLYP	69	37 SS, 46 OS	B2PLYP	53	27
1DH-PBE^[a]^	67.5	45.5625	PTPSS	50	37.5 OS
1DH-BLYP	65	42.25	PWPB95	50	26.9 OS
B2GPPLYP	65	36	PBE0-DH	50	12.5

[a] Unpublished parameters, similar to 1DH-BLYP.^62^

**Figure 9:**
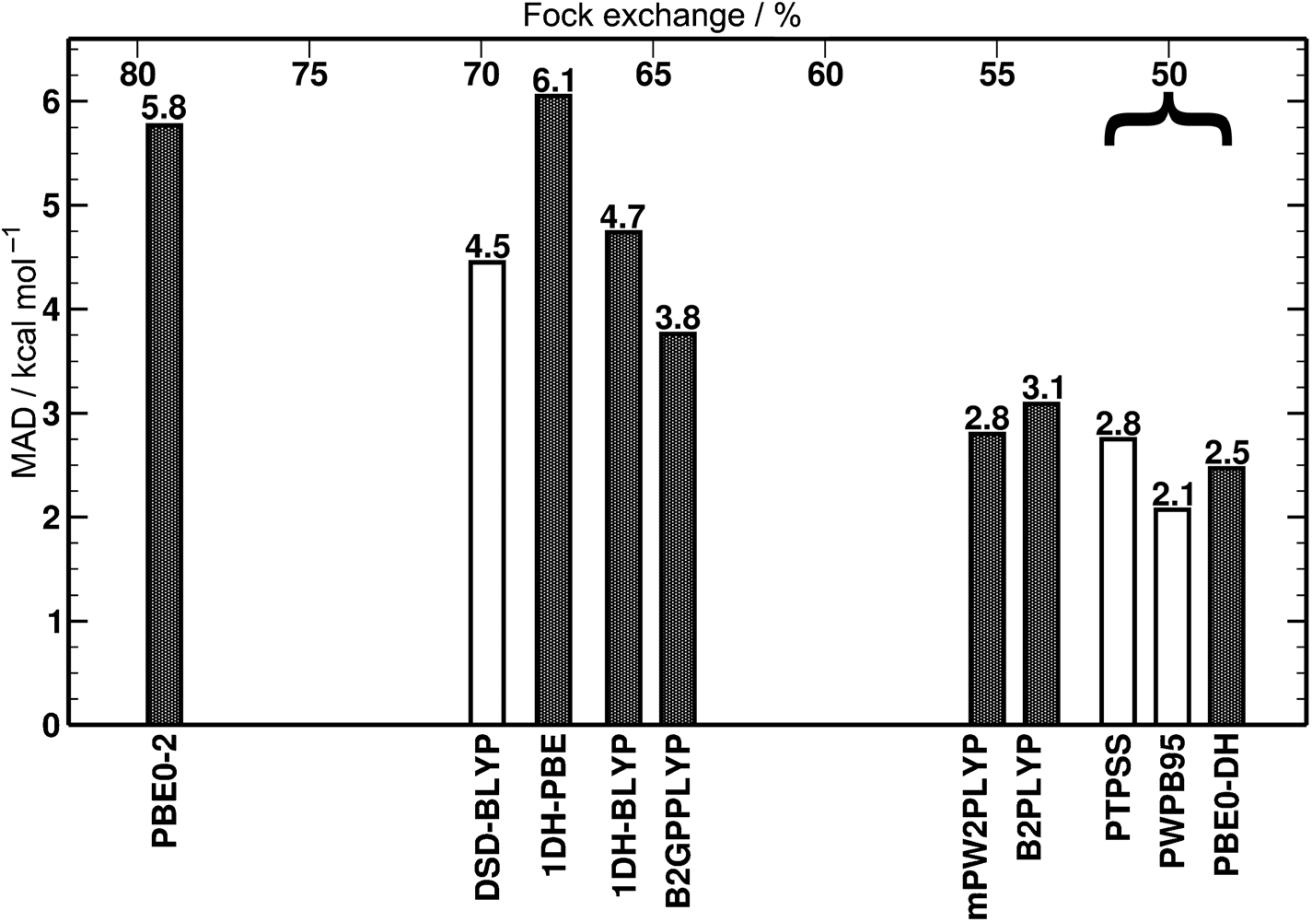
MAD over the complete set for all DHDFs compared with CCSD(T)/CBS. The functionals are ordered according to the amount of Fock exchange included. DHDFs with SCS-type MP2 correlation are shown as open bars.

The results of the tested DHDFs for the complete set are presented in Figure 9. With an MAD of 2.1 kcal mol^−1^, PWPB95 is the best DHDF followed by PBE0-DH (2.5 kcal mol^−1^), PTPSS, and mPW2PLYP (2.9 kcal mol^−1^ each). B2PLYP is slightly worse, with an MAD of 3.1 kcal mol^−1^. Because the DHDFs are ordered with respect to the amount of Fock exchange, Figure 9 shows that DHDFs with the lowest amount perform best (50–55 %). With increasing amounts of Fock exchange, the MAD increases. The largest MADs are obtained for 1DH-PBE (6.1 kcal mol^−1^) and PBE0-2 (5.8 kcal mol^−1^).

However, the perturbative part also plays an important role. A large contribution of MP2 correlation is problematic in electronically complicated cases like the nickel subset (see Figure 6) as already noted. This is supported by the data in Figure 9. Additionally, DHDFs with spin-component-scaled correlation were tested as well, namely DSD-BLYP (37 % SS, 46 % OS), PTPSS, and PWPB95 (both using the opposite-spin part only (PTPSS: 37.5 %, PWPB95: 26.9 %)). Compared with other double-hybrid functionals with very high amounts of Fock exchange (>65 %) DSD-BLYP performs better, with an MAD of 4.5 kcal mol^−1^ due to the incorporation of SCS-MP2-type correlation. Only B2GPPLYP has a lower MAD, but this is related to the lower amount of perturbative correlation relative to DSD-BLYP. PWPB95 shows the best performance for all tested double hybrids and seems to be more robust for electronically complicated cases due to the use of the OS term.

## Conclusions

This work presents results of an extensive benchmark study of 23 density functionals and wave-function methods for prototype bond activations with four different model catalysts, namely Pd, PdCl^−^, PdCl_2_, and Ni. The estimated CCSD(T)/CBS energies based on DFT-optimized geometries served as reference. In general, extended Gaussian AO basis sets were used which provide results quite close to the CBS (typically better than 0.5 kcal mol^−1^ for relative energies). Basis set superposition errors at a quadruple-ζ basis set level appear to be insignificant.

The PBE0 hybrid functional, together with our atom-pairwise dispersion correction (D3), shows the best performance for the complete set followed by PW6B95-D3, the corresponding double-hybrid functional PWPB95-D3, and B3LYP-D3. Moreover, we could reproduce the findings of Quintal et al.^48^ for a subset of our systems and were able to support their statement that there is no truly outstanding functional, but several functionals with a small (20–30 %) amount of Fock exchange which perform well. Functionals with meta-GGA correlation, except for PW6B95-based functionals and M06L, seem to offer no advantage. The functionals of the M06 series with large amounts of Fock exchange perform poorly. ‘Cheap’ (meta-)GGAs perform often worse than hybrid functionals, but some (such as B97-D3 and M06L) offer a good compromise between cost and accuracy and can be recommended for exploratory investigations.

Compared with the density functionals, the (SOS-/SCS-)MP2 methods generally perform worse mainly because of the bad results for the electronically complicated systems with PdCl_2_ and Ni in particular. From these methods only SOS-MP2 or SCS-MP2 can be recommended for comparative investigations.

We also systematically investigated the influence of the D3 dispersion correction for all reactions. In general, the barriers are not affected by adding this medium-range correlation term, which is in line with results reported by Lai et al.^51^ However, for the reaction energies we found an occasional significant influence. The biggest effect is observed for B3LYP and B97-d, for which the MAD decreases by 1.7 and 1.5 kcal mol^−1^, respectively.

For the first time we investigated ten (partially very recently proposed) double-hybrid functionals on a realistic transition-metal benchmark set with a particular eye on the influence of the amount of Fock exchange and the different variants of the perturbative correlation. This study reveals that DHDFs with 50–60 % of Fock exchange and <30 % of perturbative correlation perform best. The PWPB95 method that incorporates only the opposite-spin part is found to be slightly better than the other functionals. These results are in line with the findings for the MP2-type methods for which inclusion of same-spin correlation leads to a deterioration of accuracy.

Based on all results the best ‘cost/performance’ ratio is offered by hybrid functionals with small amounts of Fock exchange. If electronically very complicated systems are not of interest (i.e., if one excludes the Ni subset from the benchmark), the double hybrid PWPB95-D3 is similar in accuracy to the best hybrid (which is PBE0-D3) and can therefore be recommended for well-behaved transition-metal systems as well. We also point out that the D3 dispersion correction always has a positive (or small) effect on the results and is therefore recommended as a default in DFT computations of organometallic thermochemistry similar to main-group systems.

## Supplementary material

As a service to our authors and readers, this journal provides supporting information supplied by the authors. Such materials are peer reviewed and may be re-organized for online delivery, but are not copy-edited or typeset. Technical support issues arising from supporting information (other than missing files) should be addressed to the authors.
