# Supplementary material for: Benchmark Study of the Performance of Density Functional Theory for Bond Activations with (Ni,Pd)-Based Transition-Metal Catalysts
Source: ChemistryOpen. 2013 Jun 3;2(3):115–24. doi: 10.1002/open.201300012 (PMC3703816; doi:10.1002/open.201300012)
Supplement: Supplementary file 1 [file open0002-0115-SD1.pdf]

ChemistryOPEN

Supporting Information

© 2013 The Authors. Published by Wiley-VCH Verlag GmbH & Co. KGaA, Weinheim

Benchmark Study of the Performance of Density Functional Theory for Bond Activations with (Ni,Pd)-Based Transition-Metal Catalysts

Marc Steinmetz and Stefan Grimme*[a]

open_201300012_sm_miscellaneous_information.pdf

Contents

1 Coordinates in the TURBOMOLE Format 2
1.1 Pd subset . . . 2
1.2 $PdCl^-$ subset . . . 12
1.3 $PdCl_2$ . . . 20
1.4 Ni subset . . . 28

2 Complete Results for all Subsets 36
2.1 Pd subset . . . 36
2.2 $PdCl^-$ subset . . . 54
2.3 $PdCl_2$ subset . . . 69
2.4 Ni subset . . . 83

1 Coordinates in the TURBOMOLE Format

1.1 Pd subset

Reaction **1**:

reactant

```
$coord
    1.45073772696982      0.00000000000000      0.37481785522416      h
    0.00000000000000      0.00000000000000     -0.74963571044831      o
   -1.45073772696982      0.00000000000000      0.37481785522416      h
$end
```

reactant complex

```
$coord
   -1.31039770580726     -0.33626451386260     -1.46832059559111      h
    3.36590658278668      0.04857449520212      0.00000000000000      pd
   -0.74511117117215      0.62395453252309      0.00000000000000      o
   -1.31039770580726     -0.33626451386260      1.46832059559111      h
$end
```

transition state

```
$coord
   -0.79249000000000     -1.46633900000000     -0.42725550000000      h
    2.14196900000000     -1.46633900000000     -0.42725550000000      pd
   -0.36352000000000      1.30042600000000     -0.42725550000000      o
   -0.98595900000000      1.63225200000000      1.28176650000000      h
$end
```

product

```
$coord
   -1.73496440122395     -2.17944154836249      0.68187556165830      h
    0.99788785710462     -1.89237287409845     -0.11158697359067      pd
    0.99650108715957      1.69455448087782      0.30130089002408      o
   -0.25942454304025      2.37725994158313     -0.87158947809171      h
$end
```

Reaction **2**:

reactant

```
$coord
    0.00000000000000     -0.54747998206517      0.00000000000000      n
    0.89444426225340      0.18249332735506      1.54922290676135      h
    0.89444426225340      0.18249332735506     -1.54922290676135      h
   -1.78888852450680      0.18249332735506      0.00000000000000      h
$end
```

reactant complex

```
$coord
    0.00000000000000     -0.37525886322984      0.00000000000000      n
    0.00000000000000      3.52543401388399      0.00000000000000      pd
   -0.90713312732291     -1.05005838355138     -1.57120066575213      h
   -0.90713312732291     -1.05005838355138      1.57120066575213      h
    1.81426625464582     -1.05005838355138      0.00000000000000      h
$end
```

transition state

```
$coord
   -0.76051540077399      0.72737768400824      0.00000000000000      n
    2.99872959897827      0.72737768400824      0.00000000000000      pd
   -1.68124340095766      0.06586766314423      1.56794255230948      h
    1.12427260371103     -1.58649069430494      0.00000000000000      h
   -1.68124340095766      0.06586766314423     -1.56794255230948      h
$end
```

product

```
$coord
    1.26329572611985      0.95290406093672      0.00000000000000      n
    1.91634485047249     -0.03755994803539     -1.54464860222936      h
    1.91634485047249     -0.03755994803539      1.54464860222936      h
   -2.37468315215401      0.99291292604882      0.00000000000000      pd
   -2.72130227491083     -1.87069709091477      0.00000000000000      h
$end
```

Reaction **3**:

reactant

```
$coord
    0.00000000000000      1.42934135450848     -0.82523061578934      c
    0.00000000000000     -1.42934135450848     -0.82523061578934      c
    0.00000000000000      0.00000000000000      1.65046123157867      c
   -1.73354298999174      2.39619067133508     -1.38344132912498      h
    1.73354298999174      2.39619067133508     -1.38344132912498      h
   -1.73354298999174     -2.39619067133508     -1.38344132912498      h
    1.73354298999174     -2.39619067133508     -1.38344132912498      h
    1.73354298999174      0.00000000000000      2.76688265824996      h
   -1.73354298999174      0.00000000000000      2.76688265824996      h
$end
```

reactant complex

```
$coord
   -0.55989810960346      1.27401993525355     -1.43728953846627      c
   -0.55989810960346      1.27401993525355      1.43728953846627      c
    0.40865278276669     -0.98789545913573      0.00000000000000      c
    0.81356226092327      2.47304536184563     -2.40232115502939      h
   -2.37432721289806      1.10544296542911     -2.40423264876506      h
    0.81356226092327      2.47304536184563      2.40232115502939      h
   -2.37432721289806      1.10544296542911      2.40423264876506      h
   -0.86973454616736     -2.68910720721363      0.00000000000000      h
    2.53660195381584     -1.21626883971567      0.00000000000000      h
    2.16580593274136     -4.81174501899152      0.00000000000000      pd
$end
```

transition state

```
$coord
   -1.48644400000000     -1.27440720000000     -0.31228870000000      c
    1.35411600000000     -1.27440720000000     -0.31228870000000      c
   -0.05549100000000      1.24497780000000     -0.31228870000000      c
   -2.44660900000000     -1.84636420000000      1.41851630000000      h
   -2.48300100000000     -1.77046120000000     -2.05136370000000      h
    2.30702900000000     -1.86498020000000      1.41766530000000      h
    2.33994200000000     -1.78449120000000     -2.05384770000000      h
    0.03186500000000      2.27197980000000     -2.10340970000000      h
    1.58084800000000      2.68732880000000      1.81521930000000      h
   -1.14225500000000      3.61082480000000      2.49408630000000      pd
$end
```

product

```
$coord
   -1.83566263944623     -1.13587828919109      0.20934184183871      c
    1.00133932072027     -1.64552905761681      0.21248127295742      c
    0.02072465920340      0.80986243653376     -0.80801953602856      c
   -2.70166257094125     -0.83268578478355      2.05956910962439      h
   -3.05865298935797     -2.09490397596492     -1.15055540470771      h
    1.91854647281339     -1.65267487710158      2.05806196737699      h
    1.76975726496593     -3.00764997094749     -1.13708525446768      h
    0.10415344495253      1.08585062219356     -2.85622450133042      h
    2.76570213786424      4.47735440222099      0.32460591275298      h
    0.01575489922573      3.99625449465712      1.08782459198389      pd
$end
```

Reaction **4**:

reactant

```
$coord
    0.00000000000000      0.00000000000000     -1.14117422703006      c
    0.00000000000000      0.00000000000000      1.14117422703006      c
    0.00000000000000      0.00000000000000      3.16530882872916      h
    0.00000000000000      0.00000000000000     -3.16530882872916      h
$end
```

reactant complex

```
$coord
    3.04593158923872      0.00000000000000      1.24700588452024      h
    1.19885901053401      0.00000000000000      0.37884542062175      c
   -1.19885901053401      0.00000000000000      0.37884542062175      c
    0.00000000000000      0.00000000000000     -3.25170261028396      pd
   -3.04593158923872      0.00000000000000      1.24700588452024      h
$end
```

transition state

```
$coord
   -0.19545763643666     -0.15401057806297      0.00000000000000      c
    2.09212202686021     -0.15401057806297      0.00000000000000      c
    4.10256322732066     -0.15006390286587      0.00000000000000      h
   -3.31705741578258      1.73160011289946      0.00000000000000      pd
   -2.68217020196162     -1.27351505390765      0.00000000000000      h
$end
```

product

```
$coord
   -4.35147119824507      0.12912096937751      0.00000000000000      h
   -2.34108667738856     -0.11171925058770      0.00000000000000      c
   -0.06882418883410     -0.53811194802463      0.00000000000000      c
    3.41450618691483     -1.17107087832785      0.00000000000000      pd
    3.34687587755291      1.69178110756266      0.00000000000000      h
$end
```

Reaction **5**:

reactant

```
$coord
   -1.26006909152356      0.00000000000000      0.00000000000000      c
    1.26006909152356      0.00000000000000      0.00000000000000      c
   -2.34104626031258      1.75888714176583      0.00000000000000      h
   -2.34104626031258     -1.75888714176583      0.00000000000000      h
    2.34104626031258      1.75888714176583      0.00000000000000      h
    2.34104626031258     -1.75888714176583      0.00000000000000      h
$end
```

reactant complex

```
$coord
    0.00000000000000     -1.33696977418930      0.31244701298007      c
    0.00000000000000      1.33696977418930      0.31244701298007      c
   -1.74831154462943     -2.37841124515008      0.69242579887308      h
   -1.74831154462943      2.37841124515008      0.69242579887308      h
    1.74831154462943      2.37841124515008      0.69242579887308      h
    1.74831154462943     -2.37841124515008      0.69242579887308      h
    0.00000000000000      0.00000000000000     -3.39459722145244      pd
$end
```

transition state

```
$coord
   -1.77573114285714      0.46584185714286     -0.17462600000000      c
    0.76562385714286      0.46584185714286     -0.17462600000000      c
   -2.81616714285714      2.25584785714286     -0.17462600000000      h
    1.79774785714286      2.25407185714286      0.01029400000000      h
    1.94256385714286     -1.76683714285714      1.70450700000000      h
   -2.88376114285714     -1.27209514285714     -0.19791600000000      h
    2.96972385714286     -2.40267114285714     -0.99300700000000      pd
$end
```

product

```
$coord
   -1.89141830454892      0.73826282333409      0.19401678420851      c
    0.13652323326766     -0.74821727009557      0.46546596158011      c
   -3.79077955846962     -0.03816743546567      0.48754919111663      h
   -0.02108516377330     -2.78223585074872      0.83567055215146      h
    3.77274127948578     -0.43509767723924     -2.19975784502221      h
   -1.76080836597552      2.73536333099749     -0.30327040339354      h
    3.55482688001395      0.53009207921762      0.52032575935903      pd
$end
```

Reaction **6**:

```
$coord
    0.00000000000000      0.00000000000000      0.00000000000000      b
    0.00000000000000     -1.96272843818143      1.13318179213018      h
    0.00000000000000      1.96272843818143      1.13318179213018      h
    0.00000000000000      0.00000000000000     -2.26636358426036      h
$end
```

reactant complex

```
$coord
   -0.51616030475977      0.43368323122043     -0.21621911712469      b
   -0.89618946123553      0.51329154212998     -2.46704355489373      h
    0.33532293466509     -1.73870062986279      1.23320783821123      h
   -1.98296308842399      1.46166735154021      1.18067313242577      h
    3.05998991975422     -0.66994149502783      0.26938170138142      pd
$end
```

transition state

```
$coord
    0.72394682993979     -0.39119817834646      0.27478610614027      b
   -2.95340655639671     -0.21871269440076      0.24174337430876      pd
    1.47432776961149     -2.34122509385018     -0.64835556137187      h
   -1.39985432696806      1.88483173221120     -1.13316065887795      h
    2.15498628381349      1.06630423438618      1.26498673980079      h
$end
```

product

```
$coord
    0.99616995433622     -0.48908757730341      0.00000000000000      b
   -2.61704629187946     -0.48024118947315      0.00000000000000      pd
    1.64277917251253     -2.70040899249164      0.00000000000000      h
   -2.46922599720029      2.42333773104699      0.00000000000000      h
    2.44732316223101      1.24640002822121      0.00000000000000      h
$end
```

Reaction **7**:

reactant

```
$coord
    0.00000000000000      0.00000000000000      0.70936651838074      h
    0.00000000000000      0.00000000000000     -0.70936651838074      h
$end
```

reactant complex

```
$coord
   -0.89305700020942      0.00000000000000     -0.97890477539465      h
    0.00000000000000      0.00000000000000      1.95780955078931      pd
    0.89305700020942      0.00000000000000     -0.97890477539465      h
$end
```

transition state

```
$coord
   -1.22797091329417      0.00000000000000     -0.88086795709859      h
    0.00000000000000      0.00000000000000      1.76173591419719      pd
    1.22797091329417      0.00000000000000     -0.88086795709859      h
$end
```

product

```
$coord
   -1.74872151327333      0.00000000000000     -0.75760102712099      h
    0.00000000000000      0.00000000000000      1.51520205424197      pd
    1.74872151327333      0.00000000000000     -0.75760102712099      h
$end
```

Reaction **8**:

reactant

```
$coord
    2.28959810067812     -1.32190007976257      0.00000000000000      c
    2.28959810067812      1.32190007976257      0.00000000000000      c
    0.00000000000000      2.64380015952514      0.00000000000000      c
   -2.28959810067812      1.32190007976257      0.00000000000000      c
   -2.28959810067812     -1.32190007976257      0.00000000000000      c
    0.00000000000000     -2.64380015952514      0.00000000000000      c
    4.07564049746586      2.35307213833206      0.00000000000000      h
    0.00000000000000      4.70614427666411      0.00000000000000      h
   -4.07564049746586      2.35307213833206      0.00000000000000      h
   -4.07564049746586     -2.35307213833206      0.00000000000000      h
    0.00000000000000     -4.70614427666411      0.00000000000000      h
    4.07564049746586     -2.35307213833206      0.00000000000000      h
$end
```

reactant complex

```
$coord
    1.42363758835454      1.57253522189086      1.36459099414013      c
    1.42363758835454      1.57253522189086     -1.36459099414013      c
   -0.30752063512963     -0.03499854873637     -2.66870009788697      c
   -1.99583141340813     -1.53857863108247     -1.33364314071334      c
   -1.99583141340813     -1.53857863108247      1.33364314071334      c
   -0.30752063512963     -0.03499854873637      2.66870009788697      c
    2.33152967401957      3.11364717471806     -2.40230813354086      h
   -0.33418863330778     -0.01523826721988     -4.73017863063568      h
   -3.33795258438803     -2.72514559307588     -2.35459956603454      h
   -3.33795258438803     -2.72514559307588      2.35459956603454      h
   -0.33418863330778     -0.01523826721988      4.73017863063568      h
    4.44065200771889     -0.74444271298869      0.00000000000000      pd
    2.33152967401957      3.11364717471806      2.40230813354086      h
$end
```

transition state

```
$coord
   -2.14487336820119     -0.01769515300271      0.00000000000000      c
   -0.79804712809629     -0.01769515300271      2.29791953034502      c
    1.84028811807891     -0.16075420743837      2.28676736398094      c
    3.16465497753224     -0.24049144915336      0.00000000000000      c
    1.84028811807891     -0.16075420743837     -2.28676736398094      c
   -0.79804712809629     -0.01769515300271     -2.29791953034502      c
```

```
   -1.82197557634548      0.07731306050587      4.08213418469380      h
    2.86395309843808     -0.19327352077676      4.07765359611721      h
    5.22434399280328     -0.33720535052670      0.00000000000000      h
    2.86395309843808     -0.19327352077676     -4.07765359611721      h
   -1.82197557634548      0.07731306050587     -4.08213418469380      h
   -5.80745365446578     -0.75955687799397      0.00000000000000      pd
   -4.60510897181904      1.94376847210069      0.00000000000000      h
$end
```

product

```
$coord
   -1.66042878038495     -1.17688305913066      0.00000000000000      c
   -0.54100804165745     -0.47254953318939      2.30232488044049      c
    1.75462084458250      0.84386673409449      2.28755917774019      c
    2.90545903503607      1.50023429012690      0.00000000000000      c
    1.75462084458250      0.84386673409449     -2.28755917774019      c
   -0.54100804165745     -0.47254953318939     -2.30232488044049      c
   -1.44676862460027     -0.95227005519118      4.08898598761483      h
    2.63160213187630      1.37059357788032      4.07928151306123      h
    4.68585092018269      2.54015177194486      0.00000000000000      h
    2.63160213187630      1.37059357788032     -4.07928151306123      h
   -1.44676862460027     -0.95227005519118     -4.08898598761483      h
   -4.56070627277751     -3.42580237184085      0.00000000000000      pd
   -6.16706752245851     -1.01698207828871      0.00000000000000      h
$end
```

Reaction **10**:

reactant

```
$coord
    0.00000000000000      0.00000000000000      0.00000000000000      c
    1.69480823375199      0.00000000000000      1.19841039489683      h
   -1.69480823375199      0.00000000000000      1.19841039489683      h
    0.00000000000000     -1.69480823375199     -1.19841039489683      h
    0.00000000000000      1.69480823375199     -1.19841039489683      h
$end
```

reactant complex

```
$coord
    0.00000000000000      0.00000000000000      0.69739503554454      c
    0.00000000000000      1.71541238380755      1.85670098992525      h
   -1.84094691578371      0.00000000000000     -0.43219634107662      h
    1.84094691578371      0.00000000000000     -0.43219634107662      h
    0.00000000000000     -1.71541238380755      1.85670098992525      h
    0.00000000000000      0.00000000000000     -3.54640433324179      pd
$end
```

transition state

```
$coord
    0.35107070350377     -0.61500160999319      0.00000000000000      c
    1.52679222518970     -0.71229107700964      1.70654697672554      h
   -1.00119235713010     -2.21281868580291      0.00000000000000      h
    0.24739337785861      2.34081207390340      0.00000000000000      h
    1.52679222518970     -0.71229107700964     -1.70654697672554      h
   -2.65085617461169      1.91159037591198      0.00000000000000      pd
$end
```

product

```
$coord
   -0.00041463820783     -0.91095735549307     -0.43803470501920      c
    1.67493886077509     -1.62140752163925      0.56450410966089      h
    0.00000621696540      2.70496326015712      2.38350977404208      h
    0.07217849477973     -1.42370949039595     -2.46534759904685      h
   -1.74741328291982     -1.61059145179714      0.44336764342215      h
    0.00070434860741      2.86170255916831     -0.48799922305908      pd
$end
```

Reaction **10**:

reactant

```
$coord
    0.00000000000000     -0.28802324859021      0.00000000000000      c
   -0.98271913757002     -0.94150820330141      1.70211947584155      h
   -0.98271913757002     -0.94150820330141     -1.70211947584155      h
    0.00000000000000      3.11254785849446      0.00000000000000      cl
    1.96543827514005     -0.94150820330141      0.00000000000000      h
$end
```

reactant complex

```
$coord
   -1.19143021594370      0.47314360246124      0.00000000000000      c
   -0.77776706619676      1.56237388183094      1.70554188027910      h
   -3.13515297119647     -0.24827114136621      0.00000000000000      h
   -0.77776706619676      1.56237388183094     -1.70554188027910      h
    0.90679634549739     -2.28276001059780      0.00000000000000      cl
    4.97532097403631     -1.06686021415911      0.00000000000000      pd
$end
```

transition state

```
$coord
    0.07073750000000      0.11986616666667     -0.96051350000000      c
    2.14225250000000      0.11986616666667     -0.96051350000000      h
   -0.79731950000000      1.99993516666667     -0.96051350000000      h
   -0.66827850000000     -1.04002983333333     -2.49791450000000      h
   -1.44727650000000     -2.21692583333333      1.79222750000000      cl
    0.69988450000000      1.01728816666667      3.58722750000000      pd
$end
```

product

```
$coord
    0.98210701879284     -0.00017203372681     -0.72916368785287      c
    3.04463595265758      0.02420997399137     -0.35812232943017      h
    0.34193790099326      1.70574495657980     -1.72189215071998      h
    0.37807722159085     -1.72952778526044     -1.70420628879175      h
   -4.39679986872112     -0.00013993464293      1.73718606316721      cl
   -0.34995822531343     -0.00011517694098      2.77619839362754      pd
$end
```

Reaction **11**:

reactant (see reaction **10** in this subsection)

reactant complex

```
$coord
  -0.43755301800523      0.37917571013978     -0.00150870698755      c
  -3.24661441376447     -1.55833863002622     -0.00167582525182      cl
   3.46713429113700     -1.22171609577754     -0.00261463155246      pd
   0.62044414231038      0.00217216770355      1.83900499718971      h
  -1.00447741356256      2.36689900541776      0.02009512112076      h
   0.60106641188486      0.03180784254269     -1.85330095451862      h
$end
```

transition state

```
$coord
  -0.32106350000000     -1.04948583333333      0.00026900000000      c
   3.43220450000000     -1.04948583333333      0.00026900000000      pd
  -0.61187250000000      4.73327216666667      0.00026900000000      cl
  -1.14977650000000     -1.86992483333333     -1.72361100000000      h
  -1.15543450000000     -1.89696083333333      1.70830500000000      h
  -0.19405750000000      1.13258516666667      0.01449900000000      h
$end
```

product

```
$coord
   0.98210701879284     -0.00017203372681     -0.72916368785287      c
   3.04463595265758      0.02420997399137     -0.35812232943017      h
   0.34193790099326      1.70574495657980     -1.72189215071998      h
   0.37807722159085     -1.72952778526044     -1.70420628879175      h
  -4.39679986872112     -0.00013993464293      1.73718606316721      cl
  -0.34995822531343     -0.00011517694098      2.77619839362754      pd
$end
```

Reaction **12**:

reactant

```
$coord
  -1.44835159317494      0.00000000000000      0.00000000000000      c
  -2.20082092875607      0.96968005219708     -1.67953511749139      h
   1.44835159317494      0.00000000000000      0.00000000000000      c
  -2.20082092875607      0.96968005219708      1.67953511749139      h
  -2.20082092875607     -1.93936010439417      0.00000000000000      h
   2.20082092875607     -0.96968005219708      1.67953511749139      h
   2.20082092875607     -0.96968005219708     -1.67953511749139      h
   2.20082092875607      1.93936010439417      0.00000000000000      h
$end
```

reactant complex

```
$coord
   0.94689002989886      0.53013261132874      0.00000000000000      c
   2.93346469437125     -0.06760505841549      0.00000000000000      h
   0.70990275009972      1.66750273544212      1.82437422654693      h
   0.70990275009972      1.66750273544212     -1.82437422654693      h
  -0.91394599458620     -1.68572974898759      0.00000000000000      c
  -0.21825143618085      4.64364342188302      0.00000000000000      pd
  -2.87097325434152     -0.99451998168905      0.00000000000000      h
  -0.64849476968051     -2.88046335750192     -1.68389461375626      h
  -0.64849476968051     -2.88046335750192      1.68389461375626      h
$end
```

transition state

```
$coord
    0.59908655555556    -0.12611744444444    -0.98271000000000      c
    2.67703855555556    -0.12611744444444    -0.98271000000000      h
    0.75760755555556     2.85011255555556    -0.98271000000000      h
    0.00698555555556    -1.49617244444444    -2.45263600000000      h
   -0.47806544444444    -0.79940144444444     1.61332200000000      c
   -1.31496744444444     2.65519755555556    -3.04979900000000      pd
   -2.55322844444444    -0.82823844444444     1.58344900000000      h
    0.18899755555556    -2.69156344444444     2.18908000000000      h
    0.11654555555556     0.56230055555556     3.06471400000000      h
$end
```

product

```
$coord
   -0.13045389107839    -1.22277970348715    -0.37605820819458      c
    1.61833007058631    -2.33692769615946    -0.21001941661086      h
    2.19679123508012     1.43254449297990    -3.50864554590436      h
   -1.77576691271240    -2.52650112735378    -0.39014839263893      h
   -0.35351794662995     0.76816625272148     1.68837968477085      c
   -0.32707317356750     0.09191989061404    -3.92187164427590      pd
   -2.05288132136448     1.93939023300571     1.45596378335922      h
   -0.47526076959942    -0.16807485350896     3.55477720864298      h
    1.29983270928568     2.02226251118823     1.70762253085157      h
$end
```

Reaction **13**:

reactant (see reaction **12** in this subsection)

reactant complex (see reaction **12** in this subsection)

transition state

```
$coord
   -1.80267379357525    -0.00139868900071    -0.24473427877190      c
    0.00000000000000     0.00000000000000     3.29873490301428      pd
    1.80267379357525     0.00139868900071    -0.24473427877190      c
   -3.27954815723374    -0.13091980021887     1.31361603860579      h
    2.16260010472354    -1.75241528067424    -1.27408250750175      h
   -2.07776548774804    -1.65946032681312    -1.44416670383927      h
    3.27954815723374     0.13091980021887     1.31361603860579      h
   -2.16260010472354     1.75241528067424    -1.27408250750175      h
    2.07776548774804     1.65946032681312    -1.44416670383927      h
$end
```

product

```
$coord
   -2.75610703591884     0.00002817958383     0.01642347298914      c
    0.00000000000000     0.00000000000000    -2.56799852881020      pd
    2.75610703591884    -0.00002817958383     0.01642347298914      c
   -2.67128044281850    -1.74866742347872     1.13630486941333      h
   -2.59709632664519     1.67421035732743     1.23692769077147      h
   -4.51926852541519     0.07278764449658    -1.10565676876886      h
    2.67128044281850     1.74866742347872     1.13630486941333      h
    4.51926852541519    -0.07278764449658    -1.10565676876886      h
    2.59709632664519    -1.67421035732743     1.23692769077147      h
$end
```

1.2 PdCl$^-$ subset

PdCl$^-$:

```
$coord
    0.00000000000000      0.00000000000000      2.17320161197838      pd
    0.00000000000000      0.00000000000000     -2.17320161197838      cl
$end
```

Reaction **1**:

reactant

```
$coord
    1.45072359481631      0.00000000000000      0.37482740519908      h
    0.00000000000000      0.00000000000000     -0.74965481039816      o
   -1.45072359481631      0.00000000000000      0.37482740519908      h
$end
```

reactant complex

```
$coord
   -2.10217453854800      1.60740562502493      0.15280757069570      h
    1.52413862026988     -0.31816571820635      0.51546082832853      pd
   -2.88787130244289     -0.06167494736917      0.37190260343336      o
   -2.37346759108653     -0.98573254820279     -1.14277579845749      h
    5.83937481180755     -0.24183241124662      0.10260479599990      cl
$end
```

transition state

```
$coord
   -0.92692749001820      1.51620522207651      0.36705820241922      h
    1.27146208947851     -0.38940606298952      0.27149716347948      pd
   -2.71363313337206     -0.61189958863946      0.52001703507125      o
   -3.23149499883343     -0.77850296377314     -1.22417825776164      h
    5.60059353274518      0.26360339332561      0.06560585679168      cl
$end
```

product

```
$coord
    0.15948875003202      2.39776999820642     -0.08818973025115      h
    0.87515507305506     -0.34963574563341     -0.28277793961125      pd
   -2.68816321452702     -1.57565136547427     -0.36910681617482      o
   -3.56581672775315     -0.92931450798390      1.09034856220463      h
    5.21933611919308      0.45683162088516     -0.35027407616740      cl
$end
```

Reaction **2**:

reactant

```
$coord
    0.00000000000000      0.00000000000000     -0.54710229857343      n
    0.89460466361279     -1.54950073006542      0.18236743285780      h
    0.89460466361279      1.54950073006542      0.18236743285780      h
   -1.78920932722558      0.00000000000000      0.18236743285780      h
$end
```

reactant complex

```
$coord
    0.00000000000000      0.00000000000000      1.74976559718743      n
    0.00000000000000      0.00000000000000     -2.24654177052174      pd
   -0.90743927909200     -1.57173093617102      2.38221200839304      h
   -0.90743927909200      1.57173093617102      2.38221200839304      h
    1.81487855818400      0.00000000000000      2.38221200839304      h
    0.00000000000000      0.00000000000000     -6.64131413129738      cl
$end
```

transition state

```
$coord
   -1.53068319270606      1.45384766565851      0.00000000000000      n
    1.89243052213277     -0.43440687647542      0.00000000000000      pd
   -2.66753358709377      1.34826978179794      1.54237571784440      h
   -0.90444519250601     -1.34048776705358      0.00000000000000      h
   -2.66753358709377      1.34826978179794     -1.54237571784440      h
    5.87776503726686     -2.37549258572539      0.00000000000000      cl
$end
```

product

```
$coord
    2.17360706810022      1.21603570623573      0.00000000000000      n
    3.09579753440413      0.46750454512766     -1.52018976368183      h
    3.09579753440413      0.46750454512766      1.52018976368183      h
   -1.49623109985010      0.29940486616110      0.00000000000000      pd
   -0.89646744837229     -2.49982606023891      0.00000000000000      h
   -5.97250358868609      0.04937639758675      0.00000000000000      cl
$end
```

Reaction **3**:

reactant

```
$coord
   -0.82199483857914      1.42373682397845      0.00000000000000      c
   -0.82199483857914     -1.42373682397845      0.00000000000000      c
    1.64398967715828      0.00000000000000      0.00000000000000      c
   -1.37663541506102      2.38440248238436      1.70739318539849      h
   -1.37663541506102      2.38440248238436     -1.70739318539849      h
   -1.37663541506102     -2.38440248238436      1.70739318539849      h
   -1.37663541506102     -2.38440248238436     -1.70739318539849      h
    2.75327083012204      0.00000000000000     -1.70739318539849      h
    2.75327083012204      0.00000000000000      1.70739318539849      h
$end
```

reactant complex

```
$coord
   -2.27081465069178      0.25731722999473      1.43409454426326      c
   -2.27081465069178      0.25731722999473     -1.43409454426326      c
    0.11262959161354     -0.31331071333788      0.00000000000000      c
   -3.22196767345355     -1.28425927740745      2.39791170346217      h
   -2.41684305188617      2.06323773162390      2.39551379290609      h
   -3.22196767345355     -1.28425927740745     -2.39791170346217      h
   -2.41684305188617      2.06323773162390     -2.39551379290609      h
    1.52213641339553      1.30893762053701      0.00000000000000      h
    0.77531947194848     -2.26355298406228      0.00000000000000      h
    4.50934436922797     -0.23155256591121      0.00000000000000      pd
    8.89969632751141     -0.57308105916536      0.00000000000000      cl
$end
```

transition state

```
$coord
    1.61177181466859     -1.08860739840464      1.30413444193545      c
    2.22583175494534      1.32661594565519     -0.06033269977843      c
    0.07224521856409     -0.31948689037790     -1.01351559682470      c
    0.72165237108438     -0.97722637534903      3.14271062017970      h
    2.91723461751459     -2.66260534080612      1.09172863974398      h
    1.72445421351251      3.08934066442744      0.85896292414501      h
    3.96743557415801      1.41055415537917     -1.14798923361416      h
    0.57095858865658     -1.36484966023719     -2.70985255407373      h
   -1.95754848123805      1.66222494655326     -1.76261227620277      h
   -3.79974244495253     -0.13781359654476     -0.42077489962711      pd
   -8.05429322691356     -0.93814645029540      0.71754063411671      cl
$end
```

product

```
$coord
   -0.78536983897511      2.29671523338873     -0.48385881686404      c
   -2.29864448106552      0.38612516065221      1.01932671488815      c
   -0.40593931888982     -0.51913342171527     -0.91304292406866      c
    0.68438133261430      3.33431736247915      0.50516903904383      h
   -1.76072834588096      3.40179274015938     -1.91806171545071      h
   -1.83315445402644      0.16692698562049      3.00410645290759      h
   -4.30900867556492      0.22420917683595      0.61083448209868      h
   -1.27935350972427     -1.21599918595961     -2.63905295384193      h
    1.43936696751534     -4.25150927906072     -0.01833681866158      h
    3.05009612437000     -1.86720196870204     -0.03707034854030      pd
    7.49835419962743     -1.95624280369829      0.86998688848901      cl
$end
```

Reaction **4**:

reactant

```
$coord
    0.00000000000000      0.00000000000000     -1.13159388828988      c
    0.00000000000000      0.00000000000000      1.13159388828988      c
    0.00000000000000      0.00000000000000      3.14170756905486      h
    0.00000000000000      0.00000000000000     -3.14170756905486      h
$end
```

reactant complex

```
$coord
    2.53970017972549      3.01653100565048      0.00000000000000      h
    1.63581590151085      1.20071646145272      0.00000000000000      c
    1.67387313678193     -1.17946214182372      0.00000000000000      c
   -2.00477811713876     -0.03840800409108      0.00000000000000      pd
    2.64919999672690     -2.95832848623527      0.00000000000000      h
   -6.49381109760643     -0.04104883495312      0.00000000000000      cl
$end
```

transition state

```
$coord
    1.08846769145371     -0.28172395690704      0.00000000000000      c
    3.24825038715398     -1.09566916734713      0.00000000000000      c
    5.03413074793577     -2.01429016133426      0.00000000000000      h
   -2.41809719302310      0.63810868788240      0.00000000000000      pd
   -0.08707199431672      2.40922521847272      0.00000000000000      h
   -6.86567963920364      0.34434937923331      0.00000000000000      cl
$end
```

product

```
$coord
   -5.17450703580183      2.47509720869943      0.00000000000000      h
   -3.33244779185206      1.67235811800400      0.00000000000000      c
   -1.19648343191744      0.80278135732128      0.00000000000000      c
    2.24984302915308     -0.46954377690046      0.00000000000000      pd
    0.87252539506634     -2.95096909035325      0.00000000000000      h
    6.58106983535192     -1.52972381677100      0.00000000000000      cl
$end
```

Reaction **4**:

reactant

```
$coord
   -1.25259928081358      0.00000000000000      0.00000000000000      c
    1.25259928081358      0.00000000000000      0.00000000000000      c
   -2.32604772825070      1.74587640693775      0.00000000000000      h
   -2.32604772825070     -1.74587640693775      0.00000000000000      h
    2.32604772825070      1.74587640693775      0.00000000000000      h
    2.32604772825070     -1.74587640693775      0.00000000000000      h
$end
```

reactant complex

```
$coord
    0.00000000000000      1.32459969448941      1.29289060704326      c
    0.00000000000000     -1.32459969448941      1.29289060704326      c
    1.73021529464738      2.36367318934035      1.69130178185321      h
    1.73021529464738     -2.36367318934035      1.69130178185321      h
   -1.73021529464738     -2.36367318934035      1.69130178185321      h
   -1.73021529464738      2.36367318934035      1.69130178185321      h
    0.00000000000000      0.00000000000000     -2.44276500102434      pd
    0.00000000000000      0.00000000000000     -6.92134377749718      cl
$end
```

transition state

```
$coord
    2.50621374797210      1.14312294878038      0.13897079140571      c
    0.73621943359845     -0.49182736384742     -0.65212507892783      c
    4.32486596977039      1.30170958759376     -0.81684588008232      h
    1.29578297349242     -1.75919030812936     -2.18653614930244      h
   -0.80725868354118     -2.02177572312146      1.70330304190308      h
    2.18602004341598      2.40799434500655      1.72138716728956      h
   -2.91197051828161     -0.67105035921147      0.19976137532392      pd
   -7.32987296642655      0.09101687292905     -0.10791526760967      cl
$end
```

product

```
$coord
   -2.55270552101439     -1.20156361125546      0.15571639629439      c
   -0.81588486949659      0.42894457774803     -0.69937710727313      c
   -4.42002666275422     -1.33749512758102     -0.70989348810177      h
   -1.42842673743244      1.69494694696257     -2.22038705398201      h
    1.32014673437287      2.07120488377232      2.15800125146667      h
   -2.17496383051722     -2.48330199193151      1.71315907543635      h
    2.81158317378781      0.71019285081602      0.09999074271002      pd
    7.26027771305418      0.11707147146903     -0.49720981655052      cl
$end
```

Reaction **8**:

reactant

```
$coord
    2.27870002626172     -1.31560807356461      0.00000000000000      c
    2.27870002626172      1.31560807356461      0.00000000000000      c
    0.00000000000000      2.63121614712922      0.00000000000000      c
   -2.27870002626172      1.31560807356461      0.00000000000000      c
   -2.27870002626172     -1.31560807356461      0.00000000000000      c
    0.00000000000000     -2.63121614712922      0.00000000000000      c
    4.05152957138905      2.33915168867120      0.00000000000000      h
    0.00000000000000      4.67830337734240      0.00000000000000      h
   -4.05152957138905      2.33915168867120      0.00000000000000      h
   -4.05152957138905     -2.33915168867120      0.00000000000000      h
    0.00000000000000     -4.67830337734240      0.00000000000000      h
    4.05152957138905     -2.33915168867120      0.00000000000000      h
$end
```

reactant complex

```
$coord
   -0.36224533548797     -1.90683333826320      1.35087092616165      c
   -0.36224533548797     -1.90683333826320     -1.35087092616165      c
    0.94229699004816      0.06615041991319     -2.64688506702312      c
    2.20083420598125      1.92962254488923     -1.33580040484713      c
    2.20083420598125      1.92962254488923      1.33580040484713      c
    0.94229699004816      0.06615041991319      2.64688506702312      c
   -0.81200879841765     -3.61820003584605     -2.39430524220421      h
    0.95166860769042      0.06506087156405     -4.69650127469811      h
    3.17861451702349      3.41658537508884     -2.35265422882410      h
    3.17861451702349      3.41658537508884      2.35265422882410      h
    0.95166860769042      0.06506087156405      4.69650127469811      h
   -3.95401437059534     -0.61225933883672      0.00000000000000      pd
   -0.81200879841765     -3.61820003584605      2.39430524220421      h
   -8.23791938175022      0.70280213915274      0.00000000000000      cl
$end
```

transition state

```
$coord
   -0.79250806694503     -1.30885366187841      0.18985219751464      c
    1.86817813639807     -1.28827361730188      0.20698813409680      c
    3.22639176372686      0.96384080028047      0.20547247464462      c
    1.97588480829657      3.28078825722368      0.27005515747250      c
   -0.65598182060514      3.30270603901577      0.36096322667980      c
   -2.01298358511675      1.04912529614356      0.36037986114854      c
    2.86732804254943     -3.07345076186467      0.15514355504895      h
    5.27648211543890      0.90715541098869      0.14662090153889      h
    3.03312531597450      5.03605014236247      0.25807913574997      h
   -1.66311203359779      5.08909877732651      0.42671123231866      h
   -4.05770822093214      1.09909304498384      0.43078065886793      h
   -2.65155068046079     -4.38492518745336     -0.87372510588850      pd
   -2.12987200088020     -3.59603141109934      1.91540339134212      h
   -4.28367377384654     -7.07632312872736     -4.05272482053489      cl
$end
```

product

```
$coord
   -1.37936068709021     -0.32667882737638      0.00000000000000      c
   -0.00034945368742     -0.06838103076205     -2.26000713686739      c
    2.56892999286046      0.51129598834876     -2.26637565868593      c
    3.87502118677203      0.81141837472569      0.00000000000000      c
    2.56892999286046      0.51129598834876      2.26637565868593      c
   -0.00034945368742     -0.06838103076205      2.26000713686739      c
```

```
   -0.97185473358459     -0.30609291555224     -4.04763197594265      h
    3.55891404366643      0.71955882293321     -4.05234669557837      h
    5.87537114358859      1.25974596103455      0.00000000000000      h
    3.55891404366643      0.71955882293321      4.05234669557837      h
   -0.97185473358459     -0.30609291555224      4.04763197594265      h
   -5.12192836743827     -0.63837355172516      0.00000000000000      pd
   -4.07811406549384     -3.32187200938801      0.00000000000000      h
   -9.48223058531051      0.50298993560871      0.00000000000000      cl
$end
```

Reaction **9**:

reactant

```
$coord
    0.00000000000000      0.00000000000000      0.00000000000000      c
    1.14755402783870     -1.14755402783870      1.14755402783870      h
   -1.14755402783870      1.14755402783870      1.14755402783870      h
   -1.14755402783870     -1.14755402783870     -1.14755402783870      h
    1.14755402783870      1.14755402783870     -1.14755402783870      h
$end
```

reactant complex

```
$coord
    0.00000000000000      0.00000000000000     -1.88908753537979      c
    0.00000000000000     -1.69937923783696     -3.05434362892248      h
   -1.77680567426165      0.00000000000000     -0.71270346673687      h
    1.77680567426165      0.00000000000000     -0.71270346673687      h
    0.00000000000000      1.69937923783696     -3.05434362892248      h
    0.00000000000000      0.00000000000000      2.50307578249009      pd
    0.00000000000000      0.00000000000000      6.90824067841860      cl
$end
```

transition state

```
$coord
    0.77508549197841      0.37861139843259      1.51807217134445      c
    2.84110953103764      0.40714528761925      1.58827212026907      h
    0.09302294395800      2.34012493416131      1.46564576370333      h
    0.58418422837771     -2.07292472102232     -0.12609708068749      h
    0.06800241434979     -0.50978580073694      3.24589086445538      h
   -1.00085950021252     -0.45950386839352     -1.94805591771224      pd
   -3.36054510948904     -0.08366723006034     -5.74372792137247      cl
$end
```

product

```
$coord
    1.56642531085289      0.95907043546344     -0.00063618201901      c
    2.75878007689584      0.51389220270910      1.63247648831433      h
    0.16125192701409     -2.94461404398504      0.01775290283463      h
    1.05113176967767      2.97433668579070      0.07867293416523      h
    2.66865372751977      0.61954859725916     -1.72111482941835      h
   -1.83131346038841     -0.87717952609556      0.00308574278304      pd
   -6.37492935157188     -1.24505435114180     -0.01023705665990      cl
$end
```

Reaction **10**:

reactant

```
$coord
    0.00000000000000      0.00000000000000     -0.29204314373951      c
   -0.97418393958675     -1.68733607928186     -0.93688390600618      h
   -0.97418393958675      1.68733607928186     -0.93688390600618      h
    0.00000000000000      0.00000000000000      3.10269486175804      cl
    1.94836787917350      0.00000000000000     -0.93688390600618      h
$end
```

reactant complex

```
$coord
    2.55117141884418     -0.18072161832027      0.00000000000000      c
    2.22746661555778     -1.30704686953100      1.68318227188524      h
    4.42989325500496      0.67465707750000      0.00000000000000      h
    2.22746661555778     -1.30704686953100     -1.68318227188524      h
    0.19292819715605      2.35027113652412      0.00000000000000      cl
   -3.75809893030192      0.68005716126491      0.00000000000000      pd
   -7.87082717181882     -0.91017001790678      0.00000000000000      cl
$end
```

transition state

```
$coord
   -1.41026483920261      1.37482479371247      0.00000000000000      c
   -0.37877361845219      1.81444850373039     -1.70798235984115      h
   -0.37877361845219      1.81444850373039      1.70798235984115      h
   -3.33175228937279      2.08936930599081      0.00000000000000      h
   -2.54866000211171     -2.40551592386353      0.00000000000000      cl
    1.80217688366253     -2.47569165809396      0.00000000000000      pd
    6.24604748392896     -2.21188352520656      0.00000000000000      cl
$end
```

product

```
$coord
    0.10110656756335     -1.45325755074951     -0.00093503494414      c
    2.09106516692256     -1.96841763212796     -0.02404030856165      h
   -0.88940212900773     -2.10743669631297     -1.68432726737387      h
   -0.84806063614974     -2.10461208618155      1.70740141032670      h
   -4.54488465630055      2.32660858187109      0.00056731138781      cl
   -0.13172413370055      2.31382459856016      0.00053094209956      pd
    4.22189982067265      2.99329078494076      0.00080294706560      cl
$end
```

Reaction **12**:

reactant

```
$coord
    0.00000000000000      0.00000000000000      1.44547312142977      c
   -1.66886275029181      0.96351835812152      2.19076438446940      h
    0.00000000000000      0.00000000000000     -1.44547312142977      c
    1.66886275029181      0.96351835812152      2.19076438446940      h
    0.00000000000000     -1.92703671624303      2.19076438446940      h
    1.66886275029181     -0.96351835812152     -2.19076438446940      h
   -1.66886275029181     -0.96351835812152     -2.19076438446940      h
    0.00000000000000      1.92703671624303     -2.19076438446940      h
$end
```

reactant complex

```
$coord
   -0.57562791041616     -1.06987432859012      0.00000000000000      c
   -1.53263526538865     -2.89801544821957      0.00000000000000      h
    0.62310534821747     -1.01659824820660      1.75876824897562      h
    0.62310534821747     -1.01659824820660     -1.75876824897562      h
   -2.42592120296822      1.14604678211364      0.00000000000000      c
    3.80808834658088     -0.50401764913640      0.00000000000000      pd
   -1.38159947274955      2.92461454040228      0.00000000000000      h
   -3.64616734548501      1.11895040967410     -1.67125911766003      h
   -3.64616734548501      1.11895040967410      1.67125911766003      h
    8.15404454460386      0.19450536329058      0.00000000000000      cl
$end
```

transition state

```
$coord
    0.45390572759709     -0.09387544855452      1.16062238862881      c
   -3.06567206072960     -1.57780018636039      0.04456200514775      pd
   -7.48999248543724     -1.53759334510218     -0.69760734056280      cl
    2.03335530208694      1.17718267067928     -0.90894844759784      c
    1.69128255774572     -1.09227860927585      2.48292553723226      h
   -0.37720461466293     -2.72533267601470     -0.02556759034509      h
   -0.55251056258643      1.33731168522952      2.27865575784270      h
    0.81172771686520      1.95453365779694     -2.37843284723759      h
    3.18340665573274      2.72883118999171     -0.13651143962577      h
    3.31170176338854     -0.17097893838977     -1.81969802348247      h
$end
```

product

```
$coord
    0.37804743941177      0.16687683324757      1.23092592933825      c
    1.29761458098161     -1.09433113655819      2.58829184884521      h
   -1.27391273782179     -3.34209047898975     -0.23400752626621      h
   -0.15524129519116      1.89530608560877      2.26194036044871      h
    2.22897400912003      0.82255023871559     -0.89619405438812      c
   -3.08148325601816     -1.11086010822352      0.07493514649798      pd
    1.34091284219714      2.04319840214700     -2.30846292983182      h
    3.91650241290613      1.81304886065828     -0.17595336002021      h
    2.86133126185454     -0.88222377645577     -1.87714152177780      h
   -7.51274525744014     -0.31147492014994     -0.66433389284598      cl
$end
```

Reaction **13**:

reactant (see reaction **12** in this subsection)

reactant complex (see reaction **12** in this subsection)

transition state

```
$coord
   -1.76515678595439     -0.00095148699459     -1.03986681024286      c
    0.00000000000000      0.00000000000000      2.62658801272905      pd
    1.76515678595439      0.00095148699459     -1.03986681024286      c
   -3.21898721515412     -0.34895631380604      0.45453036085062      h
    2.19577430574125     -1.80527873948144     -1.90845867155243      h
   -1.95887658910415     -1.52150115163372     -2.41308320989592      h
    3.21898721515412      0.34895631380604      0.45453036085062      h
   -2.19577430574125      1.80527873948144     -1.90845867155243      h
    1.95887658910415      1.52150115163372     -2.41308320989592      h
    0.00000000000000      0.00000000000000      7.14240316768797      cl
$end
```

product

```
$coord
    0.32121525675765      2.44900612515763      0.00661652994041      c
   -2.12785281558077     -0.48559374054556      0.00299363501068      pd
    1.08191069958462     -2.65616120924952     -0.01192429983659      c
    1.41994864339888      2.44921446236788      1.75911800092778      h
    1.60677206524531      2.33357575773554     -1.60876537213630      h
   -0.84855540577585      4.15022055848244     -0.11468280557237      h
    2.18502415436571     -2.39246677567480     -1.74811544047247      h
    0.48973875027323     -4.64992324803763      0.09495721509722      h
    2.31617870552216     -2.25276611126790      1.60441135109398      h
   -6.44438005379097      1.05489418103189      0.01539118594764      cl
$end
```

1.3 $PdCl_2$

$PdCl_2$:

```
$coord
    0.00000000000000      0.00000000000000     -1.71451458045675      pd
   -3.21019596503499      0.00000000000000      0.85723845462232      cl
    3.21019596503499      0.00000000000000      0.85723845462232      cl
$end
```

Reaction **1**:

reactant

```
$coord
    1.45073772696982      0.00000000000000      0.37481785522568      h
    0.00000000000000      0.00000000000000     -0.74963571044679      o
   -1.45073772696982      0.00000000000000      0.37481785522568      h
$end
```

reactant complex

```
$coord
   -1.25339380309977     -1.23773916458562      0.00000000000000      o
   -0.84641329008523     -2.22403953613488     -1.49874666584244      h
   -0.84641329008523     -2.22403953613488      1.49874666584244      h
    0.91011388272141      1.91939068302144      0.00000000000000      pd
    1.01241501787227      1.89224022024587     -4.25060383463287      cl
    1.01241501787227      1.89224022024587      4.25060383463287      cl
$end
```

transition state

```
$coord
   -1.47369884027884     -0.35034971541780      1.50703946268169      o
    1.24021174859478     -0.19531869045355      1.40945574860004      h
   -1.96573781487180      1.43234179000387      1.63767061017721      h
    0.64953647701029     -0.31579217569828     -1.41366037587344      pd
    1.35769550391253     -4.45309462271919     -1.41557392499030      cl
    0.19199292563306      3.88221341428495     -1.72493152059520      cl
$end
```

product

```
$coord
   -2.09366101831246     -0.32110107214495      1.35567229337680      o
    2.50246396705705     -0.16577272163858      0.87109035210342      h
   -2.37633675800664      1.49852379007897      1.58678986728536      h
    0.52509075248603     -0.36011735614880     -1.11060001038675      pd
    1.28462831349833     -4.50197073944442     -1.28636160452405      cl
    0.15781474327766      3.85043809929776     -1.41659089785476      cl
$end
```

Reaction **3**:

reactant

```
$coord
   -0.82523061578934      1.42934135450848      0.00000000000000      c
   -0.82523061578934     -1.42934135450848      0.00000000000000      c
    1.65046123157867      0.00000000000000      0.00000000000000      c
   -1.38344132912498      2.39619067133508      1.73354298999174      h
   -1.38344132912498      2.39619067133508     -1.73354298999174      h
   -1.38344132912498     -2.39619067133508      1.73354298999174      h
   -1.38344132912498     -2.39619067133508     -1.73354298999174      h
    2.76688265824995      0.00000000000000     -1.73354298999174      h
    2.76688265824995      0.00000000000000      1.73354298999174      h
$end
```

reactant complex

```
$coord
   -2.11050845820140      0.11678033830910      0.73234761771333      c
    0.84731957373262     -0.02606620325703      0.73405487876265      c
   -0.73317127275772      2.51381292535321      0.70483319774353      c
   -0.65216439135751      3.55671294721709     -1.06917502998543      h
   -0.49090870569261      3.60193422564662      2.43905942769089      h
   -2.89820075944838     -0.57067160529863      2.50704468920701      h
   -3.08180079464983     -0.46205967736650     -0.98476179554620      h
    1.72654061912690     -0.54546787732805      2.51852620201658      h
    2.27835368080357      0.43343360628463     -0.85421256160017      h
    1.68615780680851     -2.73842946181402     -2.10403780456238      pd
   -0.19557982697775     -0.58636082492901     -5.34378529846738      cl
    3.62396252861356     -5.29361839281743      0.72010647702756      cl
$end
```

transition state

```
$coord
   -2.34761940674198     -0.27865467834322      0.83676318844220      c
    0.50250112690688     -0.31367372667154      0.83398721942745      c
   -0.92400689246440      2.19251729744901      0.83655024055694      c
   -0.97907077662967      3.25507246969610     -0.92622600463346      h
   -0.76178379716836      3.30856778498197      2.56426981254236      h
   -3.22440945476196     -0.95350902447997      2.57896761323591      h
   -3.32704724936269     -0.75909213378318     -0.90766230166211      h
    1.50508506004924     -0.90405991641222      2.53184833876464      h
    2.74893258823681      0.61948349729780     -0.99979775448678      h
    2.18558151674818     -1.93119485219482     -2.25894934629767      pd
   -0.26264639968412     -0.02967232127121     -5.24545064478260      cl
    4.88448368487208     -4.20578439626877      0.15569963889312      cl
$end
```

product

```
$coord
   -2.48450392275615     -0.33161384642005      0.94613930655357      c
    0.33800810925397     -0.32141374567849      0.95392025354471      c
   -0.97086264134378      2.16156606061812      0.94648427696585      c
   -1.10524438061250      3.21812036621822     -0.81529183458126      h
   -0.81122178957694      3.29783844019943      2.66382965091412      h
   -3.41865669042331     -1.02548036332743      2.65227406226028      h
   -3.47881961776298     -0.69243060230934     -0.82198897103466      h
    1.39500582632766     -0.94990842358091      2.60976888224338      h
    3.98094897581897      0.34766568970967     -2.05114998666430      h
    2.09343977695373     -1.76852534233020     -2.15184191855902      pd
   -0.23032826380010      0.33433998367372     -5.15886471482351      cl
    4.69223461792143     -4.27015821677272      0.22672099318081      cl
$end
```

Reaction **5**:

reactant

```
$coord
   -1.26006909152356      0.00000000000000      0.00000000000000      c
    1.26006909152356      0.00000000000000      0.00000000000000      c
   -2.34104626031258      1.75888714176583      0.00000000000000      h
   -2.34104626031258     -1.75888714176583      0.00000000000000      h
    2.34104626031258      1.75888714176584      0.00000000000000      h
    2.34104626031258     -1.75888714176584      0.00000000000000      h
$end
```

reactant complex

```
$coord
   -1.62023768722985     -0.53134363603019      1.31433324067739      c
   -1.62023768722985     -0.53134363603019     -1.31433324067739      c
   -0.94117939464297     -2.17489357355260      2.35952443263447      h
   -2.72578266990339      0.85400935232672      2.38071415006233      h
   -2.72578266990339      0.85400935232672     -2.38071415006233      h
   -0.94117939464297     -2.17489357355260     -2.35952443263447      h
    1.69627582039925      1.49736941637436      0.00000000000000      pd
    3.97841359769222     -2.06242857952769      0.00000000000000      cl
    4.90007673157803      4.26974552013908      0.00000000000000      cl
$end
```

transition state

```
$coord
   -2.79900230816272     -0.05733813198762      0.33938730596836      c
   -0.44174807298454     -0.95050176671478      0.24971588091813      c
   -3.40767700915593      1.85344566380542     -0.12463089180243      h
   -4.25033786124121     -1.39566856648403      0.97600579780562      h
    0.04888969644933     -2.84980806647667      0.87757195048593      h
    1.19898288027783      1.64427562104638      1.18563228311283      h
    2.83592081818683      0.55288886365150     -0.94998074479404      pd
    5.27430674777099     -2.25040646333326      1.21885584956841      cl
    1.54066510885941      3.45311284649309     -3.77255743126281      cl
$end
```

product

```
$coord
   -2.75704504886035     -0.11874803731222      0.29068972198074      c
   -0.53240640724130     -1.24248457248153      0.08440959376209      c
   -3.06341505133678      1.88929759861341     -0.03295061310434      h
   -4.38909085110328     -1.29561658348560      0.78749002140227      h
```

```
   -0.08097685225193     -3.22622221558864      0.42738657682508      h
    2.16572564913267      2.17150650938649      1.40311979406171      h
    2.64338707461246      0.53714635258819     -0.86831163057403      pd
    5.11636239577416     -2.07709923231375      1.45704572795132      cl
    0.89745909127433      3.36222018059366     -3.54887919230483      cl
$end
```

Reaction **7**:

reactant

```
$coord
    0.00000000000000      0.00000000000000      0.70937774792599      h
    0.00000000000000      0.00000000000000     -0.70937774792599      h
$end
```

reactant complex

```
$coord
    0.00000000000000      0.00000000000000      1.06551450310376      pd
    0.00000000000000      4.22972848799004      1.31036578653820      cl
    0.00000000000000     -4.22972848799004      1.31036578653820      cl
   -0.85461521829877      0.00000000000000     -1.84196068607428      h
    0.85461521829877      0.00000000000000     -1.84196068607428      h
$end
```

transition state

```
$coord
    0.00000000000000      0.00000000000000      0.99280815451614      pd
    0.00000000000000      4.22994449672436      1.17864445917411      cl
    0.00000000000000     -4.22994449672436      1.17864445917411      cl
   -1.06776976293509      0.00000000000000     -1.67628140956178      h
    1.06776976293509      0.00000000000000     -1.67628140956178      h
$end
```

product

```
$coord
    0.00000000000000      0.00000000000000      0.81446402031493      pd
    0.00000000000000      4.22050413149606      0.91856400824367      cl
    0.00000000000000     -4.22050413149606      0.91856400824367      cl
   -1.78824703393223      0.00000000000000     -1.32619815953895      h
    1.78824703393223      0.00000000000000     -1.32619815953895      h
$end
```

Reaction **8**:

reactant

```
$coord
    2.28959810067812     -1.32190007976257      0.00000000000000      c
    2.28959810067812      1.32190007976257      0.00000000000000      c
    0.00000000000000      2.64380015952514      0.00000000000000      c
   -2.28959810067812      1.32190007976257      0.00000000000000      c
   -2.28959810067812     -1.32190007976257      0.00000000000000      c
    0.00000000000000     -2.64380015952514      0.00000000000000      c
    4.07564049746586      2.35307213833206      0.00000000000000      h
```

```
   0.00000000000000      4.70614427666411      0.00000000000000      h
  -4.07564049746586      2.35307213833206      0.00000000000000      h
  -4.07564049746586     -2.35307213833206      0.00000000000000      h
   0.00000000000000     -4.70614427666411      0.00000000000000      h
   4.07564049746586     -2.35307213833206      0.00000000000000      h
$end
```

reactant complex

```
$coord
  -0.14849119608352     -3.08985781538413     -1.34320199104637      c
  -0.14849119608352     -3.08985781538413      1.34320199104637      c
   0.45450089255437     -0.94136662663638      2.68282525641240      c
   1.15485326168365      1.30898365043947      1.36702504986418      c
   1.15485326168365      1.30898365043947     -1.36702504986418      c
   0.45450089255437     -0.94136662663638     -2.68282525641240      c
  -0.63857530894883     -4.82118239568552      2.34661592617342      h
   0.46324420656265     -0.94587738716737      4.74197645919361      h
   2.19175149877227      2.78489927527486      2.37247624438949      h
   2.19175149877227      2.78489927527486     -2.37247624438949      h
   0.46324420656265     -0.94587738716737     -4.74197645919361      h
  -0.63857530894883     -4.82118239568552     -2.34661592617342      h
  -2.06054204861404      3.56331355623683      0.00000000000000      pd
  -5.28175232755492      0.74110057258268      0.00000000000000      cl
   0.38772766708775      7.10438846949860      0.00000000000000      cl
$end
```

transition state

```
$coord
  -0.45081154452577     -3.50517941858846     -0.07931833732824      c
   2.18816975132822     -3.37035083228119      0.01673303907409      c
   3.38783740854509     -1.02261272896939      0.11540753666501      c
   1.97001845279816      1.20943705065755      0.15109667665853      c
  -0.67285701982428      1.02696120516454      0.06457978085581      c
  -1.91572355201216     -1.30696022992578     -0.07740657986713      c
   3.30783546596212     -5.10096208802832      0.00650329269860      h
   5.44446217795292     -0.89772044223195      0.18350729239947      h
   2.88893043439194      3.04289699708222      0.25922536303329      h
  -1.37019026017012      3.49228555370732     -1.93765929019069      h
  -3.96407598282424     -1.40572407997310     -0.18988598371785      h
  -1.39982396131716     -5.33306879894959     -0.17624083156671      h
  -2.89599207065371      4.11767732325085      0.45018077191844      pd
  -6.32648656770163      2.87027551197036     -1.81988293784212      cl
  -0.19129273194932      6.18304497711500      3.03316020720952      cl
$end
```

product

```
$coord
  -0.46865908360202     -3.60892626491623      0.26135179448847      c
   2.16553613904493     -3.44957150054848      0.27929856670409      c
   3.35008767529948     -1.09336018431225      0.15967717933565      c
   1.91833775079792      1.13206536679476      0.03196690999620      c
  -0.69925338489997      0.89447063858893      0.01862826518006      c
  -1.95177924339331     -1.41642077415924      0.11182567508400      c
   3.29894324014516     -5.16747386077973      0.38635092619653      h
   5.40640318535395     -0.95003749305659      0.19586214035747      h
   2.82685218337432      2.97463344319482      0.01219329369699      h
  -1.18310703741178      5.12479190194049     -1.94009706076700      h
  -4.00253665793169     -1.53667252952869      0.03113870548422      h
  -1.41095762320238     -5.44165820373809      0.32987418370505      h
  -2.89204155514307      3.97162958385673      0.00873382574138      pd
  -5.51678062247211      2.70832584598029     -3.13123529324472      cl
  -0.84104496595946      5.85820403068332      3.24443088804158      cl
$end
```

Reaction **9**:

reactant

```
$coord
    0.00000000000000      0.00000000000000      0.00000000000000      c
    1.19841039489683     -1.19841039489683      1.19841039489683      h
   -1.19841039489683      1.19841039489683      1.19841039489683      h
   -1.19841039489683     -1.19841039489683     -1.19841039489683      h
    1.19841039489683      1.19841039489683     -1.19841039489683      h
$end
```

reactant complex

```
$coord
   -1.10313795744367     -1.22610465995245     -0.13432081403634      c
    1.14817735782320     -1.24911041201604     -0.08745743420449      h
   -2.28307087568204      0.50006101883723     -0.02234751854399      h
   -1.42969610209546     -2.10742287532340     -1.97765531957536      h
   -1.51585533860606     -2.39978914899268      1.51834204785310      h
    1.61175861621726      1.98216750264260      0.21586745528526      pd
    1.84913836347077      2.65395348844493     -4.00149062510462      cl
    1.72268593631603      1.84624508635984      4.48906220832643      cl
$end
```

transition state

```
$coord
    0.21507292889043     -1.51080972534331     -0.52273542876202      c
    2.29043643849411     -1.52794129741692     -0.48425154355876      h
   -1.81217577299793      0.53164031650845     -0.49826524483553      h
   -0.42590423185836     -1.77570999151207     -2.47874733666819      h
   -0.62903588250522     -2.82443980996449      0.83060656694016      h
    0.08660334838630      2.18787283327841      0.96632586708258      pd
    1.58131055858864      3.97617755402660     -2.62292300941741      cl
   -1.30630738699794      0.94321012042334      4.80999012921916      cl
$end
```

product

```
$coord
    0.67848976342314     -1.63831369263933     -0.43698685403933      c
    2.66413628394210     -1.82948404485982      0.14312356862163      h
   -2.76880101485043      1.58148271374544     -0.25359681537576      h
    0.42840086374137     -1.65754714579932     -2.48953652597086      h
   -0.59251287558777     -2.89607701347438      0.60154049761396      h
   -0.14215111204539      1.96997402831566      0.73957771016106      pd
    1.07825440428098      3.76631658185848     -2.98653154700182      cl
   -1.34581631290399      0.70364857285324      4.68240996599109      cl
$end
```

Reaction **10**:

reactant

```
$coord
    0.00000000000000      0.00000000000000     -0.28802062562879      c
   -0.98271913757002     -1.70211947584155     -0.94150558033999      h
   -0.98271913757002      1.70211947584155     -0.94150558033999      h
    0.00000000000000      0.00000000000000      3.11255048145588      cl
    1.96543827514005      0.00000000000000     -0.94150558033999      h
$end
```

reactant complex

```
$coord
    1.16300999340308     -2.29998314086776      0.00000000000000      c
    3.02417936918910     -3.17246788219149      0.00000000000000      h
    0.09388785385828     -2.73948429137188      1.69107806063235      h
    1.78172723207355      1.05327116291923      0.00000000000000      cl
    0.09388785385828     -2.73948429137188     -1.69107806063235      h
   -1.90593501335062      3.20762771941425      0.00000000000000      pd
    0.32845763513767      6.78081991656540      0.00000000000000      cl
   -4.57921492416934     -0.09029919309586      0.00000000000000      cl
$end
```

transition state

```
$coord
    0.86690334674465     -1.56433754290613      0.00000000000000      c
    1.83549720222632     -2.02587606158339      1.73685460190472      h
   -1.08941496921910     -2.22994256146562      0.00000000000000      h
    2.39027931264861      2.22760057123962      0.00000000000000      cl
    1.83549720222632     -2.02587606158339     -1.73685460190472      h
   -1.74961223135330      1.88883058372024      0.00000000000000      pd
   -2.03725158665485      1.86357807835488      4.22113049256974      cl
   -2.03725158665485      1.86357807835488     -4.22113049256974      cl
$end
```

product

```
$coord
    0.54992706863347     -1.71597890151863      0.00000000000000      c
    1.67360161415041     -1.78244054639882      1.71648088633685      h
   -0.97922440306348     -3.10670533258405      0.00000000000000      h
    2.03251699401924      3.83649721426504      0.00000000000000      cl
    1.67360161415041     -1.78244054639882     -1.71648088633685      h
   -1.37073006681226      1.50015540902096      0.00000000000000      pd
   -1.79239237959806      1.52370361753004      4.20417879489267      cl
   -1.79239237959806      1.52370361753004     -4.20417879489267      cl
$end
```

Reaction **12**:

reactant

```
$coord
    0.00000000000000      0.00000000000000      1.44835159317494      c
   -1.67953511749139      0.96968005219708      2.20082092875607      h
    0.00000000000000      0.00000000000000     -1.44835159317494      c
    1.67953511749139      0.96968005219708      2.20082092875607      h
    0.00000000000000     -1.93936010439417      2.20082092875607      h
    1.67953511749139     -0.96968005219708     -2.20082092875607      h
   -1.67953511749139     -0.96968005219708     -2.20082092875607      h
    0.00000000000000      1.93936010439417     -2.20082092875607      h
$end
```

reactant complex

```
$coord
   -0.63619820205672      0.86616961305616     -0.09779996816883      c
    1.65328963730064      0.80066618686846     -0.06587401068842      h
   -0.86814577931878      2.92703221721637     -0.08789661926877      h
   -1.74189396187689      0.12581420919532     -1.70620878498053      h
   -1.30451672203806     -0.51264803645638      2.35129746103539      c
```

```
   -0.96684465751939     -2.54820621677505      2.15770209618542      h
   -0.19782763749079      0.20451875857604      3.95433284674067      h
   -3.32513185957527     -0.19524452964781      2.74285825285131      h
    2.34364374359563     -0.48835295211747     -2.92483532742154      pd
    2.36560131863852     -4.60488578422008     -1.58273753602359      cl
    2.67802412034114      3.42513653430441     -4.74083841026109      cl
$end
```

transition state

```
$coord
   -0.82005185187055      0.75374666538726     -0.22514590287524      c
    2.09137215445056      0.74507413211143     -0.21278722308816      h
   -0.83551121663035      2.82313083476840     -0.23938311026308      h
   -2.20260053078044      0.04887150017229     -1.60446598551402      h
   -1.10568935017185     -0.45444153036640      2.35752111513118      c
   -1.03895632041237     -2.51927657077724      2.25262144258342      h
    0.31836418754515      0.19117460687807      3.71965334147109      h
   -2.97543684710525      0.11479902258931      3.08769455755767      h
    2.04700039184685     -0.48683026696594     -2.84447282874586      pd
    2.29560567341070     -4.56190775870463     -1.54169503860178      cl
    2.22590370971756      3.34565936490749     -4.74954036765517      cl
$end
```

product

```
$coord
   -1.39609613522924      0.35452948128680     -0.38072412260172      c
    3.49777495740530      0.23372859090267     -0.35159767467358      h
   -1.96213906085952      2.34416817973876     -0.50145248172618      h
   -2.59842898500931     -0.90731508847667     -1.50932251848291      h
   -0.90121060433542     -0.57717800979133      2.25729175020040      c
   -0.16380124703849     -2.51560224356861      2.26305752599238      h
    0.38342852992464      0.67559253214359      3.30130711808709      h
   -2.73609787575050     -0.57457777343985      3.26667908635578      h
    1.83871073284064      0.32648384390628     -2.64405401862718      pd
    1.81326439426667     -3.98685981441796     -2.96346426134026      cl
    2.22459529378526      4.62703030171637     -2.73772040318380      cl
$end
```

Reaction **13**:
reactant (see reaction **12** in this subsection)
reactant complex (see reactin **12** in this subsection)
transition state

```
$coord
    1.69912638540017     -0.46737212449079     -0.95257702149488      c
    3.21338082440619     -0.49125418452438      0.52109137509783      h
    2.25431538600309      1.01004327893047     -2.27695846290654      h
    1.58085839999298     -2.35910885372127     -1.75685379570897      h
   -1.69912638540017      0.46737212449079     -0.95257702149488      c
   -2.25431538600309     -1.01004327893047     -2.27695846290654      h
   -3.21338082440619      0.49125418452438      0.52109137509783      h
   -1.58085839999298      2.35910885372127     -1.75685379570897      h
    0.00000000000000      0.00000000000000      2.77357616375843      pd
   -0.92488599049128     -4.18518825581856      3.07946565688819      cl
    0.92488599049128      4.18518825581856      3.07946565688819      cl
$end
```

product

```
$coord
    2.32372647611463      0.64183439394924     -1.51637262176370      c
    4.36387092138417      0.45831794321143     -1.17546599927156      h
    1.69094205675482      2.61055800145104     -1.50804568335916      h
    1.67481385482377     -0.46158757102208     -3.14044887376711      h
   -2.78944209282623     -0.04479757615874      0.57552642684900      c
   -3.06743767145323     -0.76238082320175     -1.34390124643170      h
   -3.95069322999495     -1.04581708604299      1.97577043862092      h
   -2.90835053954577      2.01244523475129      0.74677481276087      h
    0.79474025549654     -1.01708239407071      1.60832031463395      pd
    0.52379848844100     -4.87733669101673     -0.24892983433230      cl
    1.34403148080526      2.48584656814999      4.02677226606081      cl
$end
```

1.4 Ni subset

Reaction **2**:

reactant

```
$coord
    0.00000000000000      0.00000000000000      0.54751735465957      n
   -0.89451705630777      1.54934898976201     -0.18250578488651      h
   -0.89451705630777     -1.54934898976201     -0.18250578488651      h
    1.78903411261554      0.00000000000000     -0.18250578488651      h
$end
```

reactant complex

```
$coord
    0.00000000000000      0.00000000000000      0.28685590672041      n
    0.91293146331442     -1.58124367828877      0.96678990969926      h
    0.91293146331442      1.58124367828877      0.96678990969926      h
   -1.82586292662883      0.00000000000000      0.96678990969926      h
    0.00000000000000      0.00000000000000     -3.18722563581818      ni
$end
```

transition state

```
$coord
   -0.77683072762924     -0.11736151015521      0.00000000000000      n
    1.91558227453843     -0.20422405650096      0.00000000000000      h
   -1.21271505327892     -1.12048218156986      1.58442848323669      h
   -1.21271505327892     -1.12048218156986     -1.58442848323669      h
    1.28667855964864      2.56254992979589      0.00000000000000      ni
$end
```

product

```
$coord
   -1.42972865624403      0.19662534313075      0.00000000000000      n
    3.34621984337234     -0.12516398871079      0.00000000000000      h
   -1.67444508335942     -0.94873812182454      1.55585436950496      h
   -1.67444508335942     -0.94873812182454     -1.55585436950496      h
    1.43239897959054      1.82601488922911      0.00000000000000      ni
$end
```

Reaction **3**:

reactant

```
$coord
   -0.82523722550861     -1.42935280287808      0.00000000000000      c
   -0.82523722550861      1.42935280287808      0.00000000000000      c
    1.65047445101721      0.00000000000000      0.00000000000000      c
    2.76792796930105      0.00000000000000     -1.73468085727880      h
    2.76792796930105      0.00000000000000      1.73468085727880      h
   -1.38396398465053     -2.39709593726019      1.73468085727880      h
   -1.38396398465053     -2.39709593726019     -1.73468085727880      h
   -1.38396398465053      2.39709593726019      1.73468085727880      h
   -1.38396398465053      2.39709593726019     -1.73468085727880      h
$end
```

reactant complex

```
$coord
    0.00000000000000      0.00000000000000     -1.12466083689758      c
    0.00000000000000     -1.43705661354697      1.33825658448129      c
    0.00000000000000      1.43705661354697      1.33825658448129      c
   -1.73304749458467      2.40765494707779      1.89700192590539      h
    1.73304749458467      2.40765494707779      1.89700192590539      h
    1.91260016552756      0.00000000000000     -2.20219643590297      h
   -1.91260016552756      0.00000000000000     -2.20219643590297      h
    1.73304749458467     -2.40765494707779      1.89700192590539      h
   -1.73304749458467     -2.40765494707779      1.89700192590539      h
    0.00000000000000      0.00000000000000     -4.73546716388066      ni
$end
```

transition state

```
$coord
   -1.00760009378983     -0.81178880828233      0.31051041725687      c
    1.84084478024066     -0.37161291383612      0.17664148365256      c
    0.15429666611655      1.90023719526415      0.25407492659644      c
    0.00582470247272      2.98112339989403      2.00614735703188      h
   -0.04256280115501      3.01599448391286     -1.46713589244392      h
   -1.51051620208137     -2.73785806033171     -1.53086677391075      h
   -1.68264462198599     -1.45268549041152      2.16240526206990      h
    2.78943446822648     -0.78922456598505     -1.60651569116670      h
    2.92887008364752     -0.84322217141034      1.86551744623600      h
   -3.47594698169171     -0.89096306881394     -2.17077853532226      ni
$end
```

product

```
$coord
   -1.25576975622739     -0.51735056176003      0.00000000000000      c
    1.20686962950331     -0.51883476343103     -1.43549534124936      c
    1.20686962950331     -0.51883476343103      1.43549534124936      c
    1.74494599301157     -2.24908491674311      2.42751434195099      h
    1.77870430819706      1.22029085338201      2.38238945361745      h
   -2.10039016723960      4.03865639410389      0.00000000000000      h
   -2.37308642407264     -2.29370034689412      0.00000000000000      h
    1.77870430819706      1.22029085338201     -2.38238945361745      h
    1.74494599301157     -2.24908491674311     -2.42751434195099      h
   -3.73179351388423      1.86765216813453      0.00000000000000      ni
$end
```

Reaction **5**:

reactant

```
$coord
    0.00000000000000     -1.26008101970436      0.00000000000000      c
    0.00000000000000      1.26008101970436      0.00000000000000      c
   -1.75890496375767     -2.34107004708163      0.00000000000000      h
   -1.75890496375767      2.34107004708163      0.00000000000000      h
    1.75890496375767      2.34107004708163      0.00000000000000      h
    1.75890496375767     -2.34107004708163      0.00000000000000      h
$end
```

reactant complex

```
$coord
    0.00000000000000     -1.35234243954768     -0.23179152839963      c
    0.00000000000000      1.35234243954768     -0.23179152839963      c
   -1.74496386044381     -2.39253899439181     -0.65949703462163      h
   -1.74496386044381      2.39253899439181     -0.65949703462163      h
    1.74496386044381      2.39253899439181     -0.65949703462163      h
    1.74496386044381     -2.39253899439181     -0.65949703462163      h
    0.00000000000000      0.00000000000000      3.10157119528576      ni
$end
```

transition state

```
$coord
   -0.76328377782757     -0.34326317400863      0.36629558052469      c
    1.60895047370163     -0.63455082971896     -0.54709695740369      c
   -2.04938179364006      2.23952553024808     -0.44551993919236      h
    2.97845649503975      0.90670799107956     -0.56541453439875      h
    2.23692343211524     -2.46344928519857     -1.28339347546436      h
   -2.12436852548888     -1.90502973333584      0.17052847895925      h
   -1.88729630390011      2.20005950093436      2.30460084697521      ni
$end
```

product

```
$coord
   -0.61026386734441     -0.48645726207152     -0.33745268742937      c
    1.92252074078696     -0.59489748960649     -0.18999345004730      c
   -3.28826894680356      2.89499659551492      0.46961739602570      h
    3.01209126890532      0.49727471753469      1.18228453712715      h
    3.00386265509560     -1.82826379147408     -1.45956470934624      h
   -1.68818718973737     -1.46146350890319     -1.81881814176209      h
   -2.35175466090255      0.97881073900564      2.15392705543217      ni
$end
```

Reaction **6**:

reactant

```
$coord
    0.00000000000000      0.00000000000000      0.00000000000000      b
    1.13316583511206     -1.96270079981531      0.00000000000000      h
    1.13316583511206      1.96270079981531      0.00000000000000      h
   -2.26633167022413      0.00000000000000      0.00000000000000      h
$end
```

reactant complex

```
$coord
    0.00000000000000      0.00000000000000      0.67219950917333      b
    0.00000000000000      0.00000000000000      2.92058081488604      h
    2.18624424999189      0.00000000000000     -0.44675596475902      h
   -2.18624424999189      0.00000000000000     -0.44675596475902      h
    0.00000000000000      0.00000000000000     -2.69926839454133      ni
$end
```

transition state

```
$coord
    0.56764462013988      0.64646954077486      0.00000000000000      b
    2.81940311655741      0.64404610605847      0.00000000000000      h
   -0.81241767368125      2.54952640235860      0.00000000000000      h
   -0.26732340512957     -2.84347708088955      0.00000000000000      h
   -2.30730665788647     -0.99656496830238      0.00000000000000      ni
$end
```

product

```
$coord
    0.89952632775172      0.85509547307752      0.00000000000000      b
    3.09384758724233      1.35966248830626      0.00000000000000      h
   -1.01411002346282      2.32974879513927      0.00000000000000      h
   -1.15706573852224     -3.62160254586818      0.00000000000000      h
   -1.82219815300898     -0.92290421065486      0.00000000000000      ni
$end
```

Reaction **7**:

reactant

```
$coord
    0.00000000000000      0.00000000000000     -0.70938388323755      h
    0.00000000000000      0.00000000000000      0.70938388323755      h
$end
```

reactant complex

```
$coord
    0.99637601082228      0.00000000000000     -0.85243399641568      h
   -0.99637601082228      0.00000000000000     -0.85243399641568      h
    0.00000000000000      0.00000000000000      1.70486799283135      ni
$end
```

transition state

```
$coord
    1.09778018112369      0.00000000000000     -0.82819955550747      h
   -1.09778018112369      0.00000000000000     -0.82819955550747      h
    0.00000000000000      0.00000000000000      1.65639911101494      ni
$end
```

product

```
$coord
    1.72373291803840      0.00000000000000     -0.68800697347133      h
   -1.72373291803840      0.00000000000000     -0.68800697347133      h
    0.00000000000000      0.00000000000000      1.37601394694265      ni
$end
```

Reaction **8**:

reactant

```
$coord
    2.28956539705871     -1.32188119831910      0.00000000000000      c
    2.28956539705871      1.32188119831910      0.00000000000000      c
    0.00000000000000      2.64376239663820      0.00000000000000      c
   -2.28956539705871      1.32188119831910      0.00000000000000      c
   -2.28956539705871     -1.32188119831910      0.00000000000000      c
    0.00000000000000     -2.64376239663820      0.00000000000000      c
    4.07560621506293      2.35305234537750      0.00000000000000      h
    0.00000000000000      4.70610469075499      0.00000000000000      h
   -4.07560621506293      2.35305234537750      0.00000000000000      h
   -4.07560621506293     -2.35305234537750      0.00000000000000      h
    0.00000000000000     -4.70610469075499      0.00000000000000      h
    4.07560621506293     -2.35305234537750      0.00000000000000      h
$end
```

reactant complex

```
$coord
   -2.16577144567216     -0.23254891297467     -1.37069820206544      c
    0.20997506383824     -0.13430506636057     -2.66693103833371      c
    2.48084584044899     -0.16369863184425     -1.33598010366742      c
    2.48084584044899     -0.16369863184425      1.33598010366742      c
    0.20997506383824     -0.13430506636057      2.66693103833371      c
   -2.16577144567216     -0.23254891297467      1.37069820206544      c
    0.22199000566835     -0.13651535408838     -4.72934804126157      h
    4.26923176493660     -0.15921920350053     -2.36198738003450      h
    4.26923176493660     -0.15921920350053      2.36198738003450      h
    0.22199000566835     -0.13651535408838      4.72934804126157      h
   -3.86982416739185     -0.74850014486888      2.42594376706199      h
   -3.86982416739185     -0.74850014486888     -2.42594376706199      h
   -2.29289412365640      3.14957462727460      0.00000000000000      ni
$end
```

transition state

```
$coord
   -0.80965820625380      0.19016033336380     -2.30315258949565      c
    1.79108988756243     -0.25702800070481     -2.28924795146602      c
    3.10075550323625     -0.47402050704056      0.00000000000000      c
    1.79108988756243     -0.25702800070481      2.28924795146602      c
   -0.80965820625380      0.19016033336380      2.30315258949565      c
   -2.13807280946860      0.50616514953132      0.00000000000000      c
    2.80044547357573     -0.45353952571002     -4.07812261776697      h
    5.12996737091483     -0.83955594180495      0.00000000000000      h
    2.80044547357573     -0.45353952571002      4.07812261776697      h
   -1.82503277205343      0.32239440106682      4.09129342505189      h
   -4.78009937166969     -0.76342203317646      0.00000000000000      h
   -1.82503277205343      0.32239440106682     -4.09129342505189      h
   -5.22623945867463      1.96685891645905      0.00000000000000      ni
$end
```

product

```
$coord
   -0.64176266618921     -2.34972566309567     -0.14108791127718      c
    1.94672126659200     -2.25609530188503      0.40023122239732      c
    3.19287775858027      0.07042570121190      0.55698603823499      c
    1.85014427337686      2.31737498899056      0.17600707870932      c
   -0.73584618289532      2.25196624993932     -0.37129945590226      c
   -1.97548636305765     -0.09101357800983     -0.59090118951632      c
    2.98406200803912     -4.01064805630374      0.72277577605494      h
    5.20593937351321      0.13472823654541      0.99833847591514      h
    2.81438928495949      4.13605986038914      0.31904284863565      h
   -1.77874112473497      4.00902487959298     -0.63773702523560      h
   -6.12582897611199      0.24441522423359      0.62904392179454      h
   -1.60916195491759     -4.16854066027449     -0.22132986542649      h
   -5.12730669715421     -0.28797188133409     -1.84006991438404      ni
$end
```

Reaction **9**:

reactant

```
$coord
    0.00000000000000      0.00000000000000      0.00000000000000      c
    1.19617347266982     -1.19617347266982      1.19617347266982      h
   -1.19617347266982      1.19617347266982      1.19617347266982      h
    1.19617347266982      1.19617347266982     -1.19617347266982      h
   -1.19617347266982     -1.19617347266982     -1.19617347266982      h
$end
```

reactant complex

```
$coord
    0.00000000000000      0.00000000000000      0.60428003200926      c
    0.00000000000000      1.72375903815253      1.76522811991450      h
   -1.89962313307762      0.00000000000000     -0.53416086082685      h
    1.89962313307762      0.00000000000000     -0.53416086082685      h
    0.00000000000000     -1.72375903815253      1.76522811991450      h
    0.00000000000000      0.00000000000000     -3.06641455018456      ni
$end
```

transition state

```
$coord
    0.25093304174184     -0.49540676585565      0.26419061483036      c
    2.33228527920949     -0.49513259358197      0.25184845172864      h
   -0.08360201817897      2.26782896331387      0.29078359353943      h
   -0.43602772569517     -1.99998229449926     -1.06440735071360      h
   -0.47733792157023     -0.89474720775368      2.17276715126421      h
   -1.58625065550697      1.61743989837671     -1.91518246064906      ni
$end
```

product

```
$coord
    0.43879296568221      0.85073241283684      0.00000000000000      c
    1.58692302586500      0.61121468022320      1.71728488050077      h
   -0.77829803139291     -3.51469967266279      0.00000000000000      h
   -0.52470709895874      2.72267252043543      0.00000000000000      h
    1.58692302586500      0.61121468022320     -1.71728488050077      h
   -2.30963388706055     -1.28113462105587      0.00000000000000      ni
$end
```

Reaction **12**:

reactant

```
$coord
    0.00000000000000      0.00000000000000      1.44839479701059      c
    1.67958305934057      0.96970773143662      2.20086226262138      h
    0.00000000000000      0.00000000000000     -1.44839479701059      c
   -1.67958305934057      0.96970773143662      2.20086226262138      h
    0.00000000000000     -1.93941546287323      2.20086226262138      h
   -1.67958305934057     -0.96970773143662     -2.20086226262138      h
    1.67958305934057     -0.96970773143662     -2.20086226262138      h
    0.00000000000000      1.93941546287323     -2.20086226262138      h
$end
```

reactant complex

```
$coord
    0.35845091538663     -1.85281506405941      0.00000000000000      c
    2.43377574236988     -1.85137779993397      0.00000000000000      h
   -0.29025651616682     -2.88668341344195      1.68581516187084      h
   -0.67458608857804      0.85528517473553      0.00000000000000      c
   -0.29025651616682     -2.88668341344195     -1.68581516187084      h
   -2.75294183129176      0.93099950927902      0.00000000000000      h
   -0.06574899579215      1.86816642168539      1.88274740457215      h
   -0.06574899579215      1.86816642168539     -1.88274740457215      h
    1.34731228603126      3.95494216349192      0.00000000000000      ni
$end
```

transition state

```
$coord
    0.42395093138419      0.80621268560661      1.61565620644403      c
    2.49813032472779      0.73341570145250      1.67011091939656      h
   -0.14117736966083      2.80142502096120      1.47578518904227      h
   -0.62124295204042     -0.73815095771591     -0.60310805907850      c
   -0.30247687687733      0.05523443199071      3.41903326835838      h
   -2.70372847392552     -0.69592203703296     -0.67581103871518      h
   -0.29569332084377      0.98882468523255     -2.81629293382115      h
   -0.05802864588454     -2.78587040504992     -0.44668542760128      h
    1.20026638312041     -1.16516912544476     -3.63868812402516      ni
$end
```

product

```
$coord
    0.50179077090185      0.41414176452556      1.77367319770388      c
    2.57863989578717      0.42988629355148      1.74970183262791      h
   -0.16330657131301      2.38100933750139      1.80434430414620      h
   -0.59005484877269     -0.99974128464886     -0.47994299960270      c
   -0.10889998842709     -0.48989620316121      3.55692260387949      h
   -2.67499538213206     -0.98465031606828     -0.50902472633404      h
   -0.19478982269880      2.43181226872249     -3.65892968484266      h
    0.09466441817421     -3.00195752117367     -0.57798358186634      h
    0.55695152848041     -0.18060433924887     -3.65876094571177      ni
$end
```

Reaction **13**:

reactant (see reaction **12** in this subsection)

reactant complex

```
$coord
    0.00000000000000      1.48647106138799     -0.41249041598450      c
   -1.69764069883737      2.23973338194757     -1.33576914845319      h
    0.00000000000000      2.72619799201904      1.59654513042054      h
    0.00000000000000     -1.48647106138799     -0.41249041598450      c
    1.69764069883737      2.23973338194757     -1.33576914845319      h
   -1.69764069883737     -2.23973338194757     -1.33576914845319      h
    1.69764069883737     -2.23973338194757     -1.33576914845319      h
    0.00000000000000     -2.72619799201904      1.59654513042054      h
    0.00000000000000      0.00000000000000      2.97496716494066      ni
$end
```

transition state

```
$coord
    0.00000000000000      1.81285267397015     -0.25609817678638      c
   -1.72480296613349      2.23734656584727     -1.32002539831306      h
    0.00000000000000      3.13938412130056      1.49134527379225      h
    0.00000000000000     -1.81285267397015     -0.25609817678638      c
    1.72480296613349      2.23734656584727     -1.32002539831306      h
   -1.72480296613349     -2.23734656584727     -1.32002539831306      h
    1.72480296613349     -2.23734656584727     -1.32002539831306      h
    0.00000000000000     -3.13938412130056      1.49134527379225      h
    0.00000000000000      0.00000000000000      2.80960739924054      ni
$end
```

product

```
$coord
    0.00000000000000      2.75344004121354     -0.02917704184833      c
   -1.71391006238707      2.82040931709841     -1.20613356486355      h
    0.00000000000000      4.32718456671668      1.36668582405183      h
    0.00000000000000     -2.75344004121354     -0.02917704184833      c
    1.71391006238707      2.82040931709841     -1.20613356486355      h
   -1.71391006238707     -2.82040931709841     -1.20613356486355      h
    1.71391006238707     -2.82040931709841     -1.20613356486355      h
    0.00000000000000     -4.32718456671668      1.36668582405183      h
    0.00000000000000      0.00000000000000      2.12185939553742      ni
$end
```

2 Complete Results for all Subsets

2.1 Pd subset

Table 1: Deviation from the CCSD(T)/CBS values in kcal/mol for tested density functionals without dispersion correction.

Molecule (bond activation)	Reaction Step	CCSD(T)	SVWN	BP86	BLYP	PBE
Benzene	D_e	28.85	21.86	3.29	-4.79	4.93
(C-H)	ΔE_{forw}	23.67	-5.11	-4.43	-4.95	-3.80
	ΔE_{back}	1.63	1.17	2.16	3.33	1.74
	ΔE_{reac}	22.04	-6.28	-6.59	-8.27	-5.53
BH_3	D_e	32.14	27.08	7.94	0.10	9.57
(B-H)	ΔE_{forw}	2.32	-3.02	-2.13	-1.94	-1.79
	ΔE_{back}	-0.76	3.51	3.28	4.04	2.93
	ΔE_{reac}	3.08	-6.53	-5.41	-5.98	-4.71
C_2H_2	D_e	36.71	27.16	8.72	0.42	10.20
(C-H)	ΔE_{forw}	25.14	-0.33	-0.36	-1.16	-0.11
	ΔE_{back}	0.53	0.53	1.35	2.19	0.80
	ΔE_{reac}	24.61	-0.86	-1.71	-3.34	-0.91
C_2H_4	D_e	38.66	24.75	6.42	-1.59	7.89
(C-H)	ΔE_{forw}	33.82	-4.33	-3.71	-4.35	-3.21
	ΔE_{back}	1.41	1.17	2.08	3.28	1.66
	ΔE_{reac}	32.42	-5.50	-5.80	-7.63	-4.88
C_2H_6	D_e	10.75	17.42	1.90	-3.28	2.99
(C-C)	ΔE_{forw}	26.06	-9.57	-5.05	-2.43	-5.32
	ΔE_{back}	21.61	-0.12	4.56	7.73	3.50
	ΔE_{reac}	4.45	-9.45	-9.61	-10.16	-8.82
Cyclopropane	D_e	9.66	16.66	1.61	-3.40	2.70
(C-H)	ΔE_{forw}	10.46	-10.25	-6.50	-4.31	-6.42
	ΔE_{back}	5.14	1.58	2.74	4.10	2.21
	ΔE_{reac}	5.32	-11.83	-9.24	-8.40	-8.63
H_2O	D_e	8.95	12.87	2.45	0.72	2.97
(O-H)	ΔE_{forw}	26.57	-15.02	-9.98	-7.37	-9.93
	ΔE_{back}	9.39	-0.83	1.47	2.94	1.13
	ΔE_{reac}	17.18	-14.19	-11.45	-10.31	-11.05
C_2H_6	D_e	10.75	17.42	1.90	-3.28	2.99
(C-H)	ΔE_{forw}	12.86	-8.99	-5.92	-4.15	-5.86
	ΔE_{back}	4.98	1.91	3.05	4.43	2.50

	ΔE_{reac}	7.88	-10.90	-8.97	-8.58	-8.36
H_2 (H-H)	D_{e}	20.77	22.27	6.53	1.21	7.34
	ΔE_{forw}	3.58	-4.12	-2.37	-1.65	-2.22
	ΔE_{back}	-0.74	1.10	1.66	2.40	1.33
	ΔE_{reac}	4.32	-5.22	-4.03	-4.05	-3.55
CH_4 (C-H)	D_{e}	9.92	18.13	2.85	-2.29	3.90
	ΔE_{forw}	12.29	-8.35	-5.63	-4.00	-5.56
	ΔE_{back}	4.12	1.21	2.63	4.07	2.06
	ΔE_{reac}	8.17	-9.56	-8.26	-8.07	-7.62
NH_3 (N-H)	D_{e}	23.17	17.96	3.98	0.53	4.38
	ΔE_{forw}	32.04	-9.55	-6.59	-4.59	-6.80
	ΔE_{back}	7.85	0.77	2.50	3.83	2.06
	ΔE_{reac}	24.18	-10.31	-9.08	-8.40	-8.85
CH_3Cl (OxIn) (C-Cl)	D_{e}	13.96	18.51	4.93	0.73	5.67
	ΔE_{forw}	15.77	-4.66	-3.37	-2.95	-3.53
	ΔE_{back}	34.12	-0.10	0.19	0.72	-0.20
	ΔE_{reac}	-18.35	-4.56	-3.57	-3.67	-3.33
CH_3Cl (S_N2) (C-Cl)	D_{e}	8.49	18.15	2.64	-2.58	3.80
	ΔE_{forw}	47.52	-6.57	-14.95	-19.89	-14.20
	ΔE_{back}	71.35	-1.66	-9.11	-12.91	-9.01
	ΔE_{reac}	-23.83	-4.91	-5.84	-6.98	-5.19
		MAD	8.96	4.86	4.59	4.82
		MD	1.55	-1.67	-2.79	-1.31
		RMS	11.76	5.77	5.81	5.75

Table 2: Deviation from the CCSD(T)/CBS values in kcal/mol for tested density functionals without dispersion correction.

Molecule (bond activation)	Reaction Step	CCSD(T)	B97-d	TPSS	oTPSS	M06-L
Benzene (C-H)	D_{e}	28.85	-6.51	3.53	0.17	0.49
	ΔE_{forw}	23.67	-4.29	-2.21	-2.71	1.70
	ΔE_{back}	1.63	2.22	2.42	2.32	0.12
	ΔE_{reac}	22.04	-6.51	-4.63	-5.03	1.58
BH_3 (B-H)	D_{e}	32.14	-1.89	5.32	4.69	-0.55
	ΔE_{forw}	2.32	-1.62	-1.18	-0.45	0.97
	ΔE_{back}	-0.76	3.10	2.46	1.77	-0.05
	ΔE_{reac}	3.08	-4.71	-3.65	-2.23	1.02
C_2H_2 (C-H)	D_{e}	36.71	-1.43	9.06	4.13	4.02
	ΔE_{forw}	25.14	-1.25	1.60	-1.18	1.95

	ΔE_{back}	0.53	0.87	1.98	1.62	-1.79
	ΔE_{reac}	24.61	-2.12	-0.38	-2.80	3.74
C_2H_4	D_{e}	38.66	-3.50	6.53	3.03	2.72
(C-H)	ΔE_{forw}	33.82	-3.81	-1.46	-2.37	1.89
	ΔE_{back}	1.41	2.17	2.28	2.24	0.19
	ΔE_{reac}	32.42	-5.99	-3.75	-4.61	1.69
C_2H_6	D_{e}	10.75	-5.37	0.71	-0.68	-0.69
(C-C)	ΔE_{forw}	26.06	-1.36	-5.17	-3.71	-0.36
	ΔE_{back}	21.61	6.09	5.65	4.95	1.52
	ΔE_{reac}	4.45	-7.45	-10.81	-8.66	-1.88
Cyclopropane	D_{e}	9.66	-5.46	0.61	-0.82	-0.69
(C-H)	ΔE_{forw}	10.46	-3.82	-5.25	-3.85	0.36
	ΔE_{back}	5.14	2.80	3.35	3.20	0.88
	ΔE_{reac}	5.32	-6.62	-8.60	-7.05	-0.52
H_2O	D_{e}	8.95	-2.28	2.31	-0.43	1.66
(O-H)	ΔE_{forw}	26.57	-5.73	-10.92	-9.45	-2.01
	ΔE_{back}	9.39	1.28	3.26	3.65	1.23
	ΔE_{reac}	17.18	-7.01	-14.18	-13.10	-3.24
C_2H_6	D_{e}	10.75	-5.37	0.71	-0.68	-0.69
(C-H)	ΔE_{forw}	12.86	-3.87	-4.70	-3.69	0.22
	ΔE_{back}	4.98	2.91	3.74	3.22	0.60
	ΔE_{reac}	7.88	-6.78	-8.45	-6.91	-0.38
H_2	D_{e}	20.77	-0.51	3.17	4.09	2.69
(H-H)	ΔE_{forw}	3.58	-1.29	-1.77	-1.63	0.77
	ΔE_{back}	-0.74	1.46	1.97	1.37	-0.26
	ΔE_{reac}	4.32	-2.74	-3.73	-3.01	1.03
CH_4	D_{e}	9.92	-4.39	1.63	0.35	-0.02
(C-H)	ΔE_{forw}	12.29	-3.74	-4.54	-3.49	0.56
	ΔE_{back}	4.12	2.55	3.45	2.97	0.26
	ΔE_{reac}	8.17	-6.29	-7.99	-6.46	0.30
NH_3	D_{e}	23.17	-2.61	3.29	-0.53	-0.26
(N-H)	ΔE_{forw}	32.04	-3.93	-6.89	-7.04	-1.33
	ΔE_{back}	7.85	2.33	3.68	3.67	1.52
	ΔE_{reac}	24.18	-6.25	-10.55	-10.69	-2.84
CH_3Cl	D_{e}	13.96	-1.55	4.31	0.94	1.75
(OxIn)	ΔE_{forw}	15.77	-1.73	-4.16	-4.72	-2.41
(C-Cl)	ΔE_{back}	34.12	-0.76	1.67	3.36	-1.07
	ΔE_{reac}	-18.35	-0.97	-5.83	-8.08	-1.34
CH_3Cl	D_{e}	8.49	-4.61	1.31	0.15	0.31
(S_N2)	ΔE_{forw}	47.52	-17.53	-16.20	-18.79	-11.55

(C-Cl)	$\Delta E_{\rm back}$	71.35	-13.51	-7.39	-9.93	-8.77
	$\Delta E_{\rm reac}$	-23.83	-4.02	-8.81	-8.86	-2.78
		MAD	4.02	4.68	4.14	1.60
		MD	-2.95	-1.60	-2.15	-0.15
		RMS	5.04	5.82	5.50	2.55

Table 3: Deviation from the CCSD(T)/CBS values in kcal/mol for tested density functionals without dispersion correction.

Molecule (bond activation)	Reaction Step	CCSD(T)	B3LYP	BHLYP	PBE0	PW6B95
Benzene	$D_{\rm e}$	28.85	-9.22	-17.07	-2.73	-5.43
(C-H)	$\Delta E_{\rm forw}$	23.67	-1.46	3.59	0.28	1.65
	$\Delta E_{\rm back}$	1.63	1.84	0.27	-0.01	0.03
	$\Delta E_{\rm reac}$	22.04	-3.30	3.32	0.29	1.63
BH_3	$D_{\rm e}$	32.14	-6.10	-16.52	-0.28	-3.52
(B-H)	$\Delta E_{\rm forw}$	2.32	-0.11	2.63	0.46	1.11
	$\Delta E_{\rm back}$	-0.76	1.67	-1.50	-0.02	-0.21
	$\Delta E_{\rm reac}$	3.08	-1.78	4.13	0.48	1.32
C_2H_2	$D_{\rm e}$	36.71	-5.10	-14.83	1.37	-2.60
(C-H)	$\Delta E_{\rm forw}$	25.14	-0.33	0.60	0.82	0.99
	$\Delta E_{\rm back}$	0.53	0.27	-1.78	-1.38	-1.68
	$\Delta E_{\rm reac}$	24.61	-0.60	2.38	2.21	2.67
C_2H_4	$D_{\rm e}$	38.66	-6.42	-15.18	-0.09	-3.73
(C-H)	$\Delta E_{\rm forw}$	33.82	-1.03	3.38	0.77	1.36
	$\Delta E_{\rm back}$	1.41	1.89	0.46	0.03	0.10
	$\Delta E_{\rm reac}$	32.42	-2.93	2.91	0.73	1.25
C_2H_6	$D_{\rm e}$	10.75	-5.94	-10.68	-2.15	-3.88
(C-C)	$\Delta E_{\rm forw}$	26.06	2.00	8.95	0.54	0.93
	$\Delta E_{\rm back}$	21.61	4.49	0.61	0.22	-0.49
	$\Delta E_{\rm reac}$	4.45	-2.49	8.35	0.31	1.42
Cyclopropane	$D_{\rm e}$	9.66	-6.12	-10.77	-2.42	-3.99
(C-H)	$\Delta E_{\rm forw}$	10.46	0.73	8.69	-0.07	2.21
	$\Delta E_{\rm back}$	5.14	2.52	0.77	0.37	0.42
	$\Delta E_{\rm reac}$	5.32	-1.79	7.92	-0.44	1.79
H_2O	$D_{\rm e}$	8.95	-1.85	-5.27	-1.31	-2.00
(O-H)	$\Delta E_{\rm forw}$	26.57	-0.30	10.41	-0.86	0.91
	$\Delta E_{\rm back}$	9.39	2.39	2.57	0.68	0.70
	$\Delta E_{\rm reac}$	17.18	-2.69	7.84	-1.54	0.21
C_2H_6	$D_{\rm e}$	10.75	-5.94	-10.68	-2.15	-3.88

(C-H)	ΔE_{forw}	12.86	0.19	7.00	-0.40	1.44
	ΔE_{back}	4.98	2.24	-0.50	-0.07	-0.16
	ΔE_{reac}	7.88	-2.05	7.51	-0.33	1.60
H_2	D_{e}	20.77	-2.41	-9.16	1.16	-2.62
(H-H)	ΔE_{forw}	3.58	0.26	3.30	0.20	0.91
	ΔE_{back}	-0.74	1.17	-0.20	-0.10	-0.04
	ΔE_{reac}	4.32	-0.91	3.50	0.31	0.95
CH_4	D_{e}	9.92	-4.90	-9.62	-1.12	-2.93
(C-H)	ΔE_{forw}	12.29	0.17	6.74	-0.36	1.46
	ΔE_{back}	4.12	2.08	-0.29	-0.19	-0.25
	ΔE_{reac}	8.17	-1.91	7.03	-0.17	1.71
NH_3	D_{e}	23.17	-3.24	-9.04	-1.74	-3.72
(N-H)	ΔE_{forw}	32.04	0.22	7.82	-0.70	0.41
	ΔE_{back}	7.85	2.39	1.18	0.46	0.38
	ΔE_{reac}	24.18	-2.15	6.65	-1.16	0.04
CH_3Cl	D_{e}	13.96	-3.01	-8.50	-0.50	-2.44
(OxIn)	ΔE_{forw}	15.77	-0.10	3.79	0.12	-0.87
(C-Cl)	ΔE_{back}	34.12	-0.27	-1.96	-1.22	-2.61
	ΔE_{reac}	-18.35	0.17	5.75	1.33	1.74
CH_3Cl	D_{e}	8.49	-5.43	-10.36	-1.57	-3.41
(S_N2)	ΔE_{forw}	47.52	-10.18	-0.74	-3.77	-3.80
(C-Cl)	ΔE_{back}	71.35	-7.94	-4.64	-4.04	-4.59
	ΔE_{reac}	-23.83	-2.25	3.90	0.27	0.78
		MAD	2.67	5.83	0.89	1.75
		MD	-1.65	-0.29	-0.37	-0.51
		RMS	3.59	7.32	1.28	2.21

Table 4: Deviation from the CCSD(T)/CBS values in kcal/mol for tested density functionals without dispersion correction.

Molecule (bond activation)	Reaction Step	CCSD(T)	BMK	TPSSh	M05	M05-2X
Benzene	D_{e}	28.85	-2.83	0.33	-12.09	-9.57
(C-H)	ΔE_{forw}	23.67	-1.26	-0.73	5.88	3.02
	ΔE_{back}	1.63	6.19	1.72	-3.93	-0.40
	ΔE_{reac}	22.04	-7.44	-2.45	9.81	3.42
BH_3	D_{e}	32.14	-2.76	1.65	-15.52	-9.95
(B-H)	ΔE_{forw}	2.32	-1.37	-0.35	2.95	0.75
	ΔE_{back}	-0.76	1.77	1.34	-3.54	-1.24
	ΔE_{reac}	3.08	-3.13	-1.68	6.49	1.99

C_2H_2 (C-H)	D_e	36.71	4.10	5.35	-10.06	-9.76
	ΔE_{forw}	25.14	3.92	1.80	2.17	-0.05
	ΔE_{back}	0.53	7.20	1.05	-7.78	-1.36
	ΔE_{reac}	24.61	-3.28	0.75	9.95	1.31
C_2H_4 (C-H)	D_e	38.66	1.79	3.20	-10.31	-9.75
	ΔE_{forw}	33.82	1.58	-0.05	5.52	1.65
	ΔE_{back}	1.41	6.20	1.64	-3.82	-0.20
	ΔE_{reac}	32.42	-4.63	-1.70	9.32	1.85
C_2H_6 (C-C)	D_e	10.75	-3.00	-1.30	-9.22	-6.42
	ΔE_{forw}	26.06	0.50	-2.73	3.08	4.82
	ΔE_{back}	21.61	12.49	4.18	-6.93	-1.24
	ΔE_{reac}	4.45	-11.99	-6.91	10.01	6.06
Cyclopropane (C-H)	D_e	9.66	-3.25	-1.41	-9.04	-6.58
	ΔE_{forw}	10.46	-1.36	-2.73	6.89	5.16
	ΔE_{back}	5.14	8.82	2.60	-4.57	-0.18
	ΔE_{reac}	5.32	-10.19	-5.33	11.46	5.34
H_2O (O-H)	D_e	8.95	0.82	0.44	-5.58	-2.22
	ΔE_{forw}	26.57	-2.26	-7.09	8.66	5.83
	ΔE_{back}	9.39	12.43	3.01	-3.48	-0.01
	ΔE_{reac}	17.18	-14.69	-10.10	12.14	5.84
C_2H_6 (C-H)	D_e	10.75	-3.00	-1.30	-9.22	-6.42
	ΔE_{forw}	12.86	-0.03	-2.55	5.51	4.04
	ΔE_{back}	4.98	9.31	2.69	-5.61	-1.20
	ΔE_{reac}	7.88	-9.34	-5.24	11.12	5.24
H_2 (H-H)	D_e	20.77	-2.34	1.03	-10.71	-7.54
	ΔE_{forw}	3.58	-2.59	-0.85	4.37	0.59
	ΔE_{back}	-0.74	5.52	1.38	-3.66	-0.37
	ΔE_{reac}	4.32	-8.11	-2.23	8.03	0.96
CH_4 (C-H)	D_e	9.92	-2.06	-0.33	-8.24	-5.74
	ΔE_{forw}	12.29	-0.33	-2.48	5.45	3.98
	ΔE_{back}	4.12	9.22	2.50	-5.70	-1.07
	ΔE_{reac}	8.17	-9.55	-4.98	11.15	5.05
NH_3 (N-H)	D_e	23.17	0.83	0.70	-9.79	-6.28
	ΔE_{forw}	32.04	-0.25	-4.39	5.15	3.58
	ΔE_{back}	7.85	10.78	3.00	-4.22	-0.84
	ΔE_{reac}	24.18	-11.02	-7.38	9.38	4.43
CH_3Cl (OxIn) (C-Cl)	D_e	13.96	-1.14	1.71	-7.79	-4.86
	ΔE_{forw}	15.77	-0.35	-2.64	1.10	0.89
	ΔE_{back}	34.12	14.26	1.20	-8.48	-3.57

	ΔE_{reac}	-18.35	-14.62	-3.83	9.57	4.46
CH_3Cl (S_N2) (C-Cl)	D_{e}	8.49	-3.14	-0.76	-8.52	-6.29
	ΔE_{forw}	47.52	-12.05	-11.79	-2.30	0.60
	ΔE_{back}	71.35	4.55	-5.50	-11.14	-2.44
	ΔE_{reac}	-23.83	-16.60	-6.29	8.84	3.04
		MAD	5.62	2.89	7.41	3.64
		MD	-0.92	-1.23	-0.33	-0.42
		RMS	7.27	3.81	8.04	4.59

Table 5: Deviation from the CCSD(T)/CBS values in kcal/mol for tested density functionals without dispersion correction.

Molecule (bond activation)	Reaction Step	CCSD(T)	M06	M06-2X	M06-HF	1DH-BLYP
Benzene (C-H)	D_{e}	28.85	-6.39	-14.35	-15.12	0.50
	ΔE_{forw}	23.67	5.19	4.80	1.89	0.11
	ΔE_{back}	1.63	-3.40	-2.90	-1.14	-0.54
	ΔE_{reac}	22.04	8.59	7.70	3.02	0.65
BH_3 (B-H)	D_{e}	32.14	-10.88	-16.00	-13.86	-0.70
	ΔE_{forw}	2.32	2.57	2.58	-0.12	-0.56
	ΔE_{back}	-0.76	-2.55	-3.09	-3.08	0.83
	ΔE_{reac}	3.08	5.12	5.67	2.97	-1.39
C_2H_2 (C-H)	D_{e}	36.71	-7.50	-16.18	-17.01	2.06
	ΔE_{forw}	25.14	0.81	-1.17	-2.61	1.15
	ΔE_{back}	0.53	-6.57	-5.14	0.03	-1.68
	ΔE_{reac}	24.61	7.38	3.96	-2.64	2.83
C_2H_4 (C-H)	D_{e}	38.66	-6.54	-15.31	-16.24	1.71
	ΔE_{forw}	33.82	3.60	2.57	-0.15	0.69
	ΔE_{back}	1.41	-3.12	-2.66	-1.11	-0.70
	ΔE_{reac}	32.42	6.71	5.22	0.96	1.38
C_2H_6 (C-C)	D_{e}	10.75	-3.87	-9.41	-11.29	-1.29
	ΔE_{forw}	26.06	1.91	6.49	5.39	-1.11
	ΔE_{back}	21.61	-6.76	-5.69	-1.92	1.19
	ΔE_{reac}	4.45	8.67	12.19	7.31	-2.30
Cyclopropane (C-H)	D_{e}	9.66	-4.22	-9.34	-11.35	-1.62
	ΔE_{forw}	10.46	5.57	8.56	4.98	-1.88
	ΔE_{back}	5.14	-3.99	-3.22	-1.22	-0.30
	ΔE_{reac}	5.32	9.57	11.78	6.19	-1.58
H_2O (O-H)	D_{e}	8.95	-1.92	-3.70	-5.31	0.14
	ΔE_{forw}	26.57	7.67	11.09	6.97	-2.01

	ΔE_{back}	9.39	-3.69	-3.60	-0.98	-1.94
	ΔE_{reac}	17.18	11.36	14.69	7.95	-0.07
C_2H_6 (C-H)	D_{e}	10.75	-3.87	-9.41	-11.29	-1.29
	ΔE_{forw}	12.86	4.44	6.75	3.63	-1.46
	ΔE_{back}	4.98	-4.90	-4.69	-2.63	-0.09
	ΔE_{reac}	7.88	9.33	11.44	6.26	-1.37
H_2 (H-H)	D_{e}	20.77	-5.39	-13.17	-13.66	1.02
	ΔE_{forw}	3.58	3.99	2.45	-1.19	0.20
	ΔE_{back}	-0.74	-2.91	-2.66	-0.71	-0.80
	ΔE_{reac}	4.32	6.89	5.11	-0.48	1.00
CH_4 (C-H)	D_{e}	9.92	-3.19	-8.78	-10.67	-0.67
	ΔE_{forw}	12.29	4.87	6.62	3.13	-1.17
	ΔE_{back}	4.12	-4.93	-4.57	-2.14	-0.37
	ΔE_{reac}	8.17	9.81	11.19	5.27	-0.80
NH_3 (N-H)	D_{e}	23.17	-4.01	-10.35	-11.52	0.52
	ΔE_{forw}	32.04	5.13	5.90	2.98	-1.14
	ΔE_{back}	7.85	-4.01	-4.13	-2.33	-1.33
	ΔE_{reac}	24.18	9.15	10.05	5.32	0.20
CH_3Cl (OxIn) (C-Cl)	D_{e}	13.96	-2.74	-8.69	-9.71	0.81
	ΔE_{forw}	15.77	-0.86	1.49	2.85	-0.03
	ΔE_{back}	34.12	-10.15	-8.54	-4.04	-1.24
	ΔE_{reac}	-18.35	9.29	10.03	6.90	1.20
CH_3Cl (S_N2) (C-Cl)	D_{e}	8.49	-3.39	-9.17	-11.23	-0.97
	ΔE_{forw}	47.52	0.61	1.54	1.03	-0.23
	ΔE_{back}	71.35	-8.05	-8.02	-4.36	0.34
	ΔE_{reac}	-23.83	8.66	9.56	5.38	-0.57
		MAD	5.51	7.37	5.41	0.99
		MD	0.52	-0.47	-1.94	-0.28
		RMS	6.15	8.42	7.10	1.18

Table 6: Deviation from the CCSD(T)/CBS values in kcal/mol for tested density functionals without dispersion correction.

Molecule (bond activation)	Reaction Step	CCSD(T)	1DH-PBE	PBE0-2	PBE0-DH	PTPSS	mPW2PLYP
Benzene (C-H)	D_{e}	28.85	5.71	2.62	-2.92	-0.88	-3.76
	ΔE_{forw}	23.67	0.68	2.44	2.36	0.11	1.05
	ΔE_{back}	1.63	-1.63	-2.58	-1.03	0.96	0.01
	ΔE_{reac}	22.04	2.30	5.02	3.39	-0.86	1.04
BH_3	D_{e}	32.14	4.13	0.00	-2.68	0.11	-4.25

(B-H)	ΔE_{forw}	2.32	-0.58	0.38	1.14	0.07	0.30
	ΔE_{back}	-0.76	0.11	-1.25	-1.18	0.91	0.51
	ΔE_{reac}	3.08	-0.69	1.64	2.32	-0.85	-0.21
C_2H_2	D_{e}	36.71	7.27	3.60	-0.37	1.37	-2.21
(C-H)	ΔE_{forw}	25.14	1.76	2.12	1.45	1.34	0.74
	ΔE_{back}	0.53	-2.52	-3.61	-2.38	0.21	-1.51
	ΔE_{reac}	24.61	4.28	5.74	3.84	1.13	2.25
C_2H_4	D_{e}	38.66	6.80	3.47	-1.12	0.67	-2.51
(C-H)	ΔE_{forw}	33.82	1.35	3.02	2.65	0.55	1.15
	ΔE_{back}	1.41	-1.81	-2.69	-1.01	0.89	-0.04
	ΔE_{reac}	32.42	3.16	5.70	3.66	-0.35	1.18
C_2H_6	D_{e}	10.75	1.60	-0.45	-2.81	-1.44	-3.08
(C-C)	ΔE_{forw}	26.06	-2.86	-0.20	2.03	-0.31	1.46
	ΔE_{back}	21.61	-1.18	-2.77	-1.69	2.99	1.27
	ΔE_{reac}	4.45	-1.68	2.57	3.72	-3.30	0.20
Cyclopropane	D_{e}	9.66	1.21	-0.78	-3.08	-1.77	-3.34
(C-H)	ΔE_{forw}	10.46	-3.44	-0.65	1.81	-0.87	1.13
	ΔE_{back}	5.14	-1.59	-2.60	-0.87	1.56	0.32
	ΔE_{reac}	5.32	-1.85	1.94	2.69	-2.43	0.81
H_2O	D_{e}	8.95	0.72	-0.85	-2.11	0.62	-0.55
(O-H)	ΔE_{forw}	26.57	-3.60	0.50	2.17	-2.95	1.00
	ΔE_{back}	9.39	-3.28	-3.87	-0.25	1.28	-0.30
	ΔE_{reac}	17.18	-0.31	4.37	2.42	-4.23	1.30
C_2H_6	D_{e}	10.75	1.60	-0.45	-2.81	-1.44	-3.08
(C-H)	ΔE_{forw}	12.86	-2.72	-0.35	1.39	-0.73	0.90
	ΔE_{back}	4.98	-1.34	-2.65	-1.49	1.69	0.09
	ΔE_{reac}	7.88	-1.37	2.31	2.88	-2.42	0.82
H_2	D_{e}	20.77	4.01	1.34	-0.53	1.18	-1.29
(H-H)	ΔE_{forw}	3.58	-0.24	0.81	1.17	-0.27	1.06
	ΔE_{back}	-0.74	-1.51	-2.22	-1.02	0.53	-0.38
	ΔE_{reac}	4.32	1.26	3.04	2.19	-0.80	1.44
CH_4	D_{e}	9.92	2.18	0.14	-1.91	-0.73	-2.37
(C-H)	ΔE_{forw}	12.29	-2.34	-0.06	1.46	-0.51	1.05
	ΔE_{back}	4.12	-1.64	-2.81	-1.51	1.50	-0.07
	ΔE_{reac}	8.17	-0.70	2.74	2.97	-2.02	1.13
NH_3	D_{e}	23.17	2.08	-0.31	-2.89	0.08	-1.51
(N-H)	ΔE_{forw}	32.04	-2.57	0.27	1.47	-1.69	0.99
	ΔE_{back}	7.85	-2.63	-3.67	-0.74	1.45	-0.20
	ΔE_{reac}	24.18	0.07	3.95	2.22	-3.13	1.20
CH_3Cl	D_{e}	13.96	2.95	0.72	-1.55	0.13	-0.91

(OxIn)	ΔE_{forw}	15.77	-0.25	1.46	1.34	-1.02	0.36
(C-Cl)	ΔE_{back}	34.12	-1.66	-2.45	-1.92	1.00	-1.77
	ΔE_{reac}	-18.35	1.41	3.92	3.26	-2.03	2.13
CH_3Cl	D_{e}	8.49	1.97	-0.15	-2.33	-1.15	-2.71
(S_N2)	ΔE_{forw}	47.52	3.15	6.01	1.30	-5.75	-2.29
(C-Cl)	ΔE_{back}	71.35	2.71	2.96	-1.19	-2.45	-2.63
	ΔE_{reac}	-23.83	0.44	3.05	2.49	-3.30	0.34
		MAD	2.13	2.22	1.98	1.38	1.31
		MD	0.36	0.78	0.32	-0.53	-0.26
		RMS	2.63	2.73	2.17	1.79	1.66

Table 7: Deviation from the CCSD(T)/CBS values in kcal/mol for tested density functionals without dispersion correction.

Molecule (bond activation)	Reaction Step	CCSD(T)	B2GPPLYP	B2PLYP	PWPB95	DSD-BLYP
Benzene	D_{e}	28.85	-2.53	-2.99	-3.17	-1.59
(C-H)	ΔE_{forw}	23.67	1.01	-0.06	1.29	0.67
	ΔE_{back}	1.63	-0.51	0.41	0.02	-0.65
	ΔE_{reac}	22.04	1.52	-0.47	1.28	1.32
BH_3	D_{e}	32.14	-3.66	-2.97	-2.39	-2.93
(B-H)	ΔE_{forw}	2.32	0.10	-0.28	0.63	-0.15
	ΔE_{back}	-0.76	0.28	1.10	0.03	0.39
	ΔE_{reac}	3.08	-0.18	-1.38	0.60	-0.53
C_2H_2	D_{e}	36.71	-1.04	-0.89	-1.31	-0.17
(C-H)	ΔE_{forw}	25.14	1.13	0.69	1.23	1.23
	ΔE_{back}	0.53	-1.80	-0.88	-1.04	-1.78
	ΔE_{reac}	24.61	2.93	1.57	2.27	3.02
C_2H_4	D_{e}	38.66	-1.34	-1.46	-1.99	-0.45
(C-H)	ΔE_{forw}	33.82	1.38	0.30	1.23	1.18
	ΔE_{back}	1.41	-0.59	0.30	0.03	-0.76
	ΔE_{reac}	32.42	1.96	-0.01	1.19	1.93
C_2H_6	D_{e}	10.75	-2.94	-3.00	-3.01	-2.63
(C-C)	ΔE_{forw}	26.06	0.86	0.21	0.41	0.25
	ΔE_{back}	21.61	0.84	2.24	0.24	1.08
	ΔE_{reac}	4.45	0.03	-2.04	0.17	-0.82
Cyclopropane	D_{e}	9.66	-3.24	-3.28	-3.26	-2.94
(C-H)	ΔE_{forw}	10.46	0.23	-0.55	0.78	-0.65
	ΔE_{back}	5.14	-0.23	0.76	0.35	-0.37
	ΔE_{reac}	5.32	0.46	-1.31	0.43	-0.28

H_2O	D_e	8.95	-0.87	-0.54	-1.34	-0.73
(O-H)	ΔE_{forw}	26.57	0.56	-1.02	-0.35	-0.35
	ΔE_{back}	9.39	-1.19	-0.07	0.24	-1.76
	ΔE_{reac}	17.18	1.75	-0.95	-0.59	1.41
C_2H_6	D_e	10.75	-2.94	-3.00	-3.01	-2.63
(C-H)	ΔE_{forw}	12.86	0.27	-0.48	0.53	-0.40
	ΔE_{back}	4.98	-0.32	0.74	0.17	-0.26
	ΔE_{reac}	7.88	0.59	-1.21	0.37	-0.15
H_2	D_e	20.77	-0.91	-0.54	-1.73	-0.52
(H-H)	ΔE_{forw}	3.58	0.86	0.42	0.43	0.56
	ΔE_{back}	-0.74	-0.78	-0.09	-0.07	-0.86
	ΔE_{reac}	4.32	1.64	0.50	0.51	1.42
CH_4	D_e	9.92	-2.27	-2.27	-2.22	-2.00
(C-H)	ΔE_{forw}	12.29	0.46	-0.29	0.65	-0.18
	ΔE_{back}	4.12	-0.50	0.52	0.07	-0.48
	ΔE_{reac}	8.17	0.97	-0.81	0.59	0.30
NH_3	D_e	23.17	-1.26	-0.94	-2.43	-0.83
(N-H)	ΔE_{forw}	32.04	0.72	-0.33	-0.34	0.02
	ΔE_{back}	7.85	-0.98	0.23	0.22	-1.35
	ΔE_{reac}	24.18	1.71	-0.55	-0.55	1.38
CH_3Cl	D_e	13.96	-0.86	-0.65	-1.75	-0.58
(OxIn)	ΔE_{forw}	15.77	0.82	-0.10	-0.58	0.74
(C-Cl)	ΔE_{back}	34.12	-1.43	-0.93	-1.56	-1.19
	ΔE_{reac}	-18.35	2.25	0.82	0.97	1.93
CH_3Cl	D_e	8.49	-2.64	-2.63	-2.66	-2.35
(S_N2)	ΔE_{forw}	47.52	0.13	-3.91	-2.70	0.38
(C-Cl)	ΔE_{back}	71.35	-0.35	-2.77	-2.77	0.21
	ΔE_{reac}	-23.83	0.48	-1.15	0.07	0.17
		MAD	1.18	1.11	1.11	1.02
		MD	-0.18	-0.69	-0.46	-0.26
		RMS	1.48	1.48	1.48	1.31

Table 8: Deviation from the CCSD(T)/CBS values in kcal/mol for tested wave function methods.

Molecule (bond activation)	Reaction Step	CCSD(T)	SOS-MP2	SCS-MP2	MP2	HF
Benzene	D_e	28.85	-3.65	0.87	9.92	-38.14
(C-H)	ΔE_{forw}	23.67	0.20	0.16	0.08	9.56

	ΔE_{back}	1.63	-2.04	-3.19	-5.49	0.63
	ΔE_{reac}	22.04	2.24	3.35	5.58	8.93
BH_3	D_{e}	32.14	-6.48	-2.72	4.78	-39.85
(B-H)	ΔE_{forw}	2.32	-0.27	-0.67	-1.49	6.33
	ΔE_{back}	-0.76	-0.57	-0.64	-0.76	-5.20
	ΔE_{reac}	3.08	0.31	-0.04	-0.72	11.53
C_2H_2	D_{e}	36.71	-2.69	1.75	10.63	-39.26
(C-H)	ΔE_{forw}	25.14	1.88	2.10	2.56	0.19
	ΔE_{back}	0.53	-1.98	-3.30	-5.96	-1.46
ΔE_{reac}	24.61	3.85	5.41	8.52	1.65	
C_2H_4	D_{e}	38.66	-2.73	1.69	10.53	-38.14
(C-H)	ΔE_{forw}	33.82	1.39	1.47	1.64	7.61
	ΔE_{back}	1.41	-2.11	-3.30	-5.69	1.04
	ΔE_{reac}	32.42	3.48	4.76	7.32	6.57
C_2H_6	D_{e}	10.75	-5.20	-2.56	2.73	-25.04
(C-C)	ΔE_{forw}	26.06	1.54	-0.69	-5.17	21.09
	ΔE_{back}	21.61	1.67	-0.01	-3.36	-0.87
	ΔE_{reac}	4.45	-0.12	-0.68	-1.81	21.96
Cyclopropane	D_{e}	9.66	-5.47	-2.89	2.28	-24.80
(C-H)	ΔE_{forw}	10.46	-15.02	-16.80	-20.36	7.80
	ΔE_{back}	5.14	-15.36	-16.67	-19.29	-12.44
	ΔE_{reac}	5.32	0.34	-0.13	-1.07	20.24
H_2O	D_{e}	8.95	-2.66	-1.38	1.17	-14.82
(O-H)	ΔE_{forw}	26.57	-0.45	-1.92	-4.86	27.92
	ΔE_{back}	9.39	-4.99	-7.02	-11.09	8.12
	ΔE_{reac}	17.18	4.54	5.10	6.23	19.80
C_2H_6	D_{e}	10.75	-5.20	-2.56	2.73	-25.04
(C-H)	ΔE_{forw}	12.86	-0.55	-2.05	-5.07	17.79
	ΔE_{back}	4.98	-0.62	-1.87	-4.35	-1.92
	ΔE_{reac}	7.88	0.08	-0.19	-0.71	19.71
H_2	D_{e}	20.77	-3.41	-0.53	5.22	-26.50
(H-H)	ΔE_{forw}	3.58	0.03	-0.13	-0.47	7.66
	ΔE_{back}	-0.74	-1.45	-2.37	-4.21	-0.51
	ΔE_{reac}	4.32	1.49	2.24	3.74	8.17
CH_4	D_{e}	9.92	-4.71	-2.12	3.04	-23.94
(C-H)	ΔE_{forw}	12.29	-0.41	-1.79	-4.54	17.10
	ΔE_{back}	4.12	-0.82	-2.15	-4.80	-1.04
	ΔE_{reac}	8.17	0.41	0.36	0.26	18.13
NH_3	D_{e}	23.17	-2.94	-0.65	3.95	-23.97
(N-H)	ΔE_{forw}	32.04	-0.40	-1.58	-3.95	19.89

	ΔE_{back}	7.85	-4.01	-5.77	-9.29	3.74
	ΔE_{reac}	24.18	3.62	4.20	5.35	16.16
CH_3Cl	D_{e}	13.96	-3.89	-1.31	3.85	-22.83
(OxIn)	ΔE_{forw}	15.77	2.66	2.04	0.80	8.78
(C-Cl)	ΔE_{back}	34.12	0.20	-1.02	-3.47	-2.86
	ΔE_{reac}	-18.35	2.46	3.06	4.27	11.63
CH_3Cl	D_{e}	8.49	-5.20	-2.54	2.76	-24.86
(S_N2)	ΔE_{forw}	47.52	-0.11	2.32	7.19	-1.60
(C-Cl)	ΔE_{back}	71.35	-1.28	0.48	4.00	-11.22
	ΔE_{reac}	-23.83	1.16	1.84	3.20	9.62
		MAD	2.70	2.62	4.85	14.34
		MD	-1.41	-0.96	-0.07	-1.29
		RMS	4.07	4.15	6.33	18.06

Table 9: Deviation from the CCSD(T)/CBS values in kcal/mol for tested density functionals with dispersion correction.

Molecule (bond activation)	Reaction Step	CCSD(T)	BP86-D3	PBE-D3	BLYP-D3	B97-D3
Benzene	D_{e}	28.85	8.90	7.64	1.73	-0.38
(C-H)	ΔE_{forw}	23.67	-3.63	-3.57	-4.20	-3.83
	ΔE_{back}	1.63	2.16	1.97	3.07	2.68
	ΔE_{reac}	22.04	-5.79	-5.17	-7.27	-5.59
BH_3	D_{e}	32.14	10.14	10.74	2.88	1.05
(B-H)	ΔE_{forw}	2.32	-2.11	-1.79	-1.95	-1.60
	ΔE_{back}	-0.76	3.28	2.92	3.98	3.11
	ΔE_{reac}	3.08	-5.40	-4.69	-5.94	-4.56
C_2H_2	D_{e}	36.71	11.38	11.57	3.72	1.92
(C-H)	ΔE_{forw}	25.14	-0.22	-0.05	-0.96	-1.02
	ΔE_{back}	0.53	1.35	0.87	2.11	1.09
	ΔE_{reac}	24.61	-1.57	-0.81	-3.08	-1.75
C_2H_4	D_{e}	38.66	9.36	9.41	2.08	0.26
(C-H)	ΔE_{forw}	33.82	-3.94	-3.37	-4.65	-4.15
	ΔE_{back}	1.41	2.08	1.49	3.13	1.83
	ΔE_{reac}	32.42	-6.03	-4.97	-7.80	-6.01
C_2H_6	D_{e}	10.75	5.22	4.75	0.79	-1.04
(C-C)	ΔE_{forw}	26.06	-5.24	-5.62	-3.24	-2.16
	ΔE_{back}	21.61	4.56	3.20	7.08	5.29
	ΔE_{reac}	4.45	-9.80	-8.81	-10.32	-7.10
Cyclopropane	D_{e}	9.66	5.04	4.47	0.64	-1.29

(C-H)	ΔE_{forw}	10.46	-7.03	-6.73	-5.13	-4.59
	ΔE_{back}	5.14	2.74	1.90	3.93	2.03
	ΔE_{reac}	5.32	-9.77	-8.84	-9.06	-6.98
H_2O	D_{e}	8.95	4.46	4.08	3.33	0.68
(O-H)	ΔE_{forw}	26.57	-9.48	-9.66	-6.76	-5.06
	ΔE_{back}	9.39	1.47	1.39	2.90	1.95
	ΔE_{reac}	17.18	-10.94	-10.77	-9.65	-6.20
C_2H_6	D_{e}	10.75	5.22	4.75	0.79	-1.04
(C-H)	ΔE_{forw}	12.86	-6.14	-5.97	-4.46	-4.15
	ΔE_{back}	4.98	3.05	2.38	4.37	2.63
	ΔE_{reac}	7.88	-9.19	-8.43	-8.82	-6.79
H_2	D_{e}	20.77	7.40	7.85	2.40	0.94
(H-H)	ΔE_{forw}	3.58	-2.38	-2.23	-1.66	-1.30
	ΔE_{back}	-0.74	1.66	1.32	2.40	1.44
	ΔE_{reac}	4.32	-4.04	-3.55	-4.06	-2.75
CH_4	D_{e}	9.92	5.26	5.22	0.79	-0.96
(C-H)	ΔE_{forw}	12.29	-5.66	-5.59	-4.09	-3.82
	ΔE_{back}	4.12	2.63	2.03	4.00	2.47
	ΔE_{reac}	8.17	-8.29	-7.62	-8.09	-6.15
NH_3	D_{e}	23.17	6.35	5.69	3.61	0.88
(N-H)	ΔE_{forw}	32.04	-6.28	-6.64	-4.21	-3.52
	ΔE_{back}	7.85	2.50	2.22	3.77	2.75
	ΔE_{reac}	24.18	-8.77	-8.66	-7.98	-5.63
CH_3Cl	D_{e}	13.96	8.25	7.29	4.60	2.08
(OxIn)	ΔE_{forw}	15.77	-3.27	-3.66	-3.33	-2.25
(C-Cl)	ΔE_{back}	34.12	0.19	-0.33	0.24	-1.28
	ΔE_{reac}	-18.35	-3.46	-3.26	-3.57	-0.69
CH_3Cl	D_{e}	8.49	5.95	5.48	1.43	-0.47
(S_N2)	ΔE_{forw}	47.52	-14.86	-13.98	-19.32	-16.64
(C-Cl)	ΔE_{back}	71.35	-9.11	-8.79	-12.60	-12.62
	ΔE_{reac}	-23.83	-5.76	-5.06	-6.73	-3.24
		MAD	5.55	5.18	4.59	3.30
		MD	-0.91	-0.92	-1.91	-1.95
		RMS	6.41	6.09	5.72	4.46

Table 10: Deviation from the CCSD(T)/CBS values in kcal/mol for tested density functionals with dispersion correction.

Molecule (bond activation)	Reaction Step	CCSD(T)	TPSS-D3	oTPSS-D3	BHLYP-D3	PBE0-D3

Benzene (C-H)	D_e	28.85	7.28	5.25	-12.30	-0.09
	ΔE_{forw}	23.67	-1.87	-2.21	4.37	0.52
	ΔE_{back}	1.63	2.27	2.12	0.27	-0.10
	ΔE_{reac}	22.04	-4.13	-4.32	4.10	0.62
BH_3 (B-H)	D_e	32.14	6.86	6.79	-14.47	0.78
	ΔE_{forw}	2.32	-1.19	-0.46	2.64	0.45
	ΔE_{back}	-0.76	2.43	1.73	-1.50	-0.04
	ΔE_{reac}	3.08	-3.62	-2.19	4.14	0.49
C_2H_2 (C-H)	D_e	36.71	10.87	6.62	-12.36	2.63
	ΔE_{forw}	25.14	1.68	-1.06	0.81	0.87
	ΔE_{back}	0.53	1.95	1.57	-1.78	-1.40
	ΔE_{reac}	24.61	-0.27	-2.63	2.59	2.27
C_2H_4 (C-H)	D_e	38.66	8.55	5.81	-12.49	1.31
	ΔE_{forw}	33.82	-1.69	-2.65	3.26	0.60
	ΔE_{back}	1.41	2.20	2.13	0.46	-0.02
	ΔE_{reac}	32.42	-3.90	-4.79	2.79	0.61
C_2H_6 (C-C)	D_e	10.75	3.06	2.51	-7.85	-0.50
	ΔE_{forw}	26.06	-5.54	-4.25	8.63	0.29
	ΔE_{back}	21.61	5.29	4.45	0.61	0.00
	ΔE_{reac}	4.45	-10.83	-8.70	8.03	0.29
Cyclopropane (C-H)	D_e	9.66	3.01	2.40	-7.96	-0.72
	ΔE_{forw}	10.46	-5.66	-4.43	8.11	-0.34
	ΔE_{back}	5.14	3.24	3.05	0.77	0.30
	ΔE_{reac}	5.32	-8.90	-7.48	7.34	-0.64
H_2O (O-H)	D_e	8.95	3.76	1.56	-3.47	-0.31
	ΔE_{forw}	26.57	-10.57	-8.96	10.83	-0.62
	ΔE_{back}	9.39	3.24	3.62	2.57	0.67
	ΔE_{reac}	17.18	-13.80	-12.59	8.26	-1.29
C_2H_6 (C-H)	D_e	10.75	3.06	2.51	-7.85	-0.50
	ΔE_{forw}	12.86	-4.86	-3.91	6.77	-0.50
	ΔE_{back}	4.98	3.70	3.16	-0.50	-0.09
	ΔE_{reac}	7.88	-8.56	-7.07	7.27	-0.41
H_2 (H-H)	D_e	20.77	3.82	4.98	-8.35	1.61
	ΔE_{forw}	3.58	-1.77	-1.64	3.29	0.20
	ΔE_{back}	-0.74	1.97	1.37	-0.20	-0.11
	ΔE_{reac}	4.32	-3.74	-3.01	3.49	0.30
CH_4 (C-H)	D_e	9.92	3.35	2.71	-7.46	0.07
	ΔE_{forw}	12.29	-4.58	-3.55	6.69	-0.39
	ΔE_{back}	4.12	3.42	2.92	-0.29	-0.21

	ΔE_{reac}	8.17	-7.99	-6.47	6.97	-0.18
NH_3 (N-H)	D_e	23.17	5.01	1.81	-6.91	-0.57
	ΔE_{forw}	32.04	-6.67	-6.74	8.08	-0.55
	ΔE_{back}	7.85	3.65	3.63	1.18	0.45
	ΔE_{reac}	24.18	-10.31	-10.36	6.91	-1.00
CH_3Cl (OxIn) (C-Cl)	D_e	13.96	6.52	3.94	-5.57	1.07
	ΔE_{forw}	15.77	-4.30	-4.93	3.76	0.04
	ΔE_{back}	34.12	1.43	3.01	-1.96	-1.36
	ΔE_{reac}	-18.35	-5.73	-7.95	5.72	1.41
CH_3Cl (S_N2) (C-Cl)	D_e	8.49	3.58	3.24	-7.50	0.00
	ΔE_{forw}	47.52	-15.96	-18.43	-0.84	-3.63
	ΔE_{back}	71.35	-7.30	-9.79	-4.64	-3.98
	ΔE_{reac}	-23.83	-8.66	-8.64	3.80	0.35
		MAD	5.15	4.66	5.21	0.73
		MD	-1.10	-1.47	0.35	-0.03
		RMS	6.13	5.70	6.34	1.10

Table 11: Deviation from the CCSD(T)/CBS values in kcal/mol for tested density functionals with dispersion correction.

Molecule (bond activation)	Reaction Step	CCSD(T)	B3LYP-D3	BMK-D3	PW6B95-D3	TPSSh-D3
Benzene (C-H)	D_e	28.85	-3.77	2.70	-3.37	10.08
	ΔE_{forw}	23.67	-0.62	-0.38	1.88	0.85
	ΔE_{back}	1.63	1.84	6.12	-0.01	1.66
	ΔE_{reac}	22.04	-2.46	-6.50	1.89	-0.82
BH_3 (B-H)	D_e	32.14	-3.78	-0.63	-2.76	7.19
	ΔE_{forw}	2.32	-0.08	-1.38	1.10	-0.44
	ΔE_{back}	-0.76	1.67	1.76	-0.21	1.34
	ΔE_{reac}	3.08	-1.74	-3.14	1.31	-1.78
C_2H_2 (C-H)	D_e	36.71	-2.33	6.72	-1.66	12.08
	ΔE_{forw}	25.14	-0.11	4.05	1.01	2.96
	ΔE_{back}	0.53	0.27	7.18	-1.68	0.98
	ΔE_{reac}	24.61	-0.38	-3.14	2.70	1.98
C_2H_4 (C-H)	D_e	38.66	-3.36	4.60	-2.72	9.92
	ΔE_{forw}	33.82	-1.18	1.35	1.25	0.22
	ΔE_{back}	1.41	1.89	6.19	0.09	1.67
	ΔE_{reac}	32.42	-3.07	-4.84	1.14	-1.46
C_2H_6 (C-C)	D_e	10.75	-2.57	-0.02	-2.72	4.33
	ΔE_{forw}	26.06	1.82	-0.11	0.76	-5.30

	ΔE_{back}	21.61	4.49	12.30	-0.57	3.71
	ΔE_{reac}	4.45	-2.66	-12.41	1.33	-9.00
Cyclopropane	D_{e}	9.66	-2.76	-0.16	-2.76	3.97
(C-H)	ΔE_{forw}	10.46	0.17	-2.00	2.02	-4.16
	ΔE_{back}	5.14	2.52	8.84	0.41	2.82
	ΔE_{reac}	5.32	-2.36	-10.85	1.61	-6.98
H_2O	D_{e}	8.95	0.30	2.56	-1.35	4.28
(O-H)	ΔE_{forw}	26.57	0.24	-1.84	1.07	-6.52
	ΔE_{back}	9.39	2.39	12.43	0.70	3.03
	ΔE_{reac}	17.18	-2.15	-14.27	0.37	-9.56
C_2H_6	D_{e}	10.75	-2.57	-0.02	-2.72	4.33
(C-H)	ΔE_{forw}	12.86	-0.02	-0.26	1.37	-3.21
	ΔE_{back}	4.98	2.24	9.36	-0.16	2.89
	ΔE_{reac}	7.88	-2.26	-9.62	1.53	-6.10
H_2	D_{e}	20.77	-1.44	-1.63	-2.35	2.87
(H-H)	ΔE_{forw}	3.58	0.25	-2.60	0.91	-0.88
	ΔE_{back}	-0.74	1.17	5.52	-0.04	1.38
	ΔE_{reac}	4.32	-0.92	-8.11	0.95	-2.26
CH_4	D_{e}	9.92	-2.35	0.09	-2.13	4.54
(C-H)	ΔE_{forw}	12.29	0.14	-0.39	1.44	-2.79
	ΔE_{back}	4.12	2.08	9.23	-0.25	2.57
	ΔE_{reac}	8.17	-1.94	-9.61	1.69	-5.36
NH_3	D_{e}	23.17	-0.69	2.91	-2.94	5.36
(N-H)	ΔE_{forw}	32.04	0.57	0.00	0.51	-4.05
	ΔE_{back}	7.85	2.39	10.78	0.38	3.09
	ΔE_{reac}	24.18	-1.81	-10.77	0.13	-7.13
CH_3Cl	D_{e}	13.96	0.24	2.29	-1.19	9.29
(OxIn)	ΔE_{forw}	15.77	-0.04	-0.51	-0.89	-3.87
(C-Cl)	ΔE_{back}	34.12	-0.27	14.10	-2.67	1.00
	ΔE_{reac}	-18.35	0.23	-14.62	1.78	-4.87
CH_3Cl	D_{e}	8.49	-2.09	0.01	-2.24	4.97
(S_N2)	ΔE_{forw}	47.52	-10.04	-11.80	-3.74	-10.63
(C-Cl)	ΔE_{back}	71.35	-7.94	5.08	-4.48	-1.45
	ΔE_{reac}	-23.83	-2.10	-16.89	0.74	-9.18
		MAD	1.90	5.47	1.49	4.29
		MD	-0.86	-0.24	-0.26	0.15
		RMS	2.63	7.28	1.81	5.24

Table 12: Deviation from the CCSD(T)/CBS values in kcal/mol for tested density functionals and HF with dispersion correction.

Molecule (bond activation)	Reaction Step	CCSD(T)	DSD-BLYP-D3	B2GPPYLP-D3	B2PLYP-D3	PWPB95-D3	HF-D3
Benzene	D_e	28.85	1.70	0.53	-0.08	-1.17	-22.94
(C-H)	ΔE_{forw}	23.67	1.24	1.56	0.32	1.60	9.89
	ΔE_{back}	1.63	-0.68	-0.53	0.33	0.00	-0.42
	ΔE_{reac}	22.04	1.92	2.09	-0.01	1.60	10.32
BH_3	D_e	32.14	-1.34	-2.27	-1.78	-1.62	-29.01
(B-H)	ΔE_{forw}	2.32	-0.17	0.09	-0.29	0.62	6.42
	ΔE_{back}	-0.76	0.38	0.28	1.08	0.03	-5.75
	ΔE_{reac}	3.08	-0.55	-0.19	-1.38	0.59	12.17
C_2H_2	D_e	36.71	1.78	0.66	0.55	-0.38	-26.54
(C-H)	ΔE_{forw}	25.14	1.49	1.32	0.77	1.27	1.61
	ΔE_{back}	0.53	-1.81	-1.82	-0.90	-1.05	-2.16
	ΔE_{reac}	24.61	3.29	3.13	1.67	2.32	3.76
C_2H_4	D_e	38.66	1.55	0.42	0.11	-1.00	-25.95
(C-H)	ΔE_{forw}	33.82	1.18	1.35	0.18	1.14	6.91
	ΔE_{back}	1.41	-0.75	-0.58	0.27	0.03	0.12
	ΔE_{reac}	32.42	1.92	1.92	-0.10	1.10	6.78
C_2H_6	D_e	10.75	-0.83	-1.30	-1.29	-1.96	-12.49
(C-C)	ΔE_{forw}	26.06	-0.41	0.33	-0.15	0.21	17.00
	ΔE_{back}	21.61	0.95	0.74	2.05	0.20	-4.63
	ΔE_{reac}	4.45	-1.37	-0.41	-2.20	0.01	21.63
Cyclopropane	D_e	9.66	-1.19	-1.62	-1.57	-2.14	-12.85
(C-H)	ΔE_{forw}	10.46	-1.14	-0.21	-0.90	0.57	6.56
	ΔE_{back}	5.14	-0.31	-0.17	0.73	0.37	-13.21
	ΔE_{reac}	5.32	-0.83	-0.04	-1.63	0.20	19.77
H_2O	D_e	8.95	0.44	0.15	0.52	-0.75	-5.65
(O-H)	ΔE_{forw}	26.57	-0.13	0.77	-0.77	-0.21	28.23
	ΔE_{back}	9.39	-1.76	-1.18	-0.07	0.24	7.43
	ΔE_{reac}	17.18	1.63	1.96	-0.70	-0.45	20.79
C_2H_6	D_e	10.75	-0.83	-1.30	-1.29	-1.96	-12.49
(C-H)	ΔE_{forw}	12.86	-0.61	0.10	-0.61	0.46	17.25
	ΔE_{back}	4.98	-0.20	-0.27	0.73	0.19	-2.78
	ΔE_{reac}	7.88	-0.40	0.37	-1.35	0.27	20.03
H_2	D_e	20.77	-0.01	-0.48	-0.08	-1.50	-20.21
(H-H)	ΔE_{forw}	3.58	0.55	0.85	0.41	0.43	7.54
	ΔE_{back}	-0.74	-0.86	-0.78	-0.09	-0.07	-0.67
	ΔE_{reac}	4.32	1.41	1.64	0.50	0.50	8.21
CH_4	D_e	9.92	-0.54	-0.98	-1.00	-1.48	-12.36
(C-H)	ΔE_{forw}	12.29	-0.25	0.41	-0.33	0.64	16.92
	ΔE_{back}	4.12	-0.46	-0.49	0.51	0.07	-1.95
	ΔE_{reac}	8.17	0.21	0.90	-0.84	0.57	18.87
NH_3	D_e	23.17	0.57	-0.03	0.32	-1.73	-13.04
(N-H)	ΔE_{forw}	32.04	0.16	0.85	-0.18	-0.26	20.05
	ΔE_{back}	7.85	-1.33	-0.97	0.22	0.22	3.03
	ΔE_{reac}	24.18	1.50	1.83	-0.39	-0.47	17.03
CH_3Cl	D_e	13.96	1.77	1.27	1.11	-0.47	-11.40
(OxIn)	ΔE_{forw}	15.77	0.44	0.59	-0.24	-0.62	6.05
(C-Cl)	ΔE_{back}	34.12	-1.27	-1.50	-1.07	-1.59	-4.90
	ΔE_{reac}	-18.35	1.71	2.10	0.84	0.97	10.95
CH_3Cl	D_e	8.49	-0.49	-0.92	-0.90	-1.55	-12.31
(S_N2)	ΔE_{forw}	47.52	0.67	0.35	-3.72	-2.63	3.47
(C-Cl)	ΔE_{back}	71.35	1.20	0.43	-2.55	-2.53	-6.57
	ΔE_{reac}	-23.83	-0.54	-0.08	-1.17	-0.10	10.05
		MAD	0.98	0.91	0.82	0.85	11.52
		MD	0.17	0.21	-0.32	-0.22	1.51
		RMS	1.17	1.14	1.10	1.11	13.86

2.2 $PdCl^-$ subset

Table 13: Deviation from the CCSD(T)/CBS values in kcal/mol for tested density functionals without dispersion correction.

Molecule (bond activation)	Reaction Step	CCSD(T)	SVWN	BP86	BLYP	PBE
Benzene	D_e	41.41	14.36	-3.16	-11.28	-0.92
(C-H)	ΔE_{forw}	14.85	-2.38	-2.15	-2.61	-1.53
	ΔE_{back}	0.49	-1.38	0.19	1.13	-0.10
	ΔE_{reac}	14.36	-1.00	-2.35	-3.74	-1.43
C_2H_2	D_e	51.25	18.10	0.97	-7.29	2.98
(C-H)	ΔE_{forw}	11.53	-1.05	-0.90	-1.35	-0.53
	ΔE_{back}	1.39	-3.22	-0.98	0.36	-1.47
	ΔE_{reac}	10.14	2.17	0.08	-1.71	0.94
C_2H_4	D_e	51.99	15.94	-1.38	-9.41	0.69
(C-H)	ΔE_{forw}	27.84	-2.40	-2.77	-3.66	-2.21
	ΔE_{back}	-0.15	-0.77	0.30	1.02	0.05
	ΔE_{reac}	27.99	-1.63	-3.07	-4.68	-2.26
C_2H_6	D_e	20.78	11.36	-3.13	-7.89	-1.64
(C-C)	ΔE_{forw}	28.05	-6.37	-2.99	-0.54	-3.34
	ΔE_{back}	22.71	-4.92	0.15	2.97	-0.52
	ΔE_{reac}	5.34	-1.45	-3.15	-3.51	-2.81
Cyclopropane	D_e	19.72	10.99	-2.76	-7.25	-1.30
(C-H)	ΔE_{forw}	4.03	-5.33	-2.04	0.47	-2.22
	ΔE_{back}	2.72	-1.39	0.49	1.63	0.10
	ΔE_{reac}	1.30	-3.93	-2.52	-1.15	-2.31
H_2O	D_e	17.40	10.93	0.07	-2.25	1.11
(O-H)	ΔE_{forw}	10.87	-7.44	-4.91	-3.48	-4.74
	ΔE_{back}	12.83	-4.14	-2.32	-1.40	-2.59
	ΔE_{reac}	-1.95	-3.31	-2.60	-2.08	-2.15
C_2H_6	D_e	20.78	11.35	-3.14	-7.90	-1.65
(C-H)	ΔE_{forw}	8.12	-5.14	-2.64	-0.71	-2.74
	ΔE_{back}	2.19	-1.35	0.67	1.85	0.27
	ΔE_{reac}	5.93	-3.79	-3.30	-2.56	-3.01
CH_4	D_e	22.05	12.77	-1.52	-6.42	-0.09
(C-H)	ΔE_{forw}	7.24	-4.82	-2.73	-1.05	-2.83
	ΔE_{back}	1.76	-1.41	0.56	1.74	0.17
	ΔE_{reac}	5.47	-3.39	-3.28	-2.78	-2.99
NH_3	D_e	25.67	10.57	-2.45	-5.52	-1.40
(N-H)	ΔE_{forw}	17.48	-7.04	-4.84	-2.73	-4.84

	ΔE_{back}	8.65	-2.43	-0.19	0.96	-0.55
	ΔE_{reac}	8.82	-4.60	-4.63	-3.67	-4.28
CH_3Cl (OxIn) (C-Cl)	D_{e}	21.97	13.92	0.63	-3.58	1.90
	ΔE_{forw}	13.27	-1.88	-2.70	-3.14	-2.56
	ΔE_{back}	56.00	-6.47	-6.77	-7.09	-6.76
	ΔE_{reac}	-42.73	4.60	4.08	3.95	4.20
		MAD	5.79	2.24	3.46	2.00
		MD	1.07	-1.83	-2.66	-1.38
		RMS	7.43	2.72	4.37	2.50

Table 14: Deviation from the CCSD(T)/CBS values in kcal/mol for tested density functionals without dispersion correction.

Molecule (bond activation)	Reaction Step	CCSD(T)	B97-d	TPSS	oTPSS	M06-L
Benzene (C-H)	D_{e}	41.41	-12.08	-2.04	-3.78	-3.66
	ΔE_{forw}	14.85	-2.13	-0.45	-1.27	3.33
	ΔE_{back}	0.49	0.63	0.83	1.05	-1.26
	ΔE_{reac}	14.36	-2.77	-1.27	-2.32	4.59
C_2H_2 (C-H)	D_{e}	51.25	-8.24	2.39	-0.82	-1.08
	ΔE_{forw}	11.53	-1.24	0.88	-1.68	2.98
	ΔE_{back}	1.39	-0.41	0.43	0.65	-2.46
	ΔE_{reac}	10.14	-0.83	0.45	-2.33	5.44
C_2H_4 (C-H)	D_{e}	51.99	-10.41	-0.23	-1.86	-2.20
	ΔE_{forw}	27.84	-3.21	-0.94	-2.08	2.72
	ΔE_{back}	-0.15	0.63	0.73	0.92	-0.69
	ΔE_{reac}	27.99	-3.84	-1.68	-3.00	3.41
C_2H_6 (C-C)	D_{e}	20.78	-9.37	-3.41	-4.06	-4.04
	ΔE_{forw}	28.05	0.13	-3.36	-2.54	0.37
	ΔE_{back}	22.71	1.87	2.56	3.05	-0.93
	ΔE_{reac}	5.34	-1.74	-5.92	-5.59	1.31
Cyclopropane (C-H)	D_{e}	19.72	-8.71	-3.19	-3.85	-4.15
	ΔE_{forw}	4.03	0.46	-1.43	-1.23	2.83
	ΔE_{back}	2.72	0.86	1.87	2.14	-0.59
	ΔE_{reac}	1.30	-0.39	-3.30	-3.36	3.43
H_2O (O-H)	D_{e}	17.40	-4.57	0.49	-1.26	-0.34
	ΔE_{forw}	10.87	-2.03	-6.11	-5.04	1.29
	ΔE_{back}	12.83	-2.24	0.26	1.06	-1.35
	ΔE_{reac}	-1.95	0.21	-6.39	-6.11	2.63
C_2H_6	D_{e}	20.78	-9.38	-3.42	-4.07	-4.05

(C-H)	ΔE_{forw}	8.12	-0.71	-1.91	-1.76	2.51
	ΔE_{back}	2.19	1.03	1.85	1.91	-0.44
	ΔE_{reac}	5.93	-1.75	-3.76	-3.68	2.94
CH_4	D_{e}	22.05	-8.10	-2.25	-2.78	-3.96
(C-H)	ΔE_{forw}	7.24	-1.06	-2.01	-1.88	2.55
	ΔE_{back}	1.76	0.87	1.86	2.03	-0.65
	ΔE_{reac}	5.47	-1.92	-3.85	-3.90	3.20
NH_3	D_{e}	25.67	-8.00	-1.88	-4.50	-5.12
(N-H)	ΔE_{forw}	17.48	-2.60	-4.92	-5.34	-0.71
	ΔE_{back}	8.65	0.04	1.96	2.60	-0.12
	ΔE_{reac}	8.82	-2.63	-6.87	-7.93	-0.57
CH_3Cl	D_{e}	21.97	-5.34	0.75	-1.76	-1.12
(OxIn)	ΔE_{forw}	13.27	-2.08	-3.79	-4.61	-1.71
(C-Cl)	ΔE_{back}	56.00	-7.61	-3.98	-1.39	-4.45
	ΔE_{reac}	-42.73	5.53	0.20	-3.21	2.74
		MAD	3.44	2.40	2.86	2.35
		MD	-2.83	-1.52	-2.09	0.07
		RMS	4.81	2.99	3.29	2.77

Table 15: Deviation from the CCSD(T)/CBS values in kcal/mol for tested density functionals without dispersion correction.

Molecule (bond activation)	Reaction Step	CCSD(T)	B3LYP	BHLYP	PBE0	PW6B95
Benzene	D_{e}	41.41	-12.49	-17.21	-4.87	-7.47
(C-H)	ΔE_{forw}	14.85	-0.82	1.69	0.49	1.68
	ΔE_{back}	0.49	1.12	1.95	0.10	0.30
	ΔE_{reac}	14.36	-1.94	-0.26	0.39	1.38
C_2H_2	D_{e}	51.25	-8.34	-13.16	-0.62	-4.36
(C-H)	ΔE_{forw}	11.53	0.41	2.53	1.53	2.27
	ΔE_{back}	1.39	1.12	3.39	-0.24	0.36
	ΔE_{reac}	10.14	-0.71	-0.85	1.77	1.92
C_2H_4	D_{e}	51.99	-9.86	-13.97	-2.20	-5.56
(C-H)	ΔE_{forw}	27.84	-0.99	2.15	0.87	1.46
	ΔE_{back}	-0.15	0.88	1.34	0.02	0.17
	ΔE_{reac}	27.99	-1.87	0.81	0.85	1.29
C_2H_6	D_{e}	20.78	-7.87	-9.76	-3.68	-4.89
(C-C)	ΔE_{forw}	28.05	2.84	8.38	1.02	1.45
	ΔE_{back}	22.71	2.76	3.56	-0.16	-0.36
	ΔE_{reac}	5.34	0.08	4.82	1.18	1.81

Cyclopropane (C-H)	D_e	19.72	-7.74	-9.89	-3.85	-5.16
	ΔE_{forw}	4.03	2.90	7.66	1.00	3.14
	ΔE_{back}	2.72	1.64	2.60	0.36	0.65
	ΔE_{reac}	1.30	1.27	5.06	0.66	2.50
H_2O (O-H)	D_e	17.40	-3.27	-5.50	-1.68	-1.87
	ΔE_{forw}	10.87	0.58	7.14	0.37	1.97
	ΔE_{back}	12.83	0.52	4.50	-0.01	0.44
	ΔE_{reac}	-1.95	0.04	2.63	0.37	1.52
C_2H_6 (C-H)	D_e	20.78	-7.87	-9.77	-3.68	-4.89
	ΔE_{forw}	8.12	1.92	6.72	0.60	2.59
	ΔE_{back}	2.19	1.52	1.82	0.13	0.28
	ΔE_{reac}	5.93	0.40	4.90	0.47	2.32
CH_4 (C-H)	D_e	22.05	-6.94	-9.66	-2.69	-4.70
	ΔE_{forw}	7.24	1.45	5.96	0.30	2.25
	ΔE_{back}	1.76	1.52	2.03	0.17	0.36
	ΔE_{reac}	5.47	-0.07	3.94	0.14	1.90
NH_3 (N-H)	D_e	25.67	-5.65	-7.33	-3.33	-4.58
	ΔE_{forw}	17.48	0.78	6.64	-0.44	0.80
	ΔE_{back}	8.65	1.57	3.76	0.48	0.82
	ΔE_{reac}	8.82	-0.78	2.89	-0.91	-0.02
CH_3Cl (OxIn) (C-Cl)	D_e	21.97	-5.24	-8.70	-2.00	-3.76
	ΔE_{forw}	13.27	-0.94	2.26	0.08	-1.23
	ΔE_{back}	56.00	-3.98	0.40	-2.85	-3.80
	ΔE_{reac}	-42.73	3.03	1.85	2.93	2.57
		MAD	2.89	5.24	1.24	2.27
		MD	-1.48	-0.07	-0.42	-0.36
		RMS	4.20	6.55	1.78	2.88

Table 16: Deviation from the CCSD(T)/CBS values in kcal/mol for tested density functionals without dispersion correction.

Molecule (bond activation)	Reaction Step	CCSD(T)	BMK	TPSSh	M05	M05-2X
Benzene (C-H)	D_e	41.41	-6.06	-3.55	-12.21	-9.31
	ΔE_{forw}	14.85	-2.11	0.25	6.57	1.62
	ΔE_{back}	0.49	5.08	0.87	-2.57	1.07
	ΔE_{reac}	14.36	-7.18	-0.62	9.14	0.55
C_2H_2 (C-H)	D_e	51.25	2.27	0.94	-10.06	-8.10
	ΔE_{forw}	11.53	3.50	1.58	5.58	1.36

	ΔE_{back}	1.39	7.99	0.83	-3.67	1.99
	ΔE_{reac}	10.14	-4.49	0.75	9.25	-0.63
C_2H_4	D_{e}	51.99	-0.69	-1.34	-10.40	-8.30
(C-H)	ΔE_{forw}	27.84	0.87	0.18	5.99	1.09
	ΔE_{back}	-0.15	3.70	0.69	-2.38	0.78
	ΔE_{reac}	27.99	-2.83	-0.51	8.37	0.31
C_2H_6	D_{e}	20.78	-4.56	-4.06	-9.20	-5.47
(C-C)	ΔE_{forw}	28.05	3.50	-1.57	2.39	4.48
	ΔE_{back}	22.71	14.64	2.46	-6.68	1.33
	ΔE_{reac}	5.34	-11.14	-4.03	9.07	3.15
Cyclopropane	D_{e}	19.72	-5.15	-4.07	-9.21	-5.90
(C-H)	ΔE_{forw}	4.03	-0.06	-0.17	6.86	4.29
	ΔE_{back}	2.72	8.93	1.89	-3.78	1.57
	ΔE_{reac}	1.30	-8.98	-2.06	10.65	2.72
H_2O	D_{e}	17.40	0.91	-0.65	-5.54	-1.87
(O-H)	ΔE_{forw}	10.87	-1.16	-3.93	7.83	2.77
	ΔE_{back}	12.83	11.51	1.15	-3.07	1.61
	ΔE_{reac}	-1.95	-12.68	-5.09	10.89	1.15
C_2H_6	D_{e}	20.78	-4.56	-4.06	-9.21	-5.47
(C-H)	ΔE_{forw}	8.12	1.21	-0.59	6.44	3.77
	ΔE_{back}	2.19	8.24	1.72	-4.10	1.03
	ΔE_{reac}	5.93	-7.03	-2.32	10.54	2.74
CH_4	D_{e}	22.05	-3.65	-3.07	-8.61	-5.40
(C-H)	ΔE_{forw}	7.24	0.62	-0.77	5.79	3.32
	ΔE_{back}	1.76	8.64	1.78	-4.07	1.06
	ΔE_{reac}	5.47	-8.00	-2.53	9.87	2.26
NH_3	D_{e}	25.67	1.48	-2.65	-9.45	-4.83
(N-H)	ΔE_{forw}	17.48	2.24	-3.09	4.60	1.91
	ΔE_{back}	8.65	11.28	2.25	-3.39	1.51
	ΔE_{reac}	8.82	-9.03	-5.34	8.00	0.41
CH_3Cl	D_{e}	21.97	-3.20	-0.81	-7.97	-4.89
(OxIn)	ΔE_{forw}	13.27	-2.72	-2.63	1.09	-0.56
(C-Cl)	ΔE_{back}	56.00	11.24	-2.58	-8.54	-1.27
	ΔE_{reac}	-42.73	-13.96	-0.06	9.62	0.71
		MAD	5.68	1.99	7.07	2.81
		MD	-0.28	-1.12	0.36	-0.29
		RMS	6.97	2.44	7.62	3.63

Table 17: Deviation from the CCSD(T)/CBS values in kcal/mol for tested density functionals without dispersion correction.

Molecule (bond activation)	Reaction Step	CCSD(T)	M06	M06-2X	M06-HF	1DH-BLYP
Benzene	D_e	41.41	-8.28	-12.85	-13.23	0.71
(C-H)	ΔE_forw	14.85	5.75	2.95	-2.43	1.75
	ΔE_back	0.49	-2.68	-0.73	3.56	-2.14
	ΔE_reac	14.36	8.43	3.68	-5.99	3.89
C_2H_2	D_e	51.25	-9.01	-13.33	-12.60	1.28
(C-H)	ΔE_forw	11.53	4.48	1.15	-3.12	1.42
	ΔE_back	1.39	-3.61	-1.29	5.01	-3.68
	ΔE_reac	10.14	8.09	2.44	-8.12	5.10
C_2H_4	D_e	51.99	-8.03	-12.46	-12.28	1.34
(C-H)	ΔE_forw	27.84	4.49	1.78	-2.72	1.81
	ΔE_back	-0.15	-2.04	-0.52	2.30	-1.59
	ΔE_reac	27.99	6.53	2.30	-5.02	3.40
C_2H_6	D_e	20.78	-4.71	-8.04	-9.29	-1.49
(C-C)	ΔE_forw	28.05	1.60	5.35	4.33	-1.04
	ΔE_back	22.71	-7.40	-2.29	5.73	-2.40
	ΔE_reac	5.34	9.00	7.64	-1.40	1.36
Cyclopropane	D_e	19.72	-5.23	-8.28	-9.78	-1.62
(C-H)	ΔE_forw	4.03	6.43	6.19	1.33	-0.75
	ΔE_back	2.72	-3.83	-1.04	4.53	-2.57
	ΔE_reac	1.30	10.27	7.25	-3.19	1.83
H_2O	D_e	17.40	-2.29	-3.84	-5.23	-0.01
(O-H)	ΔE_forw	10.87	8.57	6.78	-0.27	-0.73
	ΔE_back	12.83	-4.03	-2.29	3.46	-4.35
	ΔE_reac	-1.95	12.59	9.06	-3.74	3.60
C_2H_6	D_e	20.78	-4.71	-8.04	-9.28	-1.50
(C-H)	ΔE_forw	8.12	5.78	5.47	1.37	-0.95
	ΔE_back	2.19	-3.77	-1.18	3.68	-1.92
	ΔE_reac	5.93	9.55	6.65	-2.31	0.98
CH_4	D_e	22.05	-4.50	-7.79	-9.15	-1.45
(C-H)	ΔE_forw	7.24	5.78	4.92	0.62	-0.85
	ΔE_back	1.76	-3.96	-1.38	3.66	-2.14
	ΔE_reac	5.47	9.75	6.31	-3.03	1.30
NH_3	D_e	25.67	-5.22	-8.55	-8.77	-0.13
(N-H)	ΔE_forw	17.48	5.21	3.68	-1.11	-0.53
	ΔE_back	8.65	-3.98	-2.07	3.60	-3.81

	ΔE_{reac}	8.82	9.19	5.76	-4.70	3.29
CH_3Cl (OxIn) (C-Cl)	D_{e}	21.97	-3.60	-8.41	-9.41	0.73
	ΔE_{forw}	13.27	-0.46	0.29	0.97	-0.06
	ΔE_{back}	56.00	-10.95	-5.39	2.86	-5.09
	ΔE_{reac}	-42.73	10.49	5.67	-1.89	5.03
		MAD	6.11	5.13	4.88	1.99
		MD	0.99	-0.36	-2.53	-0.05
		RMS	6.74	6.18	5.97	2.43

Table 18: Deviation from the CCSD(T)/CBS values in kcal/mol for tested density functionals without dispersion correction.

Molecule (bond activation)	Reaction Step	CCSD(T)	1DH-PBE	PBE0-2	PBE0-DH	PTPSS	mPW2PLYP
Benzene (C-H)	D_{e}	41.41	6.54	4.56	-2.99	-1.83	-4.19
	ΔE_{forw}	14.85	2.35	3.22	2.07	1.31	1.77
	ΔE_{back}	0.49	-2.99	-3.04	-0.37	-0.35	-0.69
	ΔE_{reac}	14.36	5.34	6.27	2.44	1.66	2.46
C_2H_2 (C-H)	D_{e}	51.25	6.90	5.02	-0.05	-0.24	-2.90
	ΔE_{forw}	11.53	1.81	2.71	2.33	1.62	1.48
	ΔE_{back}	1.39	-4.92	-4.41	-0.68	-0.89	-1.41
	ΔE_{reac}	10.14	6.73	7.12	3.01	2.51	2.89
C_2H_4 (C-H)	D_{e}	51.99	6.99	5.41	-0.87	-0.73	-3.11
	ΔE_{forw}	27.84	2.68	4.01	2.58	1.26	1.55
	ΔE_{back}	-0.15	-2.25	-2.36	-0.37	-0.18	-0.50
	ΔE_{reac}	27.99	4.93	6.36	2.95	1.44	2.06
C_2H_6 (C-C)	D_{e}	20.78	1.52	0.51	-2.92	-2.36	-3.34
	ΔE_{forw}	28.05	-2.88	-0.67	1.73	0.16	1.39
	ΔE_{back}	22.71	-4.51	-4.47	-1.10	0.82	-0.59
	ΔE_{reac}	5.34	1.63	3.80	2.83	-0.65	1.98
Cyclopropane (C-H)	D_{e}	19.72	1.23	0.16	-3.22	-2.70	-3.44
	ΔE_{forw}	4.03	-2.69	-1.13	1.58	0.45	1.97
	ΔE_{back}	2.72	-3.63	-3.75	-0.29	0.13	-0.74
	ΔE_{reac}	1.30	0.95	2.63	1.88	0.32	2.73
H_2O (O-H)	D_{e}	17.40	1.25	0.13	-1.83	0.27	-0.93
	ΔE_{forw}	10.87	-1.66	0.91	2.07	-1.19	1.30
	ΔE_{back}	12.83	-5.30	-4.33	0.19	-0.90	-1.69
	ΔE_{reac}	-1.95	3.63	5.23	1.87	-0.30	2.98
C_2H_6 (C-H)	D_{e}	20.78	1.51	0.50	-2.93	-2.37	-3.35
	ΔE_{forw}	8.12	-2.48	-0.91	1.42	0.17	1.47

	ΔE_{back}	2.19	-2.94	-3.24	-0.54	0.38	-0.57
	ΔE_{reac}	5.93	0.47	2.33	1.96	-0.21	2.05
CH_4 (C-H)	D_{e}	22.05	1.60	0.28	-2.53	-1.99	-3.27
	ΔE_{forw}	7.24	-2.21	-0.75	1.20	0.19	1.32
	ΔE_{back}	1.76	-3.16	-3.38	-0.48	0.34	-0.65
	ΔE_{reac}	5.47	0.96	2.65	1.68	-0.15	1.98
NH_3 (N-H)	D_{e}	25.67	1.74	0.88	-2.70	-1.10	-2.03
	ΔE_{forw}	17.48	-1.94	0.23	1.08	-0.68	1.20
	ΔE_{back}	8.65	-4.96	-4.76	-0.04	-0.15	-1.24
	ΔE_{reac}	8.82	3.04	4.99	1.13	-0.52	2.46
CH_3Cl (OxIn) (C-Cl)	D_{e}	21.97	3.29	1.75	-1.73	-0.69	-1.36
	ΔE_{forw}	13.27	0.32	1.90	1.22	-1.39	-0.09
	ΔE_{back}	56.00	-4.88	-3.31	-1.69	-2.43	-4.22
	ΔE_{reac}	-42.73	5.20	5.21	2.90	1.04	4.13
		MAD	3.15	2.98	1.69	0.95	1.99
		MD	0.48	0.96	0.32	-0.25	-0.03
		RMS	3.65	3.56	1.94	1.22	2.26

Table 19: Deviation from the CCSD(T)/CBS values in kcal/mol for tested density functionals without dispersion correction.

Molecule (bond activation)	Reaction Step	CCSD(T)	B2GPPLYP	B2PLYP	PWPB95	DSD-BLYP
Benzene (C-H)	D_{e}	41.41	-2.18	-3.76	-3.98	-0.95
	ΔE_{forw}	14.85	1.92	1.15	1.75	1.88
	ΔE_{back}	0.49	-1.44	-0.78	-0.25	-1.92
	ΔE_{reac}	14.36	3.35	1.93	2.00	3.80
C_2H_2 (C-H)	D_{e}	51.25	-1.12	-2.28	-2.20	-0.22
	ΔE_{forw}	11.53	1.71	1.07	1.98	1.70
	ΔE_{back}	1.39	-2.41	-1.77	-0.58	-3.11
	ΔE_{reac}	10.14	4.13	2.84	2.56	4.81
C_2H_4 (C-H)	D_{e}	51.99	-1.12	-2.69	-2.76	-0.07
	ΔE_{forw}	27.84	2.09	0.93	1.62	2.14
	ΔE_{back}	-0.15	-1.08	-0.56	-0.21	-1.43
	ΔE_{reac}	27.99	3.16	1.49	1.83	3.57
C_2H_6 (C-C)	D_{e}	20.78	-2.79	-3.68	-3.50	-2.40
	ΔE_{forw}	28.05	0.80	0.37	0.73	0.25
	ΔE_{back}	22.71	-1.35	-0.48	-0.39	-1.68
	ΔE_{reac}	5.34	2.15	0.84	1.12	1.94
Cyclopropane	D_{e}	19.72	-2.95	-3.72	-3.90	-2.55

(C-H)	ΔE_{forw}	4.03	0.81	0.83	1.45	-0.01
	ΔE_{back}	2.72	-1.67	-0.83	-0.03	-2.26
	ΔE_{reac}	1.30	2.49	1.67	1.49	2.26
H_2O	D_{e}	17.40	-0.93	-1.13	-1.07	-0.70
(O-H)	ΔE_{forw}	10.87	0.88	-0.07	0.81	0.27
	ΔE_{back}	12.83	-2.65	-2.19	-0.69	-3.63
	ΔE_{reac}	-1.95	3.52	2.11	1.48	3.88
C_2H_6	D_{e}	20.78	-2.80	-3.68	-3.50	-2.41
(C-H)	ΔE_{forw}	8.12	0.53	0.38	1.19	-0.22
	ΔE_{back}	2.19	-1.30	-0.51	-0.04	-1.69
	ΔE_{reac}	5.93	1.83	0.89	1.23	1.47
CH_4	D_{e}	22.05	-2.86	-3.43	-3.24	-2.43
(C-H)	ΔE_{forw}	7.24	0.45	0.29	1.03	-0.23
	ΔE_{back}	1.76	-1.43	-0.64	-0.02	-1.87
	ΔE_{reac}	5.47	1.89	0.94	1.06	1.65
NH_3	D_{e}	25.67	-1.30	-2.04	-2.85	-0.86
(N-H)	ΔE_{forw}	17.48	0.90	0.20	0.16	0.37
	ΔE_{back}	8.65	-2.43	-1.51	-0.23	-3.32
	ΔE_{reac}	8.82	3.35	1.72	0.41	3.70
CH_3Cl	D_{e}	21.97	-0.82	-1.30	-2.37	-0.42
(OxIn)	ΔE_{forw}	13.27	0.50	-0.50	-0.97	0.52
(C-Cl)	ΔE_{back}	56.00	-3.93	-4.57	-3.33	-4.17
	ΔE_{reac}	-42.73	4.43	4.07	2.35	4.69
		MAD	1.99	1.65	1.56	1.94
		MD	0.06	-0.46	-0.25	0.01
		RMS	2.25	2.05	1.94	2.37

Table 20: Deviation from the CCSD(T)/CBS values in kcal/mol for tested wave-function methods.

Molecule (bond activation)	Reaction Step	CCSD(T)	SOS-MP2	SCS-MP2	MP2	HF
Benzene	D_{e}	41.41	-0.33	4.03	12.75	-35.23
(C-H)	ΔE_{forw}	14.85	1.66	2.23	3.36	3.43
	ΔE_{back}	0.49	-3.56	-5.02	-7.95	6.55
	ΔE_{reac}	14.36	5.22	7.25	11.31	-3.11
C_2H_2	D_{e}	51.25	-0.26	3.60	11.31	-31.80
(C-H)	ΔE_{forw}	11.53	2.39	2.59	3.01	3.03
	ΔE_{back}	1.39	-4.69	-6.85	-11.17	10.73
	ΔE_{reac}	10.14	7.08	9.45	14.18	-7.70

C_2H_4 (C-H)	D_e	51.99	0.65	4.71	12.83	-31.94
	ΔE_{forw}	27.84	3.15	3.79	5.07	3.40
	ΔE_{back}	-0.15	-2.65	-3.76	-5.98	4.69
	ΔE_{reac}	27.99	5.80	7.55	11.05	-1.29
C_2H_6 (C-C)	D_e	20.78	-3.21	-0.84	3.92	-20.88
	ΔE_{forw}	28.05	1.54	-0.62	-4.92	18.39
	ΔE_{back}	22.71	-1.58	-4.47	-10.26	10.16
	ΔE_{reac}	5.34	3.12	3.86	5.33	8.22
Cyclopropane (C-H)	D_e	19.72	-3.30	-0.98	3.66	-20.89
	ΔE_{forw}	4.03	-2.42	-3.88	-6.79	15.86
	ΔE_{back}	2.72	-5.21	-7.19	-11.14	7.49
	ΔE_{reac}	1.30	2.80	3.32	4.35	8.39
H_2O (O-H)	D_e	17.40	-1.25	0	2.49	-14.36
	ΔE_{forw}	10.87	-0.07	-0.71	-2.00	18.39
	ΔE_{back}	12.83	-6.63	-9.00	-13.75	15.30
	ΔE_{reac}	-1.95	6.55	8.28	11.74	3.08
C_2H_6 (C-H)	D_e	20.78	-3.22	-0.84	3.91	-20.86
	ΔE_{forw}	8.12	-1.79	-3.13	-5.80	15.24
	ΔE_{back}	2.19	-3.21	-5.01	-8.61	5.78
	ΔE_{reac}	5.93	1.42	1.88	2.81	9.45
CH_4 (C-H)	D_e	22.05	-2.90	-0.69	3.74	-20.45
	ΔE_{forw}	7.24	-1.63	-2.81	-5.15	13.87
	ΔE_{back}	1.76	-3.51	-5.39	-9.15	6.44
	ΔE_{reac}	5.47	1.88	2.59	4.01	7.44
NH_3 (N-H)	D_e	25.67	-0.52	1.24	4.75	-17.27
	ΔE_{forw}	17.48	0.37	-0.67	-2.75	16.49
	ΔE_{back}	8.65	-6.63	-9.11	-14.07	12.39
	ΔE_{reac}	8.82	7.01	8.45	11.33	4.11
CH_3Cl (OxIn) (C-Cl)	D_e	21.97	-2.17	0.34	5.37	-20.82
	ΔE_{forw}	13.27	2.67	2.48	2.10	5.68
	ΔE_{back}	56.00	-2.47	-4.49	-8.55	7.74
	ΔE_{reac}	-42.73	5.14	6.98	10.65	-2.06
		MAD	3.04	4.00	7.33	12.26
		MD	-0.12	0.23	0.92	-0.17
		RMS	3.63	4.85	8.27	14.89

Table 21: Deviation from the CCSD(T)/CBS values in kcal/mol for tested density functionals with dispersion correction.

Molecule (bond activation)	Reaction Step	CCSD(T)	BP86-D3	PBE-D3	BLYP-D3	B97-D3
Benzene	D_e	41.41	2.71	1.98	-4.63	-5.82
(C-H)	ΔE_forw	14.85	-1.37	-1.32	-1.97	-1.76
	ΔE_back	0.49	0.19	0.11	0.87	1.00
	ΔE_reac	14.36	-1.56	-1.10	-2.83	-2.01
C_2H_2	D_e	51.25	3.80	4.46	-3.88	-4.78
(C-H)	ΔE_forw	11.53	-0.78	-0.46	-1.16	-1.05
	ΔE_back	1.39	-0.98	-1.40	0.34	-0.22
	ΔE_reac	10.14	0.20	1.02	-1.50	-0.55
C_2H_4	D_e	51.99	1.73	2.34	-5.64	-6.54
(C-H)	ΔE_forw	27.84	-3.04	-2.36	-3.94	-3.51
	ΔE_back	-0.15	0.30	-0.10	0.98	0.33
	ΔE_reac	27.99	-3.34	-2.39	-4.92	-4.01
C_2H_6	D_e	20.78	0.24	0.18	-3.87	-5.08
(C-C)	ΔE_forw	28.05	-3.85	-3.78	-1.64	-0.98
	ΔE_back	22.71	0.15	-0.97	2.84	0.76
	ΔE_reac	5.34	-4.00	-3.19	-4.49	-2.35
Cyclopropane	D_e	19.72	0.65	0.50	-3.32	-4.64
(C-H)	ΔE_forw	4.03	-2.85	-2.62	-0.51	-0.46
	ΔE_back	2.72	0.49	-0.29	1.62	-0.06
	ΔE_reac	1.30	-3.33	-2.69	-2.11	-1.11
H_2O	D_e	17.40	1.52	1.95	-0.37	-2.38
(O-H)	ΔE_forw	10.87	-5.10	-4.80	-3.60	-2.16
	ΔE_back	12.83	-2.32	-2.66	-1.29	-2.38
	ΔE_reac	-1.95	-2.80	-2.26	-2.32	0.00
C_2H_6	D_e	20.78	0.23	0.17	-3.87	-5.08
(C-H)	ΔE_forw	8.12	-3.06	-2.93	-1.18	-1.16
	ΔE_back	2.19	0.67	0.07	1.87	0.59
	ΔE_reac	5.93	-3.72	-3.20	-3.05	-2.08
CH_4	D_e	22.05	0.91	1.27	-3.39	-4.73
(C-H)	ΔE_forw	7.24	-2.90	-2.91	-1.23	-1.24
	ΔE_back	1.76	0.56	0.09	1.75	0.69
	ΔE_reac	5.47	-3.45	-3.07	-2.97	-2.02
NH_3	D_e	25.67	-0.43	-0.26	-2.96	-5.08
(N-H)	ΔE_forw	17.48	-5.05	-4.91	-2.87	-2.76
	ΔE_back	8.65	-0.19	-0.62	1.08	-0.11
	ΔE_reac	8.82	-4.85	-4.40	-3.94	-2.81
CH_3Cl	D_e	21.97	3.72	3.43	-0.08	-2.04
(OxIn)	ΔE_forw	13.27	-3.75	-2.92	-4.00	-3.03

(C-Cl)	ΔE_{back}	56.00	-6.77	-7.13	-6.66	-8.56
	ΔE_{reac}	-42.73	3.02	3.64	2.66	4.30
		MAD	2.26	2.15	2.60	2.51
		MD	-1.21	-1.09	-1.90	-2.12
		RMS	2.83	2.70	3.02	3.23

Table 22: Deviation from the CCSD(T)/CBS values in kcal/mol for tested density functionals with dispersion correction.

Molecule (bond activation)	Reaction Step	CCSD(T)	TPSS-D3	oTPSS-D3	BHLYP-D3	PBE0-D3
Benzene	D_{e}	41.41	1.95	1.55	-12.42	-2.00
(C-H)	ΔE_{forw}	14.85	-0.13	-0.82	2.41	0.71
	ΔE_{back}	0.49	0.67	0.84	1.95	-0.01
	ΔE_{reac}	14.36	-0.80	-1.66	0.46	0.72
C_2H_2	D_{e}	51.25	4.36	1.84	-10.67	0.78
(C-H)	ΔE_{forw}	11.53	0.96	-1.56	2.72	1.58
	ΔE_{back}	1.39	0.43	0.64	3.39	-0.24
	ΔE_{reac}	10.14	0.53	-2.21	-0.66	1.83
C_2H_4	D_{e}	51.99	1.96	1.09	-11.27	-0.66
(C-H)	ΔE_{forw}	27.84	-1.15	-2.35	2.01	0.71
	ΔE_{back}	-0.15	0.72	0.89	1.34	0.02
	ΔE_{reac}	27.99	-1.87	-3.24	0.68	0.70
C_2H_6	D_{e}	20.78	-0.99	-0.82	-7.01	-1.95
(C-C)	ΔE_{forw}	28.05	-3.94	-3.34	7.57	0.62
	ΔE_{back}	22.71	2.52	2.98	3.56	-0.18
	ΔE_{reac}	5.34	-6.46	-6.32	4.00	0.80
Cyclopropane	D_{e}	19.72	-0.76	-0.63	-7.20	-2.11
(C-H)	ΔE_{forw}	4.03	-1.96	-1.95	6.89	0.64
	ΔE_{back}	2.72	1.87	2.13	2.60	0.36
	ΔE_{reac}	1.30	-3.82	-4.07	4.29	0.29
H_2O	D_{e}	17.40	1.57	0.20	-4.24	-0.93
(O-H)	ΔE_{forw}	10.87	-6.20	-5.15	6.98	0.31
	ΔE_{back}	12.83	0.33	1.15	4.50	0.03
	ΔE_{reac}	-1.95	-6.53	-6.31	2.48	0.27
C_2H_6	D_{e}	20.78	-0.99	-0.82	-7.01	-1.96
(C-H)	ΔE_{forw}	8.12	-2.17	-2.12	6.34	0.42
	ΔE_{back}	2.19	1.86	1.93	1.82	0.14
	ΔE_{reac}	5.93	-4.03	-4.05	4.52	0.28
CH_4	D_{e}	22.05	-0.47	-0.39	-7.58	-1.44

(C-H)	ΔE_{forw}	7.24	-2.10	-2.01	5.82	0.23
	ΔE_{back}	1.76	1.87	2.05	2.03	0.19
	ΔE_{reac}	5.47	-3.97	-4.05	3.80	0.06
NH_3 (N-H)	D_{e}	25.67	-0.39	-2.49	-5.60	-2.30
	ΔE_{forw}	17.48	-5.01	-5.46	6.45	-0.50
	ΔE_{back}	8.65	2.03	2.70	3.76	0.53
	ΔE_{reac}	8.82	-7.03	-8.15	2.71	-1.02
CH_3Cl (OxIn) (C-Cl)	D_{e}	21.97	2.84	1.03	-6.10	-0.49
	ΔE_{forw}	13.27	-4.25	-5.25	1.31	-0.24
	ΔE_{back}	56.00	-3.71	-1.03	0.40	-2.65
	ΔE_{reac}	-42.73	-0.55	-4.22	0.91	2.41
		MAD	2.39	2.54	4.44	0.83
		MD	-1.07	-1.49	0.45	-0.10
		RMS	3.06	3.15	5.34	1.12

Table 23: Deviation from the CCSD(T)/CBS values in kcal/mol for tested density functionals with dispersion correction.

Molecule (bond activation)	Reaction Step	CCSD(T)	B3LYP-D3	BMK-D3	PW6B95-D3	TPSSh-D3
Benzene (C-H)	D_{e}	41.41	-6.91	-0.45	-5.23	5.16
	ΔE_{forw}	14.85	-0.05	-1.31	1.91	1.54
	ΔE_{back}	0.49	1.12	4.94	0.23	0.57
	ΔE_{reac}	14.36	-1.17	-6.24	1.68	0.97
C_2H_2 (C-H)	D_{e}	51.25	-5.49	4.93	-3.33	6.85
	ΔE_{forw}	11.53	0.59	3.64	2.31	2.73
	ΔE_{back}	1.39	1.12	7.99	0.35	0.75
	ΔE_{reac}	10.14	-0.53	-4.34	1.95	1.99
C_2H_4 (C-H)	D_{e}	51.99	-6.71	2.16	-4.45	4.58
	ΔE_{forw}	27.84	-1.19	0.67	1.35	0.58
	ΔE_{back}	-0.15	0.88	3.71	0.17	0.62
	ΔE_{reac}	27.99	-2.06	-3.03	1.20	-0.03
C_2H_6 (C-C)	D_{e}	20.78	-4.53	-1.65	-3.67	0.68
	ΔE_{forw}	28.05	2.00	2.69	1.17	-4.16
	ΔE_{back}	22.71	2.76	14.73	-0.33	2.27
	ΔE_{reac}	5.34	-0.76	-12.04	1.50	-6.43
Cyclopropane (C-H)	D_{e}	19.72	-4.47	-2.18	-3.89	0.34
	ΔE_{forw}	4.03	2.09	-0.83	2.89	-1.61
	ΔE_{back}	2.72	1.64	9.02	0.68	2.05
	ΔE_{reac}	1.30	0.45	-9.85	2.22	-3.64

H_2O (O-H)	D_e	17.40	-1.73	2.08	-1.38	1.73
	ΔE_{forw}	10.87	0.38	-1.24	1.93	-4.09
	ΔE_{back}	12.83	0.52	11.57	0.46	1.26
	ΔE_{reac}	-1.95	-0.15	-12.82	1.47	-5.36
C_2H_6 (C-H)	D_e	20.78	-4.53	-1.65	-3.67	0.68
	ΔE_{forw}	8.12	1.51	0.86	2.47	-1.36
	ΔE_{back}	2.19	1.52	8.30	0.30	1.75
	ΔE_{reac}	5.93	-0.01	-7.44	2.17	-3.11
CH_4 (C-H)	D_e	22.05	-4.43	-1.58	-3.86	0.91
	ΔE_{forw}	7.24	1.28	0.51	2.21	-1.07
	ΔE_{back}	1.76	1.52	8.67	0.38	1.77
	ΔE_{reac}	5.47	-0.23	-8.15	1.84	-2.83
NH_3 (N-H)	D_e	25.67	-3.54	3.14	-3.89	0.56
	ΔE_{forw}	17.48	0.57	2.16	0.76	-3.33
	ΔE_{back}	8.65	1.57	11.36	0.85	2.43
	ΔE_{reac}	8.82	-1.00	-9.20	-0.08	-5.75
CH_3Cl (OxIn) (C-Cl)	D_e	21.97	-2.29	-0.13	-2.57	5.24
	ΔE_{forw}	13.27	-2.02	-3.32	-1.44	-4.24
	ΔE_{back}	56.00	-3.98	11.61	-3.66	-1.84
	ΔE_{reac}	-42.73	1.95	-14.92	2.22	-2.40
		MAD	2.03	5.43	1.95	2.48
		MD	-0.86	0.31	-0.12	-0.08
		RMS	2.70	6.97	2.36	3.08

Table 24: Deviation from the CCSD(T)/CBS values in kcal/mol for tested density functionals and HF with dispersion correction.

Molecule (bond activation)	Reaction Step	CCSD(T)	DSD-BLYP-D3	B2GPPYLP-D3	B2PLYP-D3	PWPB95-D3	HF-D3
Benzene (C-H)	D_e	41.41	3.92	2.32	-0.78	-1.91	-21.83
	ΔE_{forw}	14.85	2.36	2.39	1.49	2.04	3.38
	ΔE_{back}	0.49	-2.00	-1.51	-0.88	-0.31	5.37
	ΔE_{reac}	14.36	4.36	3.90	2.37	2.34	-1.98
C_2H_2 (C-H)	D_e	51.25	3.37	2.04	-0.80	-1.23	-20.43
	ΔE_{forw}	11.53	1.96	1.91	1.15	2.03	4.38
	ΔE_{back}	1.39	-3.12	-2.42	-1.78	-0.59	9.84
	ΔE_{reac}	10.14	5.09	4.33	2.93	2.62	-5.46
C_2H_4 (C-H)	D_e	51.99	3.57	2.11	-1.07	-1.74	-21.11
	ΔE_{forw}	27.84	2.18	2.08	0.82	1.55	3.01
	ΔE_{back}	-0.15	-1.44	-1.08	-0.57	-0.21	4.04
	ΔE_{reac}	27.99	3.61	3.16	1.39	1.75	-1.03
C_2H_6 (C-C)	D_e	20.78	0.99	0.27	-1.99	-2.45	-9.91
	ΔE_{forw}	28.05	-0.47	0.20	-0.11	0.45	14.16
	ΔE_{back}	22.71	-1.68	-1.32	-0.48	-0.35	6.82
	ΔE_{reac}	5.34	1.21	1.52	0.37	0.80	7.33
Cyclopropane (C-H)	D_e	19.72	0.77	0.07	-2.03	-2.81	-10.84
	ΔE_{forw}	4.03	-0.52	0.33	0.40	1.19	14.46
	ΔE_{back}	2.72	-2.19	-1.60	-0.80	0.01	6.78
	ΔE_{reac}	1.30	1.68	1.94	1.22	1.19	7.69
H_2O (O-H)	D_e	17.40	1.82	1.28	-0.39	-0.67	-7.33
	ΔE_{forw}	10.87	0.22	0.84	-0.12	0.78	17.89

	ΔE_{back}	12.83	-3.59	-2.62	-2.15	-0.67	14.84
	ΔE_{reac}	-1.95	3.80	3.45	2.02	1.44	3.04
C_2H_6 (C-H)	D_{e}	20.78	0.98	0.26	-1.99	-2.46	-9.88
	ΔE_{forw}	8.12	-0.47	0.31	0.18	1.07	14.52
	ΔE_{back}	2.19	-1.66	-1.26	-0.49	-0.02	4.90
	ΔE_{reac}	5.93	1.19	1.57	0.67	1.09	9.62
CH_4 (C-H)	D_{e}	22.05	0.62	-0.15	-2.18	-2.51	-10.62
	ΔE_{forw}	7.24	-0.32	0.37	0.22	1.00	13.73
	ΔE_{back}	1.76	-1.86	-1.42	-0.62	0.00	5.61
	ΔE_{reac}	5.47	1.55	1.80	0.85	1.01	8.13
NH_3 (N-H)	D_{e}	25.67	1.95	1.18	-1.00	-2.27	-9.28
	ΔE_{forw}	17.48	0.32	0.86	0.14	0.13	15.31
	ΔE_{back}	8.65	-3.26	-2.38	-1.46	-0.21	11.83
	ΔE_{reac}	8.82	3.59	3.25	1.61	0.35	3.49
CH_3Cl (OxIn) (C-Cl)	D_{e}	21.97	3.32	2.55	0.29	-1.20	-11.84
	ΔE_{forw}	13.27	0.03	0.08	-0.86	-1.18	3.02
	ΔE_{back}	56.00	-3.94	-3.72	-4.37	-3.20	7.94
	ΔE_{reac}	-42.73	3.98	3.80	3.51	2.02	-4.92
		MAD	2.12	1.74	1.21	1.27	9.19
		MD	0.80	0.77	-0.13	-0.03	1.87
		RMS	2.52	2.09	1.54	1.54	10.63

2.3 $PdCl_2$ subset

Table 25: Deviation from the CCSD(T)/CBS values in kcal/mol for tested density functionals without dispersion correction.

Molecule (bond activation)	Reaction Step	CCSD(T)	SVWN	BP86	BLYP	PBE
Benzene	D_e	29.96	8.64	-7.75	-14.70	-5.78
(C-H)	ΔE_{forw}	25.47	-9.66	-6.79	-6.00	-6.48
	ΔE_{back}	2.98	-0.13	0.72	1.52	0.35
	ΔE_{reac}	22.49	-9.53	-7.51	-7.52	-6.83
C_2H_4	D_e	35.04	14.60	-1.51	-8.62	0.26
(C-H)	ΔE_{forw}	29.73	-2.20	-0.70	-0.66	-0.39
	ΔE_{back}	1.87	2.96	2.81	3.34	2.51
	ΔE_{reac}	27.86	-5.16	-3.51	-3.99	-2.90
C_2H_6	D_e	8.06	9.34	-3.98	-8.67	-2.43
(C-C)	ΔE_{forw}	22.65	-2.98	.45	2.56	.20
	ΔE_{back}	28.30	-0.13	2.75	4.87	1.78
	ΔE_{reac}	-5.65	-2.85	-2.30	-2.31	-1.58
C_2H_6	D_e	8.06	9.34	-3.98	-8.67	-2.43
(C-H)	ΔE_{forw}	4.98	-6.21	-2.31	-0.17	-2.36
	ΔE_{back}	7.27	0.89	1.76	2.98	1.23
	ΔE_{reac}	-2.29	-7.10	-4.07	-3.15	-3.59
CH_3Cl	D_e	20.64	7.50	-6.15	-10.96	-4.66
(OxIn)	ΔE_{forw}	30.71	-9.73	-6.51	-4.97	-6.50
(C-Cl)	ΔE_{back}	18.78	1.35	1.85	2.80	1.43
	ΔE_{reac}	11.93	-11.08	-8.36	-7.77	-7.92
CH_4	D_e	6.28	10.05	-3.48	-8.61	-2.00
(C-H)	ΔE_{forw}	6.33	-5.76	-2.95	-1.31	-2.95
	ΔE_{back}	5.57	0.05	1.70	3.27	1.05
	ΔE_{reac}	0.77	-5.82	-4.66	-4.59	-4.01
Cyclopropane	D_e	14.40	10.44	-4.91	-10.76	-3.23
(C-H)	ΔE_{forw}	8.68	-7.01	-3.40	-1.56	-3.47
	ΔE_{back}	3.64	-0.16	1.87	3.65	1.27
	ΔE_{reac}	5.04	-6.84	-5.27	-5.21	-4.74
H_2	D_e	13.91	15.27	0.93	-4.53	2.08
(H-H)	ΔE_{forw}	0.19	-3.58	-1.15	0.08	-1.20
	ΔE_{back}	3.14	1.82	1.22	1.42	0.84
	ΔE_{reac}	-2.95	-5.39	-2.37	-1.34	-2.04
H_2O	D_e	21.12	4.41	-6.89	-9.83	-5.79

(O-H)	ΔE_{forw}	47.26	-18.03	-12.47	-9.24	-12.50
	ΔE_{back}	3.68	-0.10	2.65	4.62	2.18
	ΔE_{reac}	43.57	-17.92	-15.12	-13.85	-14.67
		MAD	6.50	4.08	5.28	3.49
		MD	-1.13	-3.04	-3.55	-2.65
		RMS	8.14	5.19	6.49	4.71

Table 26: Deviation from the CCSD(T)/CBS values in kcal/mol for tested density functionals without dispersion correction.

Molecule (bond activation)	Reaction Step	CCSD(T)	B97-d	TPSS	oTPSS	M06-L
Benzene	D_{e}	29.96	-15.55	-5.74	-7.24	-3.98
(C-H)	ΔE_{forw}	25.47	-5.92	-3.51	-3.98	1.49
	ΔE_{back}	2.98	1.22	0.69	1.00	-0.47
	ΔE_{reac}	22.49	-7.14	-4.21	-4.98	1.96
C_2H_4	D_{e}	35.04	-9.43	-0.61	-2.97	-0.94
(C-H)	ΔE_{forw}	29.73	-0.67	1.97	0.06	6.05
	ΔE_{back}	1.87	2.90	2.89	2.87	2.19
	ΔE_{reac}	27.86	-3.57	-0.92	-2.81	3.86
C_2H_6	D_{e}	8.06	-9.88	-3.94	-2.72	-3.28
(C-C)	ΔE_{forw}	22.65	3.03	-.71	.87	1.33
	ΔE_{back}	28.30	4.44	3.40	1.63	-0.64
	ΔE_{reac}	-5.65	-1.41	-4.12	-.76	1.97
C_2H_6	D_{e}	8.06	-9.88	-3.94	-2.72	-3.28
(C-H)	ΔE_{forw}	4.98	-0.82	-1.18	0.26	2.27
	ΔE_{back}	7.27	2.02	1.71	1.18	-0.29
	ΔE_{reac}	-2.29	-2.84	-2.89	-0.92	2.57
CH_3Cl	D_{e}	20.64	-11.75	-4.65	-5.50	-3.36
(OxIn)	ΔE_{forw}	30.71	-3.95	-7.09	-6.62	-3.17
(C-Cl)	ΔE_{back}	18.78	2.05	1.78	1.71	0.06
	ΔE_{reac}	11.93	-6.00	-8.88	-8.32	-3.23
CH_4	D_{e}	6.28	-9.51	-3.53	-2.08	-3.21
(C-H)	ΔE_{forw}	6.33	-1.75	-1.95	-0.29	2.14
	ΔE_{back}	5.57	2.10	1.84	1.18	-0.93
	ΔE_{reac}	0.77	-3.87	-3.80	-1.48	3.06
Cyclopropane	D_{e}	14.40	-11.92	-4.64	-4.72	-3.52
(C-H)	ΔE_{forw}	8.68	-2.10	-1.98	-1.11	1.89
	ΔE_{back}	3.64	2.63	1.84	1.94	0.39
	ΔE_{reac}	5.04	-4.73	-3.82	-3.04	1.50

H_2 (H-H)	D_e	13.91	-4.79	-1.60	1.32	-0.25
	ΔE_{forw}	0.19	-0.39	0.06	-0.04	2.19
	ΔE_{back}	3.14	0.85	0.74	0.00	-2.05
	ΔE_{reac}	-2.95	-1.23	-0.68	-0.04	4.25
H_2O (O-H)	D_e	21.12	-11.31	-4.19	-4.26	-3.09
	ΔE_{forw}	47.26	-8.72	-11.39	-8.82	-3.17
	ΔE_{back}	3.68	2.67	3.28	3.31	1.17
	ΔE_{reac}	43.57	-11.38	-14.66	-12.12	-4.33
		MAD	5.12	3.47	2.91	2.32
		MD	-3.79	-2.35	-1.95	-0.08
		RMS	6.51	4.59	4.04	2.69

Table 27: Deviation from the CCSD(T)/CBS values in kcal/mol for tested density functionals without dispersion correction.

Molecule (bond activation)	Reaction Step	CCSD(T)	B3LYP	BHLYP	PBE0	PW6B95
Benzene (C-H)	D_e	29.96	-13.68	-13.14	-6.77	-7.55
	ΔE_{forw}	25.47	-1.22	7.26	-0.45	1.94
	ΔE_{back}	2.98	0.57	-1.18	-0.78	-0.65
	ΔE_{reac}	22.49	-1.80	8.44	0.33	2.58
C_2H_4 (C-H)	D_e	35.04	-8.79	-10.80	-2.10	-4.36
	ΔE_{forw}	29.73	3.24	9.25	4.39	5.71
	ΔE_{back}	1.87	2.79	1.44	1.75	2.17
	ΔE_{reac}	27.86	0.45	7.81	2.64	3.53
C_2H_6 (C-C)	D_e	8.06	-7.43	-5.82	-2.69	-3.94
	ΔE_{forw}	22.65	4.04	6.62	2.41	1.71
	ΔE_{back}	28.30	0.58	-7.10	-3.29	-4.00
	ΔE_{reac}	-5.65	3.46	13.72	5.70	5.71
C_2H_6 (C-H)	D_e	8.06	-7.43	-5.82	-2.69	-3.94
	ΔE_{forw}	4.98	2.37	7.41	1.16	2.47
	ΔE_{back}	7.27	0.18	-4.69	-2.17	-2.11
	ΔE_{reac}	-2.29	2.19	12.09	3.33	4.58
CH_3Cl (OxIn) (C-Cl)	D_e	20.64	-7.83	-3.67	-2.68	-3.36
	ΔE_{forw}	30.71	0.76	8.79	0.85	0.46
	ΔE_{back}	18.78	0.60	-4.58	-1.36	-2.53
	ΔE_{reac}	11.93	0.16	13.37	2.21	2.99
CH_4 (C-H)	D_e	6.28	-6.90	-4.85	-1.67	-3.26
	ΔE_{forw}	6.33	1.73	7.38	1.01	2.36

	ΔE_{back}	5.57	0.48	-4.16	-2.25	-2.13
	ΔE_{reac}	0.77	1.24	11.54	3.25	4.48
Cyclopropane (C-H)	D_{e}	14.40	-9.88	-9.30	-4.17	-5.41
	ΔE_{forw}	8.68	1.89	8.43	1.10	2.62
	ΔE_{back}	3.64	1.70	-1.37	-0.98	-0.70
	ΔE_{reac}	5.04	0.17	9.79	2.06	3.23
H_2 (H-H)	D_{e}	13.91	-3.61	-3.61	1.57	-1.80
	ΔE_{forw}	0.19	1.26	4.13	0.50	1.38
	ΔE_{back}	3.14	-0.30	-3.51	-1.34	-1.54
	ΔE_{reac}	-2.95	1.55	7.63	1.84	2.92
H_2O (O-H)	D_{e}	21.12	-5.68	0.52	-2.10	-2.59
	ΔE_{forw}	47.26	0.01	14.58	-0.67	1.36
	ΔE_{back}	3.68	2.02	-1.98	-0.81	-0.57
	ΔE_{reac}	43.57	-2.00	16.58	0.15	1.94
		MAD	3.06	7.29	2.09	2.90
		MD	-1.20	2.53	-0.08	0.10
		RMS	4.45	8.34	2.54	3.31

Table 28: Deviation from the CCSD(T)/CBS values in kcal/mol for tested density functionals without dispersion correction.

Molecule (bond activation)	Reaction Step	CCSD(T)	BMK	TPSSh	M05	M05-2X
Benzene (C-H)	D_{e}	29.96	-5.07	-6.27	-12.10	-5.63
	ΔE_{forw}	25.47	4.71	-1.35	5.11	3.85
	ΔE_{back}	2.98	1.53	0.29	-3.17	-0.89
	ΔE_{reac}	22.49	3.18	-1.64	8.28	4.75
C_2H_4 (C-H)	D_{e}	35.04	-3.33	-1.64	-7.51	-4.48
	ΔE_{forw}	29.73	6.54	3.66	9.72	6.37
	ΔE_{back}	1.87	3.46	2.58	-1.01	2.16
	ΔE_{reac}	27.86	3.08	1.08	10.73	4.21
C_2H_6 (C-C)	D_{e}	8.06	-0.12	-3.95	-7.44	-1.18
	ΔE_{forw}	22.65	2.77	0.34	2.50	2.15
	ΔE_{back}	28.30	-4.20	1.25	-9.18	-6.19
	ΔE_{reac}	-5.65	6.97	-0.90	11.68	8.34
C_2H_6 (C-H)	D_{e}	8.06	-0.12	-3.95	-7.44	-1.18
	ΔE_{forw}	4.98	7.14	0.19	5.22	3.95
	ΔE_{back}	7.27	-0.66	0.33	-5.83	-2.91
	ΔE_{reac}	-2.29	7.80	-0.13	11.05	6.86
CH_3Cl	D_{e}	20.64	-0.17	-3.95	-7.61	0.10

(OxIn)	ΔE_{forw}	30.71	3.77	-3.98	3.68	5.20
(C-Cl)	ΔE_{back}	18.78	2.75	0.73	-6.29	-4.21
	ΔE_{reac}	11.93	1.02	-4.72	9.97	9.41
CH_4	D_{e}	6.28	0.65	-3.32	-6.58	-1.07
(C-H)	ΔE_{forw}	6.33	6.71	-0.40	5.00	3.80
	ΔE_{back}	5.57	0.38	0.46	-6.01	-2.86
	ΔE_{reac}	0.77	6.32	-0.87	10.99	6.65
Cyclopropane	D_{e}	14.40	-2.40	-5.03	-8.36	-3.19
(C-H)	ΔE_{forw}	8.68	6.61	-0.26	6.76	4.28
	ΔE_{back}	3.64	1.56	0.95	-3.65	-1.41
	ΔE_{reac}	5.04	5.05	-1.21	10.41	5.69
H_2	D_{e}	13.91	-1.20	-1.42	-6.86	-2.46
(H-H)	ΔE_{forw}	0.19	3.38	0.64	4.58	0.88
	ΔE_{back}	3.14	0.97	-0.15	-4.98	-1.15
	ΔE_{reac}	-2.95	2.40	0.79	9.56	2.03
H_2O	D_{e}	21.12	4.16	-2.95	-6.20	2.66
(O-H)	ΔE_{forw}	47.26	7.26	-6.59	7.09	9.14
	ΔE_{back}	3.68	4.76	2.06	-4.10	-3.50
	ΔE_{reac}	43.57	2.52	-8.64	11.20	12.65
		MAD	3.46	2.19	7.16	4.10
		MD	2.50	-1.33	0.81	1.75
		RMS	4.17	3.03	7.69	4.94

Table 29: Deviation from the CCSD(T)/CBS values in kcal/mol for tested density functionals without dispersion correction.

Molecule (bond activation)	Reaction Step	CCSD(T)	M06	M06-2X	M06-HF	1DH-BLYP
Benzene	D_{e}	29.96	-5.67	-8.99	-4.07	-3.21
(C-H)	ΔE_{forw}	25.47	4.78	4.40	5.97	-3.82
	ΔE_{back}	2.98	-2.04	-2.16	-2.95	1.64
	ΔE_{reac}	22.49	6.82	6.55	8.92	-5.46
C_2H_4	D_{e}	35.04	-3.02	-8.06	-6.47	-0.15
(C-H)	ΔE_{forw}	29.73	8.88	6.93	4.75	2.14
	ΔE_{back}	1.87	0.94	0.97	0.33	4.53
	ΔE_{reac}	27.86	7.93	5.96	4.42	-2.38
C_2H_6	D_{e}	8.06	-3.14	-3.81	0.43	-4.42
(C-C)	ΔE_{forw}	22.65	0.36	2.95	0.81	0.31
	ΔE_{back}	28.30	-7.13	-8.71	-10.32	7.20
	ΔE_{reac}	-5.65	7.49	11.66	11.12	-6.89

C_2H_6 (C-H)	D_e	8.06	-3.14	-3.81	0.43	-4.42
	ΔE_{forw}	4.98	3.88	5.45	3.92	-2.20
	ΔE_{back}	7.27	-3.32	-4.42	-6.63	3.75
	ΔE_{reac}	-2.29	7.20	9.88	10.54	-5.96
CH_3Cl (OxIn) (C-Cl)	D_e	20.64	-3.44	-2.86	3.66	-3.63
	ΔE_{forw}	30.71	-0.22	6.88	9.64	-1.76
	ΔE_{back}	18.78	-5.49	-6.18	-9.22	5.55
	ΔE_{reac}	11.93	5.27	13.06	18.85	-7.31
CH_4 (C-H)	D_e	6.28	-2.80	-3.67	-0.37	-4.08
	ΔE_{forw}	6.33	3.94	5.30	3.38	-2.38
	ΔE_{back}	5.57	-4.10	-4.57	-5.38	3.31
	ΔE_{reac}	0.77	8.03	9.86	8.75	-5.70
Cyclopropane (C-H)	D_e	14.40	-3.50	-6.15	-3.61	-3.92
	ΔE_{forw}	8.68	5.33	5.26	3.87	-3.27
	ΔE_{back}	3.64	-2.10	-2.54	-4.36	2.45
	ΔE_{reac}	5.04	7.43	7.80	8.23	-5.72
H_2 (H-H)	D_e	13.91	-3.15	-6.73	-4.39	-0.82
	ΔE_{forw}	0.19	3.56	1.66	-1.00	-1.52
	ΔE_{back}	3.14	-3.79	-3.12	-1.17	1.72
	ΔE_{reac}	-2.95	7.35	4.78	0.17	-3.24
H_2O (O-H)	D_e	21.12	-3.83	0.28	6.77	-5.00
	ΔE_{forw}	47.26	4.88	11.65	14.55	-5.40
	ΔE_{back}	3.68	-2.98	-4.67	-7.55	3.04
	ΔE_{reac}	43.57	7.87	16.33	22.11	-8.42
		MAD	4.58	6.06	6.09	3.80
		MD	1.09	1.59	2.34	-1.82
		RMS	5.10	6.98	7.87	4.29

Table 30: Deviation from the CCSD(T)/CBS values in kcal/mol for tested density functionals without dispersion correction.

Molecule (bond activation)	Reaction Step	CCSD(T)	1DH-PBE	PBE0-2	PBE0-DH	PTPSS	mPW2PLYP
Benzene (C-H)	D_e	29.96	1.42	1.33	-3.84	-4.52	-5.92
	ΔE_{forw}	25.47	-4.52	-1.71	1.77	-0.94	-0.53
	ΔE_{back}	2.98	1.21	1.25	-1.28	1.12	0.40
	ΔE_{reac}	22.49	-5.72	-2.95	3.04	-2.06	-0.93
C_2H_4 (C-H)	D_e	35.04	4.52	3.52	-0.68	-1.87	-3.04
	ΔE_{forw}	29.73	2.02	3.97	6.10	3.86	4.18

	ΔE_{back}	1.87	4.28	4.41	1.58	4.00	3.19
	ΔE_{reac}	27.86	-2.26	-0.44	4.52	-0.13	0.99
C_2H_6 (C-C)	D_{e}	8.06	-1.62	-1.29	-1.67	-3.73	-4.45
	ΔE_{forw}	22.65	-1.11	-.10	2.23	.91	2.08
	ΔE_{back}	28.30	6.21	4.31	-4.10	3.42	2.44
	ΔE_{reac}	-5.65	-7.32	-4.41	6.33	-2.51	-.36
C_2H_6 (C-H)	D_{e}	8.06	-1.62	-1.29	-1.67	-3.73	-4.45
	ΔE_{forw}	4.98	-3.82	-2.07	1.56	0.44	0.69
	ΔE_{back}	7.27	3.18	2.12	-3.00	2.19	0.90
	ΔE_{reac}	-2.29	-7.00	-4.20	4.56	-1.74	-0.21
CH_3Cl (OxIn) (C-Cl)	D_{e}	20.64	-0.78	0.65	-0.47	-3.63	-3.59
	ΔE_{forw}	30.71	-2.72	0.77	2.43	-1.42	0.37
	ΔE_{back}	18.78	5.25	4.08	-2.05	3.31	1.75
	ΔE_{reac}	11.93	-7.97	-3.31	4.47	-4.73	-1.38
CH_4 (C-H)	D_{e}	6.28	-0.99	-0.42	-0.56	-3.42	-4.10
	ΔE_{forw}	6.33	-3.70	-1.74	1.77	0.17	0.50
	ΔE_{back}	5.57	2.43	1.42	-3.17	2.03	0.77
	ΔE_{reac}	0.77	-6.14	-3.17	4.93	-1.87	-0.28
Cyclopropane (C-H)	D_{e}	14.40	-0.26	-0.35	-2.61	-4.48	-5.15
	ΔE_{forw}	8.68	-4.82	-2.68	1.91	-0.47	0.34
	ΔE_{back}	3.64	1.32	0.83	-1.99	2.00	1.08
	ΔE_{reac}	5.04	-6.15	-3.50	3.90	-2.48	-0.74
H_2 (H-H)	D_{e}	13.91	2.55	2.56	1.98	-1.01	-1.66
	ΔE_{forw}	0.19	-2.52	-1.72	0.78	0.04	0.38
	ΔE_{back}	3.14	1.70	1.08	-2.01	0.56	-0.14
	ΔE_{reac}	-2.95	-4.21	-2.80	2.80	-0.52	0.52
H_2O (O-H)	D_{e}	21.12	-3.47	-1.23	-0.18	-3.27	-3.41
	ΔE_{forw}	47.26	-7.57	-1.71	2.43	-3.34	-0.43
	ΔE_{back}	3.68	1.87	0.95	-2.10	3.16	1.22
	ΔE_{reac}	43.57	-9.43	-2.66	4.53	-6.49	-1.64
		MAD	3.71	2.14	2.64	2.38	1.78
		MD	-1.60	-0.29	0.90	-0.87	-0.57
		RMS	4.39	2.50	3.05	2.84	2.41

Table 31: Deviation from the CCSD(T)/CBS values in kcal/mol for tested density functionals without dispersion correction.

Molecule (bond activation)	Reaction Step	CCSD(T)	B2GPPLYP	B2PLYP	PWPB95	DSD-BLYP
Benzene	D_{e}	29.96	-4.47	-6.31	-4.97	-4.06

(C-H)	ΔE_{forw}	25.47	-1.49	-2.15	0.81	-2.50
	ΔE_{back}	2.98	1.05	0.78	0.02	1.75
	ΔE_{reac}	22.49	-2.53	-2.93	0.79	-4.26
C_2H_4	D_{e}	35.04	-1.76	-3.01	-2.80	-1.37
(C-H)	ΔE_{forw}	29.73	3.66	2.99	4.90	2.97
	ΔE_{back}	1.87	3.91	3.46	3.01	4.65
	ΔE_{reac}	27.86	-0.26	-0.47	1.89	-1.68
C_2H_6	D_{e}	8.06	-4.37	-5.17	-3.88	-4.75
(C-C)	ΔE_{forw}	22.65	1.42	1.60	.87	.98
	ΔE_{back}	28.30	4.37	4.00	-0.60	6.46
	ΔE_{reac}	-5.65	-2.95	-2.40	1.47	-5.48
C_2H_6	D_{e}	8.06	-4.37	-5.17	-3.88	-4.75
(C-H)	ΔE_{forw}	4.98	-0.39	-0.30	1.07	-1.18
	ΔE_{back}	7.27	2.05	1.81	-0.13	3.42
	ΔE_{reac}	-2.29	-2.44	-2.11	1.20	-4.60
CH_3Cl	D_{e}	20.64	-3.21	-4.73	-3.24	-3.66
(OxIn)	ΔE_{forw}	30.71	0.30	-1.06	-0.23	-0.12
(C-Cl)	ΔE_{back}	18.78	3.53	2.94	0.01	5.17
	ΔE_{reac}	11.93	-3.23	-4.00	-0.24	-5.29
CH_4	D_{e}	6.28	-3.91	-4.82	-3.29	-4.33
(C-H)	ΔE_{forw}	6.33	-0.49	-0.59	0.99	-1.33
	ΔE_{back}	5.57	1.77	1.67	-0.26	3.06
	ΔE_{reac}	0.77	-2.27	-2.27	1.24	-4.40
Cyclopropane	D_{e}	14.40	-4.53	-5.66	-4.55	-4.64
(C-H)	ΔE_{forw}	8.68	-1.02	-0.98	0.81	-2.15
	ΔE_{back}	3.64	1.59	1.62	0.21	2.45
	ΔE_{reac}	5.04	-2.61	-2.60	0.60	-4.60
H_2	D_{e}	13.91	-1.14	-1.87	-1.79	-1.40
(H-H)	ΔE_{forw}	0.19	-0.45	-0.21	0.50	-1.05
	ΔE_{back}	3.14	0.66	0.48	-0.48	1.51
	ΔE_{reac}	-2.95	-1.10	-0.69	0.99	-2.57
H_2O	D_{e}	21.12	-3.62	-4.88	-3.01	-4.40
(O-H)	ΔE_{forw}	47.26	-1.40	-3.20	-0.75	-2.74
	ΔE_{back}	3.68	1.91	2.07	0.64	2.99
	ΔE_{reac}	43.57	-3.30	-5.26	-1.39	-5.72
		MAD	2.32	2.67	1.60	3.29
		MD	-0.86	-1.37	-0.37	-1.32
		RMS	2.69	3.17	2.17	3.66

Table 32: Deviation from the CCSD(T)/CBS values in kcal/mol for tested wave-function methods.

Molecule (bond activation)	Reaction Step	CCSD(T)	SOS-MP2	SCS-MP2	MP2	HF
Benzene	D_{e}	29.96	-4.77	-1.73	4.36	-20.34
(C-H)	ΔE_{forw}	25.47	-3.12	-5.44	-10.08	22.52
	ΔE_{back}	2.98	7.37	7.35	7.32	-5.47
	ΔE_{reac}	22.49	-10.49	-12.79	-17.40	27.99
C_2H_4	D_{e}	35.04	-3.88	-0.39	6.58	-22.89
(C-H)	ΔE_{forw}	29.73	1.66	0.44	-2.00	18.07
	ΔE_{back}	1.87	10.36	10.49	10.76	-3.41
	ΔE_{reac}	27.86	-8.70	-10.06	-12.76	21.47
C_2H_6	D_{e}	8.06	-6.73	-5.53	-3.13	-7.44
(C-C)	ΔE_{forw}	22.65	1.16	-.27	-3.13	12.89
	ΔE_{back}	28.30	15.29	17.25	21.17	-22.87
	ΔE_{reac}	-5.65	-14.13	-17.52	-24.30	35.76
C_2H_6	D_{e}	8.06	-6.73	-5.53	-3.13	-7.44
(C-H)	ΔE_{forw}	4.98	-1.05	-3.39	-8.07	18.61
	ΔE_{back}	7.27	10.35	11.20	12.91	-15.10
	ΔE_{reac}	-2.29	-11.40	-14.60	-20.98	33.71
CH_3Cl	D_{e}	20.64	-6.04	-4.76	-2.22	-4.40
(OxIn)	ΔE_{forw}	30.71	3.70	1.77	-2.10	20.54
(C-Cl)	ΔE_{back}	18.78	11.93	13.18	15.68	-17.41
	ΔE_{reac}	11.93	-8.23	-11.41	-17.78	37.95
CH_4	D_{e}	6.28	-6.20	-4.98	-2.53	-6.46
(C-H)	ΔE_{forw}	6.33	-1.49	-3.70	-8.12	18.84
	ΔE_{back}	5.57	10.12	10.62	11.62	-13.57
	ΔE_{reac}	0.77	-11.63	-14.34	-19.75	32.40
Cyclopropane	D_{e}	14.40	-6.98	-4.69	-0.12	-14.53
(C-H)	ΔE_{forw}	8.68	-3.53	-5.98	-10.90	22.05
	ΔE_{back}	3.64	8.41	8.35	8.22	-7.22
	ΔE_{reac}	5.04	-11.93	-14.33	-19.12	29.27
H_2	D_{e}	13.91	-3.62	-1.57	2.52	-10.21
(H-H)	ΔE_{forw}	0.19	-2.29	-3.61	-6.24	11.21
	ΔE_{back}	3.14	6.48	7.00	8.05	-11.26
	ΔE_{reac}	-2.95	-8.77	-10.61	-14.28	22.47
H_2O	D_{e}	21.12	-4.74	-4.92	-5.28	6.00
(O-H)	ΔE_{forw}	47.26	0.34	-3.15	-10.14	39.74
	ΔE_{back}	3.68	9.67	9.35	8.70	-9.53

	ΔE_{reac}	43.57	-9.32	-12.49	-18.83	49.27
		MAD	7.02	7.63	10.01	18.90
		MD	-1.64	-2.24	-3.46	7.81
		RMS	8.02	9.05	11.95	21.90

Table 33: Deviation from the CCSD(T)/CBS values in kcal/mol for tested density functionals with dispersion correction.

Molecule (bond activation)	Reaction Step	CCSD(T)	BP86-D3	PBE-D3	BLYP-D3	B97-D3
Benzene	D_{e}	29.96	1.92	-0.78	-3.70	-4.58
(C-H)	ΔE_{forw}	25.47	-5.16	-5.79	-4.38	-4.74
	ΔE_{back}	2.98	0.72	1.03	1.38	2.40
	ΔE_{reac}	22.49	-5.88	-6.08	-5.76	-5.60
C_2H_4	D_{e}	35.04	3.48	2.95	-2.58	-3.04
(C-H)	ΔE_{forw}	29.73	-1.13	-0.51	-0.75	-0.90
	ΔE_{back}	1.87	2.81	2.39	3.74	2.67
	ΔE_{reac}	27.86	-3.94	-3.17	-4.49	-4.19
C_2H_6	D_{e}	8.06	1.58	-2.01	.32	-2.60
(C-C)	ΔE_{forw}	22.65	-.58	-.47	.94	1.31
	ΔE_{back}	28.30	2.75	1.11	4.53	2.72
	ΔE_{reac}	-5.65	-3.33	-2.07	-3.59	-2.39
C_2H_6	D_{e}	8.06	1.58	0.67	-2.01	-2.60
(C-H)	ΔE_{forw}	4.98	-2.67	-2.46	-0.41	-1.13
	ΔE_{back}	7.27	1.76	1.13	3.21	1.70
	ΔE_{reac}	-2.29	-4.43	-3.78	-3.62	-3.25
CH_3Cl	D_{e}	20.64	-0.69	-1.86	-4.60	-5.38
(OxIn)	ΔE_{forw}	30.71	-7.51	-7.21	-6.65	-5.82
(C-Cl)	ΔE_{back}	18.78	1.85	0.72	2.43	0.18
	ΔE_{reac}	11.93	-9.36	-8.48	-9.07	-7.32
CH_4	D_{e}	6.28	0.62	0.34	-3.53	-3.77
(C-H)	ΔE_{forw}	6.33	-2.94	-2.96	-1.35	-1.89
	ΔE_{back}	5.57	1.70	1.04	3.26	1.96
	ΔE_{reac}	0.77	-4.65	-4.01	-4.62	-3.87
Cyclopropane	D_{e}	14.40	2.01	0.56	-2.51	-3.02
(C-H)	ΔE_{forw}	8.68	-3.13	-3.41	-1.46	-2.01
	ΔE_{back}	3.64	1.87	1.33	3.36	2.73
	ΔE_{reac}	5.04	-4.99	-4.55	-4.82	-4.09
H_2	D_{e}	13.91	2.13	2.88	-2.87	-2.54
(H-H)	ΔE_{forw}	0.19	-1.19	-1.22	0.04	-0.48

	ΔE_{back}	3.14	1.22	0.82	1.46	0.75
	ΔE_{reac}	-2.95	-2.40	-2.07	-1.42	-1.39
H_2O	D_{e}	21.12	-4.49	-4.37	-6.76	-7.65
(O-H)	ΔE_{forw}	47.26	-12.53	-12.59	-9.45	-9.14
	ΔE_{back}	3.68	2.65	2.09	4.56	2.25
	ΔE_{reac}	43.57	-15.18	-14.74	-14.01	-11.65
		MAD	3.52	3.12	3.76	3.44
		MD	-1.82	-2.02	-2.15	-2.40
		RMS	4.74	4.50	4.66	4.22

Table 34: Deviation from the CCSD(T)/CBS values in kcal/mol for tested density functionals with dispersion correction.

Molecule (bond activation)	Reaction Step	CCSD(T)	TPSS-D3	oTPSS-D3	BHLYP-D3	PBE0-D3
Benzene	D_{e}	29.96	1.10	1.84	-5.61	-1.86
(C-H)	ΔE_{forw}	25.47	-2.50	-2.62	8.48	0.27
	ΔE_{back}	2.98	0.63	0.90	-1.18	-0.82
	ΔE_{reac}	22.49	-3.13	-3.52	9.66	1.09
C_2H_4	D_{e}	35.04	2.97	1.84	-6.60	0.43
(C-H)	ΔE_{forw}	29.73	1.81	-0.12	9.00	4.27
	ΔE_{back}	1.87	3.09	3.16	1.44	1.89
	ΔE_{reac}	27.86	-1.28	-3.28	7.57	2.39
C_2H_6	D_{e}	8.06	0.18	2.76	-1.40	0.24
(C-C)	ΔE_{forw}	22.65	-1.58	-0.34	5.60	1.83
	ΔE_{back}	28.30	3.20	1.36	-7.10	-3.40
	ΔE_{reac}	-5.65	-4.79	-1.70	12.71	5.23
C_2H_6	D_{e}	8.06	0.18	2.76	-1.40	0.24
(C-H)	ΔE_{forw}	4.98	-1.30	0.09	7.05	1.08
	ΔE_{back}	7.27	1.84	1.36	-4.69	-2.08
	ΔE_{reac}	-2.29	-3.14	-1.27	11.74	3.16
CH_3Cl	D_{e}	20.64	-0.84	-0.39	0.85	0.03
(OxIn)	ΔE_{forw}	30.71	-8.01	-7.88	7.86	0.23
(C-Cl)	ΔE_{back}	18.78	1.59	1.44	-4.58	-1.48
	ΔE_{reac}	11.93	-9.60	-9.32	12.44	1.71
CH_4	D_{e}	6.28	-0.46	2.03	-1.47	0.48
(C-H)	ΔE_{forw}	6.33	-1.96	-0.30	7.35	1.01
	ΔE_{back}	5.57	1.84	1.17	-4.16	-2.26
	ΔE_{reac}	0.77	-3.80	-1.49	11.51	3.26
Cyclopropane	D_{e}	14.40	0.42	2.02	-3.77	-0.58

(C-H)	ΔE_{forw}	8.68	-1.91	-1.01	8.64	1.16
	ΔE_{back}	3.64	1.68	1.71	-1.36	-1.07
	ΔE_{reac}	5.04	-3.59	-2.73	10.00	2.22
H_2 (H-H)	D_{e}	13.91	-0.60	2.65	-2.58	2.25
	ΔE_{forw}	0.19	0.04	-0.06	4.08	0.49
	ΔE_{back}	3.14	0.76	0.03	-3.51	-1.33
	ΔE_{reac}	-2.95	-0.71	-0.09	7.59	1.82
H_2O (O-H)	D_{e}	21.12	-2.35	-1.80	2.54	-0.82
	ΔE_{forw}	47.26	-11.48	-8.96	14.50	-0.72
	ΔE_{back}	3.68	3.25	3.27	-1.98	-0.82
	ΔE_{reac}	43.57	-14.73	-12.22	16.49	0.11
		MAD	2.84	2.49	6.35	1.50
		MD	-1.48	-0.80	3.49	0.55
		RMS	4.28	3.71	7.56	1.92

Table 35: Deviation from the CCSD(T)/CBS values in kcal/mol for tested density functionals with dispersion correction.

Molecule (bond activation)	Reaction Step	CCSD(T)	B3LYP-D3	BMK-D3	PW6B95-D3	TPSSh-D3
Benzene (C-H)	D_{e}	29.96	-4.53	3.46	-3.97	5.40
	ΔE_{forw}	25.47	0.25	6.32	2.52	0.28
	ΔE_{back}	2.98	0.57	1.56	-0.63	0.31
	ΔE_{reac}	22.49	-0.32	4.76	3.16	0.02
C_2H_4 (C-H)	D_{e}	35.04	-3.77	1.05	-2.61	6.46
	ΔE_{forw}	29.73	2.85	6.60	5.66	4.67
	ΔE_{back}	1.87	2.79	3.74	2.26	3.23
	ΔE_{reac}	27.86	0.05	2.87	3.40	1.44
C_2H_6 (C-C)	D_{e}	8.06	-1.92	4.44	-1.97	2.85
	ΔE_{forw}	22.65	2.96	1.72	1.35	-2.50
	ΔE_{back}	28.30	0.58	-4.22	-4.02	1.36
	ΔE_{reac}	-5.65	2.37	5.94	5.37	-3.87
C_2H_6 (C-H)	D_{e}	8.06	-1.92	4.44	-1.97	2.85
	ΔE_{forw}	4.98	1.97	7.03	2.44	-0.22
	ΔE_{back}	7.27	0.18	-0.44	-2.03	0.76
	ΔE_{reac}	-2.29	1.79	7.47	4.46	-0.97
CH_3Cl (OxIn) (C-Cl)	D_{e}	20.64	-2.52	4.94	-1.34	5.03
	ΔE_{forw}	30.71	-0.32	2.69	0.09	-6.94
	ΔE_{back}	18.78	0.60	2.55	-2.60	0.19
	ΔE_{reac}	11.93	-0.91	0.14	2.69	-7.13

CH_4	D_e	6.28	-2.71	3.96	-1.88	2.54
(C-H)	ΔE_{forw}	6.33	1.70	6.76	2.38	-0.44
	ΔE_{back}	5.57	0.48	0.40	-2.12	0.51
	ΔE_{reac}	0.77	1.21	6.35	4.49	-0.95
Cyclopropane	D_e	14.40	-3.06	3.41	-2.94	3.72
(C-H)	ΔE_{forw}	8.68	2.19	6.72	2.67	-0.05
	ΔE_{back}	3.64	1.71	1.49	-0.64	0.73
	ΔE_{reac}	5.04	0.48	5.23	3.31	-0.78
H_2	D_e	13.91	-2.26	-0.42	-1.43	0.24
(H-H)	ΔE_{forw}	0.19	1.20	3.38	1.38	0.62
	ΔE_{back}	3.14	-0.30	0.99	-1.53	-0.06
	ΔE_{reac}	-2.95	1.49	2.39	2.91	0.68
H_2O	D_e	21.12	-3.16	6.02	-1.79	0.56
(O-H)	ΔE_{forw}	47.26	-0.11	7.25	1.36	-6.96
	ΔE_{back}	3.68	2.02	4.74	-0.58	2.00
	ΔE_{reac}	43.57	-2.12	2.52	1.94	-8.95
		MAD	1.65	3.84	2.44	2.40
		MD	-0.01	3.56	0.55	0.18
		RMS	2.00	4.43	2.76	3.44

Table 36: Deviation from the CCSD(T)/CBS values in kcal/mol for tested density functionals and HF with dispersion correction.

Molecule (bond activation)	Reaction Step	CCSD(T)	DSD-BLYP-D3	B2GPPYLP-D3	B2PLYP-D3	PWPB95-D3	HF-D3
Benzene	D_e	29.96	3.81	2.85	-1.57	-1.84	-2.02
(C-H)	ΔE_{forw}	25.47	-1.79	-0.74	-1.38	1.38	22.25
	ΔE_{back}	2.98	1.77	1.06	0.76	0.04	-6.58
	ΔE_{reac}	22.49	-3.55	-1.80	-2.13	1.34	28.82
C_2H_4	D_e	35.04	4.84	3.81	-0.47	-1.23	-8.34
(C-H)	ΔE_{forw}	29.73	3.21	3.84	2.98	4.92	18.21
	ΔE_{back}	1.87	4.86	4.10	3.62	3.10	-3.13
	ΔE_{reac}	27.86	-1.65	-0.26	-0.64	1.82	21.35
C_2H_6	D_e	8.06	1.22	1.05	-2.43	-2.25	7.12
(C-C)	ΔE_{forw}	22.65	0.15	0.71	0.94	0.53	8.24
	ΔE_{back}	28.30	6.50	4.40	3.93	-0.59	-25.30
	ΔE_{reac}	-5.65	-6.35	-3.69	-2.98	1.12	33.55
C_2H_6	D_e	8.06	1.22	1.05	-2.43	-2.25	7.12
(C-H)	ΔE_{forw}	4.98	-1.29	-0.48	-0.39	1.04	17.93
	ΔE_{back}	7.27	3.58	2.19	1.92	-0.05	-15.58
	ΔE_{reac}	-2.29	-4.87	-2.67	-2.31	1.09	33.50
CH_3Cl	D_e	20.64	2.93	2.75	-1.96	-1.36	9.79
(OxIn)	ΔE_{forw}	30.71	-0.97	-0.42	-1.74	-0.57	15.08
(C-Cl)	ΔE_{back}	18.78	5.02	3.41	2.81	-0.05	-19.80
	ΔE_{reac}	11.93	-5.99	-3.83	-4.54	-0.52	34.88
CH_4	D_e	6.28	1.17	1.00	-2.77	-2.13	6.85
(C-H)	373521 ΔE_{forw}	6.33	-1.32	-0.47	-0.58	1.02	18.72
	ΔE_{back}	5.57	3.08	1.79	1.68	-0.25	-14.50
	ΔE_{reac}	0.77	-4.41	-2.27	-2.27	1.26	33.21
Cyclopropane	D_e	14.40	2.02	1.54	-2.23	-2.46	2.93
(C-H)	ΔE_{forw}	8.68	-2.09	-0.96	-0.93	0.86	22.73
	ΔE_{back}	3.64	2.40	1.55	1.54	0.19	-8.84
	ΔE_{reac}	5.04	-4.50	-2.52	-2.47	0.66	31.57
H_2	D_e	13.91	2.66	2.42	-1.27	-1.53	-3.56
(H-H)	ΔE_{forw}	0.19	-1.05	-0.44	-0.21	0.51	10.91

	ΔE_{back}	3.14	1.53	0.67	0.49	-0.48	-11.09
	ΔE_{reac}	-2.95	-2.59	-1.11	-0.70	0.99	22.00
H_2O (O-H)	D_{e}	21.12	0.29	0.53	-3.67	-2.37	14.88
	ΔE_{forw}	47.26	-2.79	-1.42	-3.25	-0.74	37.29
	ΔE_{back}	3.68	2.98	1.90	2.05	0.64	-10.36
	ΔE_{reac}	43.57	-5.76	-3.31	-5.29	-1.37	47.66
		MAD	2.95	1.92	2.04	1.24	17.66
		MD	0.12	0.45	-0.77	0.01	10.49
		RMS	3.42	2.28	2.37	1.57	20.97

2.4 Ni subset

Table 37: Deviation from the CCSD(T)/CBS values in kcal/mol for tested density functionals without dispersion correction.

Molecule (bond activation)	Reaction Step	CCSD(T)	SVWN	BP86	BLYP	PBE
Benzene	D_e	54.10	27.39	9.17	-0.57	10.96
(C-H)	ΔE_{forw}	18.18	-0.45	-2.52	-4.53	-1.60
	ΔE_{back}	4.62	-3.94	-1.21	0.58	-1.74
	ΔE_{reac}	13.56	3.49	-1.31	-5.11	0.14
BH_3	D_e	67.70	29.50	9.45	-0.06	11.44
(B-H)	ΔE_{forw}	11.38	-4.20	-5.46	-6.32	-4.86
	ΔE_{back}	2.19	-0.48	0.32	1.15	-0.18
	ΔE_{reac}	9.19	-3.72	-5.78	-7.47	-4.68
C_2H_4	D_e	66.55	26.92	10.83	2.30	12.32
(C-H)	ΔE_{forw}	30.44	-2.74	-3.03	-4.10	-2.38
	ΔE_{back}	4.08	-3.74	-1.22	0.49	-1.74
	ΔE_{reac}	26.36	1.00	-1.81	-4.59	-0.65
C_2H_6	D_e	29.25	31.74	9.46	-1.15	11.07
(C-C)	ΔE_{forw}	10.43	-1.20	-3.72	-5.44	-3.44
	ΔE_{back}	27.03	-9.97	-1.40	3.10	-2.61
	ΔE_{reac}	-16.60	8.77	-2.31	-8.53	-0.83
C_2H_6	D_e	31.11	26.49	10.31	3.45	11.27
(C-H)	ΔE_{forw}	4.28	-1.27	-0.84	-0.40	-0.83
	ΔE_{back}	10.34	-4.32	-0.49	1.76	-1.15
	ΔE_{reac}	-6.06	3.05	-0.35	-2.17	0.31
CH_4	D_e	30.77	26.62	10.72	3.98	11.67
(C-H)	ΔE_{forw}	4.09	-1.04	-0.88	-0.51	-0.84
	ΔE_{back}	9.43	-5.16	-1.01	1.26	-1.68
	ΔE_{reac}	-5.34	4.12	0.13	-1.76	0.83
Cyclopropane	D_e	29.73	26.19	10.04	3.11	11.00
(C-H)	ΔE_{forw}	-0.31	-0.86	-0.81	-0.27	-0.77
	ΔE_{back}	7.25	-4.21	-0.48	1.86	-1.10
	ΔE_{reac}	-7.56	3.35	-0.32	-2.13	0.33
H_2	D_e	43.06	23.58	9.99	4.20	10.47
(H-H)	ΔE_{forw}	0.60	-0.64	-0.52	-0.44	-0.48
	ΔE_{back}	2.29	-3.08	-1.06	0.16	-1.43
	ΔE_{reac}	-1.69	2.45	0.54	-0.60	0.95
NH_3	D_e	56.75	15.88	3.55	-0.29	3.79
(N-H)	ΔE_{forw}	25.74	-11.04	-8.37	-6.42	-8.52

	ΔE_{back}	14.87	-5.81	-1.57	0.40	-2.06
	ΔE_{reac}	10.88	-5.23	-6.81	-6.83	-6.47
		MAD	9.27	3.83	2.71	4.07
		MD	5.21	0.87	-1.16	1.29
		RMS	13.76	5.39	3.60	5.88

Table 38: Deviation from the CCSD(T)/CBS values in kcal/mol for tested density functionals without dispersion correction.

Molecule (bond activation)	Reaction Step	CCSD(T)	B97-d	TPSS	oTPSS	M06-L
Benzene	D_{e}	54.10	-2.57	11.02	10.10	-3.64
(C-H)	ΔE_{forw}	18.18	-3.50	-0.63	-2.39	1.29
	ΔE_{back}	4.62	-1.45	-0.12	1.48	-0.40
	ΔE_{reac}	13.56	-2.05	-0.51	-3.87	1.69
BH_3	D_{e}	67.70	-2.79	7.73	11.14	-7.78
(B-H)	ΔE_{forw}	11.38	-5.08	-5.03	-4.30	-1.38
	ΔE_{back}	2.19	-0.71	0.61	1.33	1.57
	ΔE_{reac}	9.19	-4.36	-5.63	-5.63	-2.95
C_2H_4	D_{e}	66.55	-0.42	12.61	13.23	-1.15
(C-H)	ΔE_{forw}	30.44	-3.60	-1.18	-1.78	1.82
	ΔE_{back}	4.08	-1.48	-0.27	1.34	-0.22
	ΔE_{reac}	26.36	-2.11	-0.91	-3.12	2.04
C_2H_6	D_{e}	29.25	-3.77	8.98	10.11	-7.06
(C-C)	ΔE_{forw}	10.43	-4.20	-4.12	-3.05	-2.61
	ΔE_{back}	27.03	0.26	0.53	3.72	1.52
	ΔE_{reac}	-16.60	-4.47	-4.65	-6.77	-4.12
C_2H_6	D_{e}	31.11	0.67	9.41	10.90	-4.36
(C-H)	ΔE_{forw}	4.28	-0.05	-0.86	-0.59	1.88
	ΔE_{back}	10.34	-0.87	1.27	3.13	1.42
	ΔE_{reac}	-6.06	0.82	-2.13	-3.73	0.46
CH_4	D_{e}	30.77	1.17	9.79	11.34	-4.32
(C-H)	ΔE_{forw}	4.09	-0.25	-0.95	-0.69	1.76
	ΔE_{back}	9.43	-1.36	1.04	3.02	1.20
	ΔE_{reac}	-5.34	1.12	-1.99	-3.71	0.55
Cyclopropane	D_{e}	29.73	0.51	9.26	10.53	-4.35
(C-H)	ΔE_{forw}	-0.31	0.22	-1.01	-0.52	1.76
	ΔE_{back}	7.25	-0.69	1.37	3.75	2.04
	ΔE_{reac}	-7.56	0.91	-2.38	-4.27	-0.27
H_2	D_{e}	43.06	1.90	8.13	11.82	-5.05

(H-H)	ΔE_{forw}	0.60	-0.21	-0.68	-0.95	-0.64
	ΔE_{back}	2.29	-1.82	0.37	1.69	0.63
	ΔE_{reac}	-1.69	1.62	-1.05	-2.63	-1.26
NH_3	D_{e}	56.75	-4.78	4.11	4.21	-10.34
(N-H)	ΔE_{forw}	25.74	-6.36	-8.80	-9.35	-5.59
	ΔE_{back}	14.87	-2.18	1.07	3.08	2.17
	ΔE_{reac}	10.88	-4.19	-9.88	-12.44	-7.77
		MAD	2.07	3.89	5.16	2.75
		MD	-1.56	0.96	1.28	-1.43
		RMS	2.65	5.46	6.50	3.66

Table 39: Deviation from the CCSD(T)/CBS values in kcal/mol for tested density functionals without dispersion correction.

Molecule (bond activation)	Reaction Step	CCSD(T)	B3LYP	BHLYP	PBE0	PW6B95
Benzene	D_{e}	54.10	-7.74	-19.24	-0.16	-3.73
(C-H)	ΔE_{forw}	18.18	-2.86	-2.10	-0.15	0.73
	ΔE_{back}	4.62	1.32	5.81	-0.34	0.36
	ΔE_{reac}	13.56	-4.18	-7.91	0.19	0.37
BH_3	D_{e}	67.70	-8.05	-21.25	-0.85	-4.62
(B-H)	ΔE_{forw}	11.38	-3.19	0.61	-1.26	-0.85
	ΔE_{back}	2.19	-0.33	-0.89	-1.91	-1.68
	ΔE_{reac}	9.19	-2.86	1.50	0.65	0.83
C_2H_4	D_{e}	66.55	-4.27	-14.37	2.53	-2.00
(C-H)	ΔE_{forw}	30.44	-1.50	0.97	0.73	0.82
	ΔE_{back}	4.08	1.36	6.02	-0.22	0.50
	ΔE_{reac}	26.36	-2.85	-5.05	0.95	0.32
C_2H_6	D_{e}	29.25	-8.68	-21.74	-0.96	-4.89
(C-C)	ΔE_{forw}	10.43	-3.27	-0.93	-1.07	-1.84
	ΔE_{back}	27.03	4.04	11.00	0.19	0.39
	ΔE_{reac}	-16.60	-7.31	-11.94	-1.26	-2.22
C_2H_6	D_{e}	31.11	-1.71	-9.87	2.82	-0.16
(C-H)	ΔE_{forw}	4.28	2.23	6.01	2.20	3.12
	ΔE_{back}	10.34	1.87	6.22	-0.23	0.22
	ΔE_{reac}	-6.06	0.36	-0.22	2.44	2.91
CH_4	D_{e}	30.77	-1.16	-9.34	3.31	0.24
(C-H)	ΔE_{forw}	4.09	1.92	5.47	1.92	2.85
	ΔE_{back}	9.43	1.79	6.78	-0.17	0.37
	ΔE_{reac}	-5.34	0.13	-1.31	2.09	2.48

Cyclopropane (C-H)	D_e	29.73	-2.18	-10.41	2.42	-0.46
	ΔE_{forw}	-0.31	2.48	6.30	2.35	3.53
	ΔE_{back}	7.25	2.52	7.83	0.49	1.09
	ΔE_{reac}	-7.56	-0.04	-1.54	1.86	2.44
H_2 (H-H)	D_e	43.06	-1.53	-10.99	1.92	-2.50
	ΔE_{forw}	0.60	-0.21	-0.20	-0.25	-0.18
	ΔE_{back}	2.29	0.97	5.22	-0.01	0.60
	ΔE_{reac}	-1.69	-1.17	-5.42	-0.24	-0.78
NH_3 (N-H)	D_e	56.75	-4.53	-8.64	-2.92	-5.44
	ΔE_{forw}	25.74	-3.19	2.78	-4.48	-3.64
	ΔE_{back}	14.87	1.16	6.93	-0.28	0.20
	ΔE_{reac}	10.88	-4.35	-4.15	-4.21	-3.85
		MAD	2.76	6.86	1.39	1.76
		MD	-1.53	-2.45	0.22	-0.41
		RMS	3.53	8.83	1.83	2.33

Table 40: Deviation from the CCSD(T)/CBS values in kcal/mol for tested density functionals without dispersion correction.

		CCSD(T)	BMK	TPSSh	M05	M05-2X
Benzene (C-H)	D_e	54.10	-3.63	6.20	-7.12	-13.58
	ΔE_{forw}	18.18	1.59	-0.10	2.85	1.80
	ΔE_{back}	4.62	3.46	0.22	-3.93	2.07
	ΔE_{reac}	13.56	-1.87	-0.32	6.77	-0.27
BH_3 (B-H)	D_e	67.70	-10.02	3.00	-12.71	-18.89
	ΔE_{forw}	11.38	-1.48	-3.56	0.58	0.34
	ΔE_{back}	2.19	-0.66	-0.22	-3.80	-2.98
	ΔE_{reac}	9.19	-0.82	-3.34	4.39	3.33
C_2H_4 (C-H)	D_e	66.55	-0.47	8.30	-4.24	-13.32
	ΔE_{forw}	30.44	3.37	-0.02	4.03	1.33
	ΔE_{back}	4.08	3.71	0.13	-3.61	2.54
	ΔE_{reac}	26.36	-0.34	-0.15	7.63	-1.20
C_2H_6 (C-C)	D_e	29.25	-6.47	4.02	-9.76	-17.62
	ΔE_{forw}	10.43	-2.71	-3.15	-2.65	-2.02
	ΔE_{back}	27.03	5.89	1.12	-4.73	3.62
	ΔE_{reac}	-16.60	-8.60	-4.27	2.08	-5.64
C_2H_6 (C-H)	D_e	31.11	0.52	5.99	-4.78	-7.90
	ΔE_{forw}	4.28	4.92	0.43	3.67	5.50
	ΔE_{back}	10.34	4.17	1.31	-4.56	1.16

	ΔE_{reac}	-6.06	0.75	-0.87	8.23	4.34
CH_4 (C-H)	D_{e}	30.77	0.84	6.41	-4.22	-7.76
	ΔE_{forw}	4.09	4.67	0.24	3.36	5.17
	ΔE_{back}	9.43	4.47	1.28	-4.75	1.81
	ΔE_{reac}	-5.34	0.20	-1.04	8.11	3.36
Cyclopropane (C-H)	D_{e}	29.73	0.23	5.73	-4.86	-8.37
	ΔE_{forw}	-0.31	4.71	0.35	3.68	5.73
	ΔE_{back}	7.25	4.79	1.70	-4.15	2.47
	ΔE_{reac}	-7.56	-0.08	-1.35	7.82	3.27
H_2 (H-H)	D_{e}	43.06	-5.28	4.83	-8.42	-12.39
	ΔE_{forw}	0.60	-0.56	-0.57	0.14	-0.08
	ΔE_{back}	2.29	3.89	0.73	-3.60	1.91
	ΔE_{reac}	-1.69	-4.45	-1.30	3.75	-1.99
NH_3 (N-H)	D_{e}	56.75	-0.69	1.14	-11.58	-10.44
	ΔE_{forw}	25.74	0.04	-7.07	-2.02	-0.18
	ΔE_{back}	14.87	4.80	1.37	-4.01	1.82
	ΔE_{reac}	10.88	-4.77	-8.44	1.99	-2.01
		MAD	3.05	2.51	4.96	4.95
		MD	0.11	0.52	-1.12	-2.09
		RMS	3.94	3.56	5.69	6.93

Table 41: Deviation from the CCSD(T)/CBS values in kcal/mol for tested density functionals without dispersion correction.

Molecule (bond activation)	Reaction Step	CCSD(T)	M06	M06-2X	M06-HF	1DH-BLYP
Benzene (C-H)	D_{e}	54.10	-0.32	-18.25	-31.31	12.78
	ΔE_{forw}	18.18	2.25	1.67	-0.03	10.12
	ΔE_{back}	4.62	-2.63	2.38	8.63	-18.52
	ΔE_{reac}	13.56	4.88	-0.71	-8.66	28.64
BH_3 (B-H)	D_{e}	67.70	-6.56	-24.25	-41.45	12.39
	ΔE_{forw}	11.38	0.60	1.49	-1.14	0.16
	ΔE_{back}	2.19	-1.47	-2.83	-5.24	-10.63
	ΔE_{reac}	9.19	2.07	4.31	4.11	10.79
C_2H_4 (C-H)	D_{e}	66.55	0.37	-17.40	-32.07	6.03
	ΔE_{forw}	30.44	1.95	1.75	-1.12	3.87
	ΔE_{back}	4.08	-2.00	2.90	8.77	-17.83
	ΔE_{reac}	26.36	3.95	-1.15	-9.89	21.70
C_2H_6 (C-C)	D_{e}	29.25	-2.11	-23.16	-39.71	13.93
	ΔE_{forw}	10.43	-2.67	-3.42	-3.58	0.90

	ΔE_{back}	27.03	-3.56	4.64	15.52	-30.35
	ΔE_{reac}	-16.60	0.90	-8.07	-19.10	31.25
C_2H_6	D_{e}	31.11	3.67	-11.94	-26.65	5.85
(C-H)	ΔE_{forw}	4.28	4.16	5.89	4.38	-3.55
	ΔE_{back}	10.34	-3.50	1.92	8.75	-26.98
	ΔE_{reac}	-6.06	7.65	3.97	-4.37	23.43
CH_4	D_{e}	30.77	3.81	-11.79	-26.43	7.58
(C-H)	ΔE_{forw}	4.09	4.06	5.54	3.94	0.53
	ΔE_{back}	9.43	-3.52	2.41	9.82	-23.68
	ΔE_{reac}	-5.34	7.57	3.14	-5.87	24.22
Cyclopropane	D_{e}	29.73	2.94	-12.26	-26.72	7.23
(C-H)	ΔE_{forw}	-0.31	3.88	6.31	4.46	1.04
	ΔE_{back}	7.25	-2.94	3.56	11.16	-23.89
	ΔE_{reac}	-7.56	6.82	2.75	-6.70	24.93
H_2	D_{e}	43.06	-1.14	-18.57	-30.09	-4.71
(H-H)	ΔE_{forw}	0.60	0.24	-0.12	-0.44	4.02
	ΔE_{back}	2.29	-2.23	2.28	6.81	-16.54
	ΔE_{reac}	-1.69	2.47	-2.41	-7.25	20.57
NH_3	D_{e}	56.75	-1.80	-13.07	-22.04	-20.74
(N-H)	ΔE_{forw}	25.74	-1.08	1.13	0.82	-16.19
	ΔE_{back}	14.87	-3.28	2.94	10.92	-18.56
	ΔE_{reac}	10.88	2.18	-1.81	-10.11	2.37
		MAD	2.98	6.45	12.72	14.13
		MD	0.71	-3.06	-7.27	1.73
		RMS	3.53	9.19	17.14	16.96

Table 42: Deviation from the CCSD(T)/CBS values in kcal/mol for tested density functionals without dispersion correction.

Molecule (bond activation)	Reaction Step	CCSD(T)	1DH-PBE	PBE0-2	PBE0-DH	PTPSS	mPW2PLYP
Benzene	D_{e}	54.10	19.66	13.48	3.03	12.74	4.83
(C-H)	ΔE_{forw}	18.18	12.38	13.95	3.61	4.98	4.68
	ΔE_{back}	4.62	-21.35	-21.94	-3.86	-7.46	-8.79
	ΔE_{reac}	13.56	33.73	35.90	7.47	12.45	13.47
BH_3	D_{e}	67.70	19.15	12.80	1.34	12.33	4.11
(B-H)	ΔE_{forw}	11.38	0.76	4.01	0.17	-1.81	-1.00
	ΔE_{back}	2.19	-12.73	-17.81	-3.40	-2.13	-4.45
	ΔE_{reac}	9.19	13.50	21.81	3.57	0.31	3.45
C_2H_4	D_{e}	66.55	11.15	4.27	3.16	10.79	2.59

(C-H)	ΔE_{forw}	30.44	4.75	6.13	2.71	2.38	1.89
	ΔE_{back}	4.08	-20.53	-20.68	-3.61	-7.31	-8.45
	ΔE_{reac}	26.36	25.28	26.82	6.32	9.69	10.35
C_2H_6	D_{e}	29.25	21.19	15.17	1.71	11.33	4.65
(C-C)	ΔE_{forw}	10.43	2.11	3.29	0.71	-0.61	-0.37
	ΔE_{back}	27.03	-36.20	-38.07	-6.03	-11.16	-13.68
	ΔE_{reac}	-16.60	38.31	41.35	6.74	10.55	13.31
C_2H_6	D_{e}	31.11	10.48	5.00	1.84	8.84	2.93
(C-H)	ΔE_{forw}	4.28	-3.74	-2.62	-1.44	-3.01	-2.14
	ΔE_{back}	10.34	-30.77	-32.14	-7.45	-11.79	-14.05
	ΔE_{reac}	-6.06	27.03	29.52	6.01	8.78	11.90
CH_4	D_{e}	30.77	12.08	6.14	4.00	11.03	5.06
(C-H)	ΔE_{forw}	4.09	0.52	0.79	2.93	1.63	2.19
	ΔE_{back}	9.43	-27.29	-29.04	-4.74	-8.72	-11.14
	ΔE_{reac}	-5.34	27.81	29.83	7.68	10.35	13.33
Cyclopropane	D_{e}	29.73	11.86	6.38	3.22	10.22	4.31
(C-H)	ΔE_{forw}	-0.31	0.85	1.97	3.15	1.58	2.45
	ΔE_{back}	7.25	-27.68	-29.05	-4.36	-8.70	-10.96
	ΔE_{reac}	-7.56	28.53	31.02	7.51	10.28	13.40
H_2	D_{e}	43.06	-0.21	-6.15	-8.29	-1.26	-7.23
(H-H)	ΔE_{forw}	0.60	4.01	4.28	6.42	5.12	5.68
	ΔE_{back}	2.29	-20.15	-21.90	2.40	-1.58	-4.00
	ΔE_{reac}	-1.69	24.16	26.18	4.03	6.70	9.68
NH_3	D_{e}	56.75	-19.97	-33.55	-5.06	-1.10	-9.07
(N-H)	ΔE_{forw}	25.74	-18.34	-21.76	-4.44	-8.00	-7.62
	ΔE_{back}	14.87	-21.95	-18.96	-4.03	-7.94	-9.36
	ΔE_{reac}	10.88	3.60	-2.80	-0.42	-0.07	1.73
		MAD	17.25	17.61	3.86	7.10	6.88
		MD	3.10	1.73	1.17	2.61	1.03
		RMS	20.24	21.32	4.36	8.24	8.10

Table 43: Deviation from the CCSD(T)/CBS values in kcal/mol for tested density functionals without dispersion correction.

Molecule (bond activation)	Reaction Step	CCSD(T)	B2GPPLYP	B2PLYP	PWPB95	DSD-BLYP
Benzene	D_{e}	54.10	7.43	7.30	4.94	9.55
(C-H)	ΔE_{forw}	18.18	8.24	5.44	3.97	10.00
	ΔE_{back}	4.62	-14.35	-10.36	-4.90	-17.76
	ΔE_{reac}	13.56	22.59	15.80	8.87	27.75

BH_3 (B-H)	D_e	67.70	6.62	6.46	3.87	9.01
	ΔE_{forw}	11.38	0.42	-1.65	-1.09	1.04
	ΔE_{back}	2.19	-9.01	-4.33	-2.34	-11.69
	ΔE_{reac}	9.19	9.43	2.68	1.25	12.73
C_2H_4 (C-H)	D_e	66.55	2.54	4.29	3.90	3.18
	ΔE_{forw}	30.44	3.51	1.88	2.05	4.21
	ΔE_{back}	4.08	-13.73	-10.08	-4.76	-16.97
	ΔE_{reac}	26.36	17.24	11.97	6.81	21.18
C_2H_6 (C-C)	D_e	29.25	7.83	7.03	3.40	10.36
	ΔE_{forw}	10.43	0.75	-0.59	-0.65	1.14
	ΔE_{back}	27.03	-23.32	-16.04	-7.82	-29.06
	ΔE_{reac}	-16.60	24.07	15.45	7.17	30.20
C_2H_6 (C-H)	D_e	31.11	4.48	5.81	5.20	4.69
	ΔE_{forw}	4.28	1.67	1.65	2.74	0.72
	ΔE_{back}	10.34	-18.18	-12.53	-5.87	-22.57
	ΔE_{reac}	-6.06	19.85	14.18	8.61	23.29
CH_4 (C-H)	D_e	30.77	4.82	6.09	5.37	5.11
	ΔE_{forw}	4.09	1.64	1.58	2.56	0.84
	ΔE_{back}	9.43	-18.47	-12.95	-5.89	-22.89
	ΔE_{reac}	-5.34	20.12	14.53	8.45	23.72
Cyclopropane (C-H)	D_e	29.73	4.34	5.36	4.59	4.83
	ΔE_{forw}	-0.31	2.17	1.72	2.71	1.59
	ΔE_{back}	7.25	-18.46	-12.92	-5.73	-22.98
	ΔE_{reac}	-7.56	20.63	14.64	8.44	24.57
H_2 (H-H)	D_e	43.06	5.25	5.86	2.96	6.56
	ΔE_{forw}	0.60	2.16	1.39	0.29	2.65
	ΔE_{back}	2.29	-15.37	-11.09	-4.62	-19.00
	ΔE_{reac}	-1.69	17.53	12.48	4.91	21.65
NH_3 (N-H)	D_e	56.75	-18.41	-9.01	-3.88	-24.08
	ΔE_{forw}	25.74	-12.63	-8.68	-5.45	-16.33
	ΔE_{back}	14.87	-14.22	-11.34	-5.98	-16.84
	ΔE_{reac}	10.88	1.58	2.65	0.52	0.50
		MAD	10.92	7.99	4.52	13.37
		MD	1.13	1.24	1.24	1.14
		RMS	13.26	9.36	5.09	16.37

Table 44: Deviation from the CCSD(T)/CBS values in kcal/mol for tested wavefunction methods.

Molecule (bond activation)	Reaction Step	CCSD(T)	SOS-MP2	SCS-MP2	MP2	HF
Benzene	D_e	54.10	7.43	11.83	20.63	-44.95
(C-H)	ΔE_{forw}	18.18	24.20	28.11	35.92	-8.10
	ΔE_{back}	4.62	-30.66	-36.18	-47.20	24.65
	ΔE_{reac}	13.56	54.87	64.28	83.12	-32.75
BH_3	D_e	67.70	7.84	13.17	23.85	-50.30
(B-H)	ΔE_{forw}	11.38	11.89	13.49	16.71	1.71
	ΔE_{back}	2.19	-33.48	-38.14	-47.46	7.71
	ΔE_{reac}	9.19	45.37	51.64	64.17	-6.00
C_2H_4	D_e	66.55	-10.59	-9.75	-8.06	-34.38
(C-H)	ΔE_{forw}	30.44	9.49	10.37	12.15	0.07
	ΔE_{back}	4.08	-28.00	-33.18	-43.54	24.42
	ΔE_{reac}	26.36	37.49	43.55	55.68	-24.35
C_2H_6	D_e	29.25	9.24	15.97	29.44	-52.29
(C-C)	ΔE_{forw}	10.43	3.58	4.54	6.47	0.18
	ΔE_{back}	27.03	-57.57	-68.71	-90.97	42.18
	ΔE_{reac}	-16.60	61.15	73.25	97.44	-42.00
C_2H_6	D_e	31.11	-36.55	-39.39	-45.07	-23.93
(C-H)	ΔE_{forw}	4.28	-30.59	-35.27	-44.62	14.69
	ΔE_{back}	10.34	-43.09	-50.50	-65.31	28.47
	ΔE_{reac}	-6.06	12.50	15.23	20.69	-13.78
CH_4	D_e	30.77	-33.09	-35.62	-40.67	-23.86
(C-H)	ΔE_{forw}	4.09	-28.20	-32.58	-41.34	13.48
	ΔE_{back}	9.43	-43.33	-50.94	-66.17	29.97
	ΔE_{reac}	-5.34	15.13	18.36	24.83	-16.49
Cyclopropane	D_e	29.73	-30.54	-32.60	-36.73	-25.76
(C-H)	ΔE_{forw}	-0.31	-21.80	-25.56	-33.08	13.27
	ΔE_{back}	7.25	-40.63	-48.10	-63.05	31.74
	ΔE_{reac}	-7.56	18.83	22.54	29.97	-18.47
H_2	D_e	43.06	3.07	5.16	9.33	-30.57
(H-H)	ΔE_{forw}	0.60	6.95	8.38	11.24	-2.06
	ΔE_{back}	2.29	-38.06	-44.44	-57.22	23.63
	ΔE_{reac}	-1.69	45.01	52.82	68.45	-25.69
NH_3	D_e	56.75	-74.82	-83.72	-101.51	0.77
(N-H)	ΔE_{forw}	25.74	-28.81	-33.71	-43.51	22.88
	ΔE_{back}	14.87	-8.46	-12.15	-19.53	27.15
	ΔE_{reac}	10.88	-20.36	-21.57	-24.00	-4.28
		MAD	28.13	32.91	42.48	21.86
		MD	-7.35	-7.76	-8.58	-4.81

		RMS	33.19	38.52	49.35	26.02

Table 45: Deviation from the CCSD(T)/CBS values in kcal/mol for tested density functionals with dispersion correction.

Molecule (bond activation)	Reaction Step	CCSD(T)	BP86-D3	PBE-D3	BLYP-D3	B97-D3
Benzene	D_e	54.10	13.80	13.24	4.93	2.69
(C-H)	ΔE_{forw}	18.18	-1.84	-1.38	-3.81	-3.02
	ΔE_{back}	4.62	-1.21	-1.53	0.38	-0.97
	ΔE_{reac}	13.56	-0.63	0.46	-4.19	-1.18
BH_3	D_e	67.70	11.20	12.39	2.18	-0.37
(B-H)	ΔE_{forw}	11.38	-5.44	-4.87	-6.36	-5.06
	ΔE_{back}	2.19	0.32	-0.19	1.04	-0.70
	ΔE_{reac}	9.19	-5.76	-4.65	-7.40	-4.13
C_2H_4	D_e	66.55	13.13	13.53	5.21	2.62
(C-H)	ΔE_{forw}	30.44	-3.30	-2.57	-4.47	-4.02
	ΔE_{back}	4.08	-1.22	-1.92	0.36	-1.91
	ΔE_{reac}	26.36	-2.07	-0.76	-4.82	-2.23
C_2H_6	D_e	29.25	12.65	12.81	2.95	0.72
(C-C)	ΔE_{forw}	10.43	-3.20	-3.39	-5.32	-3.96
	ΔE_{back}	27.03	-1.40	-2.56	2.32	0.50
	ΔE_{reac}	-16.60	-1.79	-0.42	-7.64	-2.89
C_2H_6	D_e	31.11	13.14	12.80	6.97	4.51
(C-H)	ΔE_{forw}	4.28	-0.86	-0.86	-0.50	-0.12
	ΔE_{back}	10.34	-0.49	-1.18	1.63	-0.94
	ΔE_{reac}	-6.06	-0.37	0.35	-2.13	1.13
CH_4	D_e	30.77	12.70	12.78	7.61	4.14
(C-H)	ΔE_{forw}	4.09	-0.86	-0.85	3.88	-0.24
	ΔE_{back}	9.43	-1.01	-1.68	3.35	-1.36
	ΔE_{reac}	-5.34	0.15	0.87	0.53	1.38
Cyclopropane	D_e	29.73	13.08	12.60	5.72	4.34
(C-H)	ΔE_{forw}	-0.31	-0.97	-0.95	-5.17	-0.30
	ΔE_{back}	7.25	-0.48	-1.29	-0.65	-1.21
	ΔE_{reac}	-7.56	-0.49	0.29	-4.52	0.98
H_2	D_e	43.06	10.69	10.89	5.16	3.10
(H-H)	ΔE_{forw}	0.60	-0.52	-0.48	-0.44	-0.21
	ΔE_{back}	2.29	-1.06	-1.43	0.16	-1.83
	ΔE_{reac}	-1.69	0.54	0.95	-0.60	1.62
NH_3	D_e	56.75	5.38	4.83	2.13	-1.95

(N-H)	ΔE_{forw}	25.74	-8.19	-8.43	-6.21	-6.10
	ΔE_{back}	14.87	-1.57	-1.96	0.33	-1.92
	ΔE_{reac}	10.88	-6.62	-6.35	-6.55	-3.69
		MAD	4.39	4.40	6.81	2.17
		MD	1.54	1.64	-0.77	-0.63
		RMS	6.49	6.48	5.92	2.67

Table 46: Deviation from the CCSD(T)/CBS values in kcal/mol for tested density functionals with dispersion correction.

Molecule (bond activation)	Reaction Step	CCSD(T)	TPSS-D3	oTPSS-D3	BHLYP-D3	PBE0-D3
Benzene	D_{e}	54.10	14.13	14.33	-15.18	2.02
(C-H)	ΔE_{forw}	18.18	-0.34	-1.95	-1.33	0.04
	ΔE_{back}	4.62	-0.24	1.32	5.81	-0.42
	ΔE_{reac}	13.56	-0.10	-3.27	-7.15	0.46
BH_3	D_{e}	67.70	8.96	12.84	-19.61	0
(B-H)	ΔE_{forw}	11.38	-5.05	-4.33	0.63	-1.28
	ΔE_{back}	2.19	0.55	1.25	-0.89	-1.94
	ΔE_{reac}	9.19	-5.60	-5.58	1.52	0.66
C_2H_4	D_{e}	66.55	14.21	15.43	-12.24	3.64
(C-H)	ΔE_{forw}	30.44	-1.43	-2.10	0.80	0.55
	ΔE_{back}	4.08	-0.34	1.24	6.02	-0.27
	ΔE_{reac}	26.36	-1.09	-3.34	-5.22	0.82
C_2H_6	D_{e}	29.25	11.26	13.23	-18.84	0.61
(C-C)	ΔE_{forw}	10.43	-4.06	-2.96	-0.43	-1.03
	ΔE_{back}	27.03	0.11	3.14	11.00	-0.07
	ΔE_{reac}	-16.60	-4.17	-6.10	-11.44	-0.96
C_2H_6	D_{e}	31.11	11.44	13.65	-7.42	4.23
(C-H)	ΔE_{forw}	4.28	-0.91	-0.66	5.98	2.17
	ΔE_{back}	10.34	1.20	3.03	6.22	-0.27
	ΔE_{reac}	-6.06	-2.11	-3.69	-0.25	2.45
CH_4	D_{e}	30.77	11.23	13.31	-7.53	4.30
(C-H)	ΔE_{forw}	4.09	-0.96	-0.70	5.50	1.92
	ΔE_{back}	9.43	1.00	2.96	6.78	-0.19
	ΔE_{reac}	-1.96	-3.66	-1.29	2.11	
Cyclopropane	D_{e}	29.73	11.40	13.42	-7.87	3.92
(C-H)	ΔE_{forw}	-0.31	-1.25	-0.86	6.11	2.20
	ΔE_{back}	7.25	1.21	3.52	7.83	0.39
	ΔE_{reac}	-7.56	-2.45	-4.39	-1.73	1.81

H_2	D_e	43.06	8.66	12.55	-10.34	2.28
(H-H)	ΔE_{forw}	0.60	-0.68	-0.95	-0.20	-0.25
	ΔE_{back}	2.29	0.37	1.68	5.22	-0.01
	ΔE_{reac}	-1.69	-1.05	-2.63	-5.42	-0.24
NH_3	D_e	56.75	5.45	6.05	-6.97	-2.01
(N-H)	ΔE_{forw}	25.74	-8.67	-9.18	2.95	-4.40
	ΔE_{back}	14.87	1.04	3.03	6.93	-0.30
		10.88	-9.73	-12.23	-3.98	-4.11
		MAD	4.29	5.68	6.24	1.51
		MD	1.39	1.87	-1.83	0.52
		RMS	6.21	7.44	7.88	2.05

Table 47: Deviation from the CCSD(T)/CBS values in kcal/mol for tested density functionals with dispersion correction.

		CCSD(T)	B3LYP-D3	BMK-D3	PW6B95-D3	TPSSh-D3
Benzene	D_e	54.10	-3.12	0.94	-2.14	15.20
(C-H)	ΔE_{forw}	18.18	-2.08	2.39	0.89	2.12
	ΔE_{back}	4.62	1.33	3.41	0.37	0.19
	ΔE_{reac}	13.56	-3.41	-1.03	0.52	1.92
BH_3	D_e	67.70	-6.19	-8.36	-4.01	7.48
(B-H)	ΔE_{forw}	11.38	-3.14	-1.52	-0.87	-3.67
	ΔE_{back}	2.19	-0.33	-0.68	-1.69	-0.30
	ΔE_{reac}	9.19	-2.81	-0.83	0.83	-3.36
C_2H_4	D_e	66.55	-1.84	1.70	-1.22	13.68
(C-H)	ΔE_{forw}	30.44	-1.70	3.11	0.69	0.04
	ΔE_{back}	4.08	1.36	3.70	0.49	0.18
	ΔE_{reac}	26.36	-3.06	-0.59	0.20	-0.14
C_2H_6	D_e	29.25	-5.28	-3.61	-3.82	10.83
(C-H)	ΔE_{forw}	10.43	-2.56	-2.69	-1.82	-3.11
	ΔE_{back}	27.03	4.04	5.63	0.29	0.34
	ΔE_{reac}	-16.60	-6.60	-8.32	-2.11	-3.45
C_2H_6	D_e	31.11	1.21	3.02	0.80	11.00
(C-H)	ΔE_{forw}	4.28	2.25	4.85	3.11	0.22
	ΔE_{back}	10.34	1.87	4.18	0.22	1.33
	ΔE_{reac}	-6.06	0.38	0.67	2.89	-1.12
CH_4	D_e	30.77	0.99	2.57	0.89	10.59
(C-H)	ΔE_{forw}	4.09	1.98	4.66	2.85	0.17
	ΔE_{back}	9.43	1.79	4.47	0.37	1.24

	ΔE_{reac}	-5.34	0.19	0.19	2.48	-1.07
Cyclopropane	D_{e}	29.73	0.85	2.95	0.61	10.64
(C-H)	ΔE_{forw}	-0.31	2.32	4.36	3.43	-0.72
	ΔE_{back}	7.25	2.52	4.67	1.05	1.20
	ΔE_{reac}	-7.56	-0.20	-0.31	2.38	-1.92
H_2	D_{e}	43.06	-0.74	-4.71	-2.28	6.27
(H-H)	ΔE_{forw}	0.60	-0.20	-0.56	-0.18	-0.57
	ΔE_{back}	2.29	0.97	3.89	0.60	0.73
	ΔE_{reac}	-1.69	-1.17	-4.45	-0.78	-1.31
NH_3	D_{e}	56.75	-2.53	0.87	-4.86	4.86
(N-H)	ΔE_{forw}	25.74	-2.96	0.18	-3.61	-6.81
	ΔE_{back}	14.87	1.15	4.80	0.19	1.39
	ΔE_{reac}	10.88	-4.12	-4.63	-3.81	-8.21
		MAD	2.20	3.04	1.65	3.82
		MD	-0.80	0.69	-0.20	1.83
		RMS	2.70	3.70	2.12	5.74

Table 48: Deviation from the CCSD(T)/CBS values in kcal/mol for tested density functionals and HF with dispersion correction.

Molecule (bond activation)	Reaction Step	CCSD(T)	DSD-BLYP-D3	B2GPPYLP-D3	B2PLYP-D3	PWPB95-D3	HF-D3
Benzene	D_{e}	54.10	12.46	10.10	9.74	6.57	-30.10
(C-H)	ΔE_{forw}	18.18	10.68	8.85	5.80	4.24	-6.06
	ΔE_{back}	4.62	-17.77	-14.36	-10.42	-4.91	23.73
	ΔE_{reac}	13.56	28.45	23.21	16.22	9.15	-29.79
BH_3	D_{e}	67.70	10.27	7.71	7.41	4.46	-40.34
(B-H)	ΔE_{forw}	11.38	1.01	0.39	-1.67	-1.10	2.07
	ΔE_{back}	2.19	-11.71	-9.03	-4.36	-2.35	6.95
	ΔE_{reac}	9.19	12.72	9.42	2.69	1.24	-4.87
C_2H_4	D_{e}	66.55	4.74	3.91	5.53	4.65	-23.91
(C-H)	ΔE_{forw}	30.44	4.15	3.43	1.73	1.95	-0.70
	ΔE_{back}	4.08	-16.96	-13.72	-10.11	-4.76	23.55
	ΔE_{reac}	26.36	21.11	17.15	11.85	6.71	-24.25
C_2H_6	D_{e}	29.25	12.36	9.58	8.73	4.39	-36.53
(C-C)	ΔE_{forw}	10.43	1.15	0.76	-0.56	-0.65	1.20
	ΔE_{back}	27.03	-29.25	-23.47	-16.28	-7.88	38.04
	ΔE_{reac}	-16.60	30.41	24.23	15.72	7.23	-36.84
C_2H_6	D_{e}	31.11	6.24	5.89	7.27	6.06	-12.15
(C-H)	ΔE_{forw}	4.28	0.66	1.61	1.61	2.72	14.70
	ΔE_{back}	10.34	-22.56	-18.16	-12.55	-5.86	27.10
	ΔE_{reac}	-6.06	23.21	19.78	14.16	8.58	-12.40
CH_4	D_{e}	30.77	6.32	5.87	7.14	5.96	-13.12
(C-H)	ΔE_{forw}	4.09	0.82	1.63	1.57	2.56	13.65
	ΔE_{back}	9.43	-22.89	-18.48	-12.97	-5.89	28.64
	ΔE_{reac}	-5.34	23.71	20.11	14.54	8.45	-14.99
Cyclopropane	D_{e}	29.73	6.42	5.81	6.91	5.56	-14.58
(C-H)	ΔE_{forw}	-0.31	1.26	1.89	1.51	2.60	12.13
	ΔE_{back}	7.25	-23.10	-18.56	-13.03	-5.76	29.49
	ΔE_{reac}	-7.56	24.36	20.45	14.54	8.36	-17.36
H_2	D_{e}	43.06	6.96	5.59	6.23	3.14	-25.32
(H-H)	ΔE_{forw}	0.60	2.65	2.16	1.39	0.29	-2.08
	ΔE_{back}	2.29	-19.01	-15.37	-11.09	-4.62	23.48
	ΔE_{reac}	-1.69	21.65	17.53	12.48	4.91	-25.55
NH_3	D_{e}	56.75	-23.00	-17.47	-8.04	-3.35	10.60
(N-H)	ΔE_{forw}	25.74	-16.24	-12.55	-8.59	-5.40	23.40
	ΔE_{back}	14.87	-16.83	-14.21	-11.35	-5.98	26.20
	ΔE_{reac}	10.88	0.58	1.65	2.75	0.57	-2.81
		MAD	13.71	11.22	8.29	4.69	18.85

	MD	1.53	1.48	1.57	1.44	-1.91
	RMS	16.54	13.42	9.60	5.26	21.95
